# Unraveling the Dynamics of Omicron (BA.1, BA.2, and BA.5) Waves and Emergence of the Deltacton Variant: Genomic Epidemiology of the SARS-CoV-2 Epidemic in Cyprus (Oct 2021–Oct 2022)

**DOI:** 10.3390/v15091933

**Published:** 2023-09-15

**Authors:** Andreas C. Chrysostomou, Bram Vrancken, Christos Haralambous, Maria Alexandrou, Ioanna Gregoriou, Marios Ioannides, Costakis Ioannou, Olga Kalakouta, Christos Karagiannis, Markella Marcou, Christina Masia, Michail Mendris, Panagiotis Papastergiou, Philippos C. Patsalis, Despo Pieridou, Christos Shammas, Dora C. Stylianou, Barbara Zinieri, Philippe Lemey, The COMESSAR Network, Leondios G. Kostrikis

**Affiliations:** 1 Department of Biological Sciences, University of Cyprus, Aglantzia, 2109 Nicosia, Cyprus; 2 Department of Microbiology, Immunology and Transplantation, Rega Institute, KU Leuven, 3000 Leuven, Belgium; 3 Spatial Epidemiology Lab (SpELL), Université Libre de Bruxelles, 1050 Bruxelles, Belgium; 4 Unit for Surveillance and Control of Communicable Diseases, Ministry of Health, 1148 Nicosia, Cyprus; 5 Microbiology Department, Larnaca General Hospital, 6301 Larnaca, Cyprus; 6 Medicover Genetics, 2409 Nicosia, Cyprus; 7 Medical Laboratory of Ammochostos General Hospital, Ammochostos General Hospital, 5310 Paralimni, Cyprus; 8 Microbiology Department, Nicosia General Hospital, 2029 Nicosia, Cyprus; 9 Department of Microbiology, Archbishop Makarios III Hospital, 2012 Nicosia, Cyprus; 10 Microbiology Department, Limassol General Hospital, 4131 Limassol, Cyprus; 11 Medical School, University of Nicosia, 2417 Nicosia, Cyprus; 12 S.C.I.N.A. Bioanalysis Sciomedical Centre Ltd., 4040 Limassol, Cyprus; 13 Microbiology Department, Paphos General Hospital, Achepans, 8026 Paphos, Cyprus; 14 Cyprus Academy of Sciences, Letters, and Arts, 60-68 Phaneromenis Street, 1011 Nicosia, Cyprus

**Keywords:** SARS-CoV-2, COVID-19, genomic epidemiology, Cyprus

## Abstract

Commencing in December 2019 with the emergence of severe acute respiratory syndrome coronavirus 2 (SARS-CoV-2), three years of the coronavirus disease 2019 (COVID-19) pandemic have transpired. The virus has consistently demonstrated a tendency for evolutionary adaptation, resulting in mutations that impact both immune evasion and transmissibility. This ongoing process has led to successive waves of infections. This study offers a comprehensive assessment spanning genetic, phylogenetic, phylodynamic, and phylogeographic dimensions, focused on the trajectory of the SARS-CoV-2 epidemic in Cyprus. Based on a dataset comprising 4700 viral genomic sequences obtained from affected individuals between October 2021 and October 2022, our analysis is presented. Over this timeframe, a total of 167 distinct lineages and sublineages emerged, including variants such as Delta and Omicron (1, 2, and 5). Notably, during the fifth wave of infections, Omicron subvariants 1 and 2 gained prominence, followed by the ascendancy of Omicron 5 in the subsequent sixth wave. Additionally, during the fifth wave (December 2021–January 2022), a unique set of Delta sequences with genetic mutations associated with Omicron variant 1, dubbed “Deltacron”, was identified. The emergence of this phenomenon initially evoked skepticism, characterized by concerns primarily centered around contamination or coinfection as plausible etiological contributors. These hypotheses were predominantly disseminated through unsubstantiated assertions within the realms of social and mass media, lacking concurrent scientific evidence to validate their claims. Nevertheless, the exhaustive molecular analyses presented in this study have demonstrated that such occurrences would likely lead to a frameshift mutation—a genetic aberration conspicuously absent in our provided sequences. This substantiates the accuracy of our initial assertion while refuting contamination or coinfection as potential etiologies. Comparable observations on a global scale dispelled doubt, eventually leading to the recognition of Delta-Omicron variants by the scientific community and their subsequent monitoring by the World Health Organization (WHO). As our investigation delved deeper into the intricate dynamics of the SARS-CoV-2 epidemic in Cyprus, a discernible pattern emerged, highlighting the major role of international connections in shaping the virus’s local trajectory. Notably, the United States and the United Kingdom were the central conduits governing the entry and exit of the virus to and from Cyprus. Moreover, notable migratory routes included nations such as Greece, South Korea, France, Germany, Brazil, Spain, Australia, Denmark, Sweden, and Italy. These empirical findings underscore that the spread of SARS-CoV-2 within Cyprus was markedly influenced by the influx of new, highly transmissible variants, triggering successive waves of infection. This investigation elucidates the emergence of new waves of infection subsequent to the advent of highly contagious and transmissible viral variants, notably characterized by an abundance of mutations localized within the spike protein. Notably, this discovery decisively contradicts the hitherto hypothesis of seasonal fluctuations in the virus’s epidemiological dynamics. This study emphasizes the importance of meticulously examining molecular genetics alongside virus migration patterns within a specific region. Past experiences also emphasize the substantial evolutionary potential of viruses such as SARS-CoV-2, underscoring the need for sustained vigilance. However, as the pandemic’s dynamics continue to evolve, a balanced approach between caution and resilience becomes paramount. This ethos encourages an approach founded on informed prudence and self-preservation, guided by public health authorities, rather than enduring apprehension. Such an approach empowers societies to adapt and progress, fostering a poised confidence rooted in well-founded adaptation.

## 1. Introduction

Since severe acute respiratory syndrome coronavirus 2 (SARS-CoV-2) emerged in China (December 2019), it has rapidly spread worldwide, resulting in the coronavirus disease 2019 (COVID-19) pandemic [1]. As the COVID-19 pandemic has progressed, it has caused devastating global socioeconomic consequences, and as of August 2023, it has resulted in approximately 676.6 million positive SARS-CoV-2 cases and 6.9 million deaths [2,3,4]. In Cyprus, which is the focal point of this study, there have been approximately more than 650 thousand cases and 1300 deaths thus far [3,4]. This heavy toll of SARS-CoV-2 on humanity is underlined by high-risk variants that have evolved throughout this pandemic with mutations that confer high transmissibility, immune evasion, and pathogenicity, causing large-scale outbreaks upon their emergence [5,6,7].

Thus far, five high-risk SARS-CoV-2 variants, including their sublineages, have been denoted as variants of concern (VOCs) by the World Health Organization (WHO): Alpha (B.1.1.7), Beta (B.1.351), Gamma (P.1), Delta (B.1.617.2), and Omicron (B.1.1.529) [5]. Upon the emergence of each of these VOCs, they rapidly outcompeted other circulating lineages, resulting in the onset of successive SARS-CoV-2 epidemic waves. These waves manifested as substantial increases in new infections that peaked before subsequently declining [8,9].

A striking example of this pattern was the Omicron variant. Emerging in November 2021 in South Africa and Botswana, the Omicron variant rapidly rose to global dominance [10]. Omicron was identified as having greater transmissibility than other variants, surpassing even the Delta variant, which had previously been considered the most transmissible [5,10,11,12]. Unlike other variants, Omicron encompassed a substantially larger number of mutations within the S gene—more than 30 mutations—compared to the wild type [13]. Additionally, the Omicron variant displayed considerable variability, giving rise to several subvariant families designated BA.1, BA.2, BA.3, BA.4, and BA.5, each further divided into numerous sublineages [14]. Consistent with the previously described pattern, most of these Omicron subvariants emerged as more infectious strains that outcompeted those in circulation at the time of their introduction. BA.1 became the first Omicron subvariant to achieve global dominance [14]. Subsequently, BA.1 was displaced by BA.2, which was later overtaken by BA.5. [14]. A complex mix of variants, colloquially termed “variant soup”, then emerged. This mixture included a plethora of Omicron BA.5 and BA.2 sublineages and recombinants, such as BQ.1, BF.7, BA.2.75, and XBB, along with their derivatives BQ.1.1, XBB.1.5, and XBB.1.16 [15,16,17]. This “variant soup” is believed to be contributing to the shift of the SARS-CoV-2 pandemic into a “wavelet” era, marked by a smaller number of milder infections [17].

The evolution of the pandemic has been meticulously documented, largely due to the efforts of molecular epidemiology studies. Conducted on both local and global scales, these studies have facilitated the tracking of SARS-CoV-2 transmission, the identification of viral mutations, and the spatiotemporal mapping of the virus’s progression. The insights gained have been instrumental in informing public health responses to the pandemic [18,19,20,21,22,23]. In Cyprus, the impact of SARS-CoV-2 has been diligently studied since the early stages of the COVID-19 pandemic from April 2020 to October 2021 [24,25]. During this period, four consecutive waves of SARS-CoV-2 infections were identified, represented by specific lineages. The first wave was represented by B.1.1 (specifically B.1.1.29), prevalent from April to June 2020, and the second wave, with B.1.258 and sublineages, was from September 2020 to January 2021. The waves that followed were represented by VOCs, with the third wave being characterized by the Alpha variant and sublineages, prevalent from February to May 2021, while the fourth wave was characterized by the Delta variant and sublineages, prevalent from June to September 2021 [24,25].

In this study, we conducted comprehensive genomic epidemiological analyses to examine SARS-CoV-2 infections in Cyprus from October 2021 to October 2022. Our goal was to identify the dominant lineages circulating within the country, understand the mutations they carried, and elucidate the spatiotemporal patterns of these lineages. During this period, the Omicron variant was responsible for the fifth and sixth waves of SARS-CoV-2 infections. Specifically, the BA.1 and BA.2 subvariants of Omicron were prevalent during the fifth wave and BA.5 was prevalent during the sixth wave. Additionally, most SARS-CoV-2 imports and exports were primarily to and from the United States of America (USA) and the United Kingdom (UK), although many other migration links were found, such as Greece, South Korea, France, Germany, Brazil, Spain, Australia, Denmark, Sweden, and Italy. This study underscores the importance of analyzing the impact of the SARS-CoV-2 infection in Cyprus. Our findings reinforce that the emergence of new, distinct, and frequently greater infectious lineages is the primary factor propelling waves of infection. While substantial progress has been achieved in combating COVID-19, the ongoing threat of new variants necessitates continued vigilance to prevent future outbreaks.

## 2. Materials and Methods

### 2.1. Sample Collection, RNA Extraction, and SARS-CoV-2 Real-Time RT-PCR

Sample collection, RNA extraction, and SARS-CoV-2 real-time RT-PCR were performed as explained in our previously published studies [24,25]. In brief, nasopharyngeal and/or oropharyngeal swab samples in transport medium were collected or received by medical facilities situated in Cyprus for diagnostic purposes. RNA extraction was then performed for the identification of SARS-CoV-2-positive samples using real-time RT-PCR. Paphos General Hospital and Bioiatriki Healthcare Group/Yiannoukas Medical Laboratories Ltd. were new contributors to the present study. Paphos General Hospital used the same automated protocol as Nicosia General Hospital, as described in [24]. Bioiatriki Healthcare Group/Yiannoukas Medical Laboratories Ltd. used disposable virus specimen collection tube kits (Shandong Chengwu Medical Products Factory, Chengwu County, Shandong Province, China) encompassing nasopharyngeal swabs and tubes containing sterile viral transport medium. RNA was extracted using the TanBead Nucleic Acis Extraction Kit (Taiwan Advanced Nanotech Inc., Taoyuan, Taiwan) on the Smart LabAssist-32 instrument (Taiwan Advanced Nanotech Inc., Taoyuan, Taiwan). SARS-CoV-2-positive samples were identified using real-time RT-PCR with the SARS-CoV-2 RT-PCR test kit targeting the SARS-CoV-2 N, E, and ORF1ab genes (ACON Biotech, San Diego, CA, USA) on the QuantStudio 5 Real-Time PCR instrument (Thermo Fisher Scientific, Waltham, MA, USA).

### 2.2. Next-Generation Sequencing (NGS)

The next-generation sequencing (NGS) was performed by Medicover Genetics (previously NIPD Genetics), which employed the COVIDSeq Assay (Illumina Inc., San Diego, CA, USA) with ARTIC PCR primers (ARTIC V4.1 since 27 January 2022), which were described in detail in our previously published studies [24,25].

### 2.3. Sequences Used in This Study

The sequences utilized in this prospective investigation were obtained from the molecular epidemiological surveillance study conducted by the Laboratory of Biotechnology and Molecular Virology at the University of Cyprus (BMV UCY), in conjunction with the Cyprus Ministry of Health and members of the Cypriot Comprehensive Molecular Epidemiological Study on SARS-CoV-2 (COMESSAR) Network. Within the framework of this collaborative effort, BMV UCY received a total of 5962 whole-genome SARS-CoV-2 sequences derived from individuals infected in Cyprus during the period spanning from 22 October 2021 to 31 October 2022.

Out of the initial pool of 5962 SARS-CoV-2 sequences, 1233 sequences were excluded based on a stringent quality control protocol. Initially, the Pangolin webtool (versions v1.14 and 1.17 for Pangolin-data and versions 4.1.1 and 4.1.3 for Pangolin) was employed to assess the genomic sequences (https://pangolin.cog-uk.io/, accessed on 20 September 2022 and 16 December 2022) [26]. This initial step resulted in the removal of 256 sequences. Subsequently, the remaining sequences that received successful lineage classification were subjected to further quality evaluation using the Nextclade webtool (versions 2.5.0 and 2.9.1) (https://clades.nextstrain.org/Nextclade, accessed on 20 September 2022 and 16 December 2022) [27]. Through this process, an additional 977 sequences of suboptimal quality were eliminated. Consequently, only sequences classified as “good quality” under the “qc.overallStatus” parameter were retained for the subsequent analyses in this investigation [27]. This meticulous approach was undertaken to mitigate potential misinterpretations arising from artifacts introduced during sequencing and assembly, encompassing issues like ambiguous nucleotides, data gaps, frameshifts, and premature stop codons [27].

Furthermore, 20 sequences were excluded due to missing chronological information, and an additional 9 sequences were identified as duplicates and consequently removed. As a result, the comprehensive analyses were conducted on a refined dataset comprising 4700 sequences that successfully surpassed the established benchmarks for quality control. These curated sequences will be made accessible upon publication of the manuscript through the GISAID database [28]. The dataset encompassing these 4700 sequences was derived from 11 distinct medical facilities situated in Cyprus, where rigorous SARS-CoV-2 testing was conducted. The distribution of sequences is as follows: 3056 sequences originated from Medicover Genetics; 561 sequences were obtained from Limassol General Hospital; 366 sequences were sourced from Ammochostos General Hospital; Nicosia General Hospital contributed 336 sequences; 212 sequences were collected from S.C.I.N.A. Bioanalysis Sciomedical Centre Ltd.; Synlab Cyprus provided 93 sequences; Archbishop Makarios III Hospital contributed 51 sequences; Larnaca General Hospital contributed 10 sequences; Bioiatriki Healthcare Group/Yiannoukas Medical Laboratories Ltd. contributed 7 sequences; Mygene Molecular Diagnostics Ltd. and Paphos General Hospital each contributed 4 sequences. All sequencing processes were executed by Medicover Genetics, which accounts for the comprehensive set of 4700 sequences.

For the ethical conduct of this research, the Cyprus National Bioethics Committee approved (EEBK 21.1.04.43.01) the analysis of these sequences. To ensure the preservation of anonymity, all sequences were assigned a novel laboratory code (double-coded) upon receipt from the BMV UCY laboratory. The handling of these sequences adhered meticulously to the pertinent guidelines and regulations stipulated by the Cyprus National Bioethics Committee.

### 2.4. Bioinformatic Analysis

#### 2.4.1. Lineage Classification and Mutation Calling

As explained in our previous publication [24], lineage classification was performed using the Pangolin webtool (Pangolin-data versions v1.14 and 1.17, Pangolin versions 4.1.1 and 4.1.3) (https://pangolin.cog-uk.io/, accessed on 20 September 2022 and 16 December 2022) [26]. Mutations in the sequences in this study were identified using Nextclade webtool versions 2.5.0 and 2.9.1 (https://clades.nextstrain.org/Nextclade accessed on 20 September 2022 and 16 December 2022) [27].

#### 2.4.2. Dataset Compilation and Time-Scaled Migration Histories

**Figure 1:**
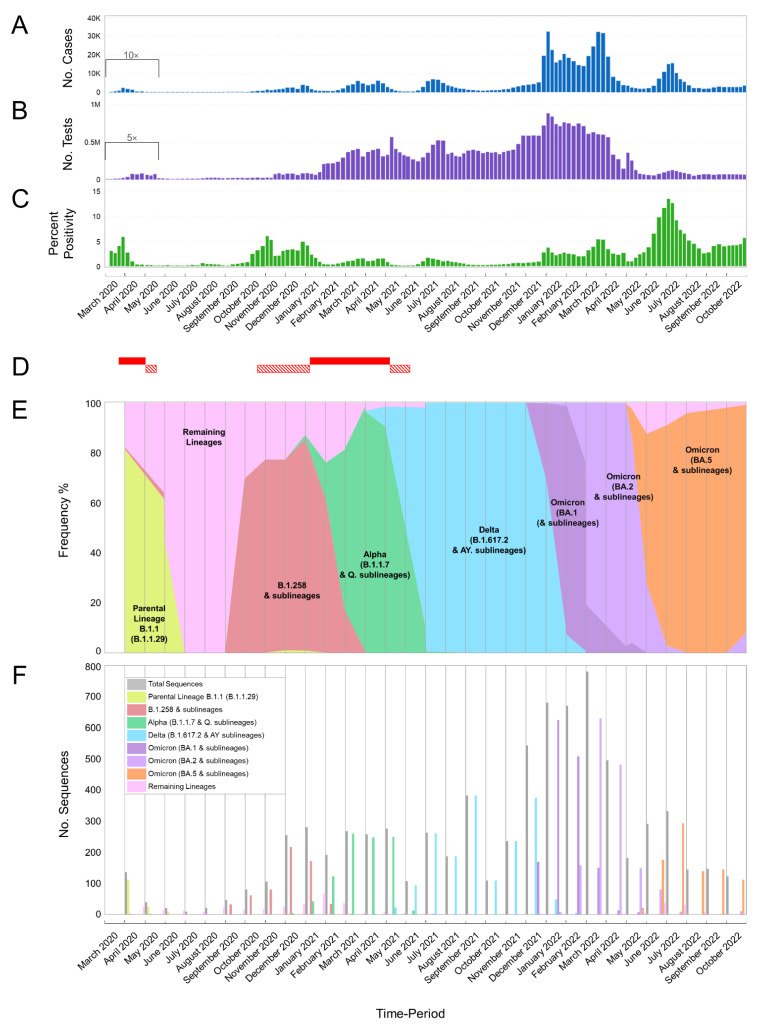
This study encompasses an extensive analysis of SARS-CoV-2 cases, testing, percent positivity, and dominant lineages in Cyprus spanning from March 2020 to October 2022. Specifically, the current investigation is focused on the timeframe from October 2021 to October 2022, while our prior research encompassed the period between April 2020 and October 2021 [24,25]. The inclusion of the previous timeframes serves the purpose of maintaining continuity and illustrating the evolution of SARS-CoV-2 infections in Cyprus. Importantly, there is no overlap in data collection between the earlier studies and the present one. (**A**) Depicts the weekly count of positive SARS-CoV-2 cases. Dark blue columns represent the number of positive cases, with values beneath the black bracket (covering the period from 1 March 2020 to 17 May 2020) being multiplied by 10 for enhanced visibility. (**B**) Presents the total number of SARS-CoV-2 tests, encompassing PCR and rapid tests, conducted weekly in Cyprus. Purple columns represent the test count, with values beneath the black bracket (covering the period from 1 March 2020 to 17 May 2020) being multiplied by 5 for enhanced visibility. (**C**) Illustrates the calculated percent positivity of SARS-CoV-2 testing weekly. The green columns represent the percent positivity values. (**D**) Highlights periods of lockdown and partial lockdown in Cyprus. Red-filled horizontal rectangles indicate full lockdown periods, while horizontal rectangles with diagonal red lines denote partial lockdowns. The initial lockdown occurred from 24 March 2020 to 3 May 2020, and the second lockdown occurred from 10 January 2021 to 9 May 2021. The first partial lockdown spanned from 4 May 2020 to 20 May 2020; the second from 23 October 2020 to 9 January 2021; and the third from 10 May 2021 to 10 June 2021 (information sourced from the Ministry of Health). Brackets underneath (**A**–**C**) group weeks into approximately 1-month periods. (**E**,**F**) Depicts the frequency (proportion) and the number of sequences for the most prevalent lineages in Cyprus per month, respectively. Specifically, the sequences for lineages B.1.1.29 (parental lineage B.1.1), B.1.258 and sublineages, Alpha (B.1.1.7 and Q. sublineages), Delta (B.1.617.2 and AY. sublineages), Omicron 1 (BA.1 and sublineages), Omicron 2 (BA.2 and sublineages), and Omicron 5 (BA.5 and sublineages) are represented in bright green, red, green, light blue, purple, lilac, and orange, respectively. The numbers of sequences categorized under “Remaining Lineages” are displayed in pink, calculated by excluding the monthly sequences of lineages B.1.1.29 (parental lineage B.1.1), B.1.258 and sublineages, Alpha (B.1.1.7 and Q. sublineages), Delta (B.1.617.2 and AY. sublineages), Omicron 1 (BA.1 and sublineages), Omicron 2 (BA.2 and sublineages), and Omicron 5 (BA.5 and sublineages) from the total number of sequences indicated in gray. Recombinant lineages such as XL (recombination of BA.1 and BA.2) and XAL (recombination of BA.1.1 and BA.2), as well as Omicron (BA.4 and sublineages), were classified under “Remaining Lineages” due to their limited representation in the dataset. No available sequencing data were provided for March 2020.

**Figure 2:**
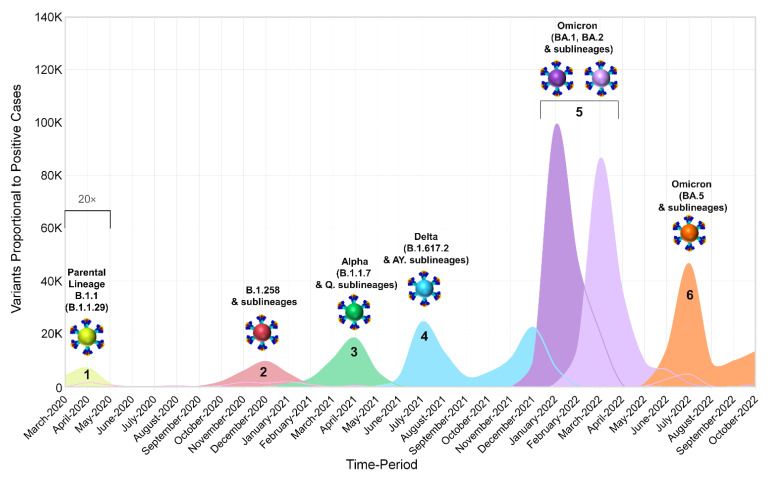
This figure provides an overview of the progression and waves of SARS-CoV-2 in Cyprus, spanning from March 2020 to October 2022. To facilitate continuity and illustrate the evolution of SARS-CoV-2 infections in Cyprus, data from our previous studies conducted between April 2020 and October 2021 [24,25] were integrated. The number of positive SARS-CoV-2 cases reported monthly in Cyprus from March 2020 to October 2022 is depicted. The representation is scaled proportionally to the prevalence of SARS-CoV-2 variants, as indicated in Figure 1A,E,F. The sequences of various lineages, including B.1.1.29 (parental lineage B.1.1), B.1.258 and sublineages, Alpha (B.1.1.7 and Q. sublineages), Delta (B.1.617.2 and AY. sublineages), Omicron 1 (BA.1 and sublineages), Omicron 2 (BA.2 and sublineages), Omicron 5 (BA.5 and sublineages), and remaining lineages, are represented in a smoothed line chart. These lineages are differentiated by colors: bright green, red, green, light blue, purple, lilac, orange, and pink, respectively. The values beneath the black bracket, denoting the period from March 2020 to May 2020, have been multiplied by 20 to enhance their visibility.

The focus of the bioinformatic analyses of this study was Omicron 1, 2, and 5 due to their high prevalence during the fifth and sixth waves of SARS-CoV-2 infection in Cyprus (Figure 1 and Figure 2, Table 1). Specifically, for Omicron 1, the most prevalent lineages during the first part of the fifth wave (November 2021–February 2022) were BA.1.1 (20.23%, 431/2131 sequences) and the parental lineage BA.1 (15.20%, 324/2131). BA.1 (25%, 167/680) and BA.1.1 (27%, 183/680) also dominated during the first peak of the fifth wave (January 2022). Omicron 2 dominated the second part of the fifth wave, February–May 2022, with the parental BA.2 lineage comprising the majority of the lineages identified during that period (53.83%, 1145/2127). During the second peak of the fifth wave (March 2022), the most prevalent lineage was BA.2 (66.79%, 521/780) among all identified lineages. Regarding Omicron 5, which was the most common lineage during the sixth wave (late May 2022–August 2022), the most prevalent sublineages were BA.5.1 (26.47%, 203/767) and BA.5.2 (19.95%, 153/767). During the peak of the sixth wave (July 2022), the most prevalent lineages were BA.5.1 (28.92%, 96/332) and BA.5.2 (20.78%, 69/332). Thus, the bioinformatic analyses focused on Omicron 1 (BA.1 and BA.1.1), Omicron 2 (BA.2), and Omicron 5 (BA.5.1 and BA.5.2). Following the identification of the most prevalent lineages for each wave, a dataset reduction was performed to alleviate the computational burden, in which each dataset (Omicron 1, 2, and 5) contained 150 representative sequences [28]. This dataset reduction entailed calculating the monthly distribution of the aforementioned lineages and randomly selecting 150 sequences according to this distribution using R Studio, version 4.2.0, 2021.09.2+382 “Ghost Orchid” Release [28].

The analysis was conducted separately for each lineage, employing the same delineation strategy as previously documented in our studies [24,25]. Specifically, all newly generated near-complete genomes underwent multiple alignments using MAFFT v.7.475 [29]. Subsequently, the alignments were subjected to visual inspection and manual editing using AliView v.1.26 [30]. A maximum likelihood (ML) tree was then constructed from the refined alignment data utilizing IQtree v.2.1.2 software [31]. To ascertain the robustness of the branches in the constructed tree, we employed the SH-like approximate likelihood ratio test (SH-aLRT) [32] and the ultrafast bootstrap (UFB) procedure [33]. Lineages of interest were identified based on SH-aLRT and UFB thresholds of 90 and 100, respectively.

As in our previous work [24], the size of the datasets precluded integrated analysis to jointly infer epidemic relationships and migration history. For this reason, the same strategy was followed for time-scaled phylogenetic analyses as before [24]. Briefly, this entailed estimating a dated ML tree [31,34], which served as the basis for 1000 rounds of randomly resolving polytomies. The resulting collection of bifurcating trees was then used as an empirical tree distribution for inferring phylogeographic history [35,36,37].

### 2.5. Calculations and Figure Information

Data for Figure 1A,B and Figure 2, were sourced from the Cyprus Ministry of Health, the Press and Information Office, and the KIOS Research and Innovation Center of Excellence (KIOS CoE), operating within the University of Cyprus. Subsequently, these data underwent processing to calculate the percentage positivity, as depicted in Figure 1C [38,39,40]. More specifically, the computations for Figure 1C involved dividing the weekly count of positive SARS-CoV-2 cases by the total number of SARS-CoV-2 tests conducted in the respective week, resulting in the percentage positivity [40].

In the case of Figure 2, the monthly tally of positive SARS-CoV-2 cases reported in Cyprus from March 2020 to October 2022 was proportionally associated with the prevalent SARS-CoV-2 variants. Additionally, for Figure 2, the graphical representations of spike proteins on the colored virions beneath the names of each lineage, as also depicted in the figures below, were generated using PyMol (Version 2.4.1, Schrödinger, LLC, https://www.pymol.org, accessed on 18 February 2021). These representations are based on data derived and adapted from the Protein Data Bank entry 6XEY [41,42], in conjunction with other sources utilized to delineate spike protein domains [43,44,45,46,47,48,49,50,51].

## 3. Results

### 3.1. The Appearance of Lineages and SARS-CoV-2 Waves in Cyprus

In this study, 4700 SARS-CoV-2 whole-genome sequences obtained in Cyprus were analyzed for the period from October 2021 to October 2022. The lineage classification analysis revealed a myriad of lineages, with 167 different lineages identified (Table 1). For the first three-month period (October 2021–December 2021) (Table 1), the most prevalent lineages were Delta (B.1.617.2 and AY. sublineages), accounting for a total of 687/857 sequences (80.2%). Specifically, the most prevalent Delta lineages were AY.43 (22.4%, 192/857), AY.122 (12.37%, 106/857), AY4 (12.02%, 103/857), and the parental lineage B.1.617.2 (5.02%, 43/857) (Table 1). During this three-month period, in December 2021, Omicron sequences were first identified in Cyprus (Figure 1E,F). Specifically, Omicron 1 sequences (BA.1 and sublineages) accounted for 19.71% (169/857), while only one (0.12%, 1/857) Omicron 2 (BA.2 and sublineages) sequence was identified during this period (Table 1). The October 2021–December 2021 period also marked the beginning of the fifth wave in Cyprus, which started with a gradual rise in new infections in November 2021, leading to a large increase in new infections by the end of December 2021 (Figure 1A). This gradual rise in SARS-CoV-2 infections in November 2021 was underlined by Delta variant sequences; however, in December 2021, Omicron variants were rapidly rising, accounting for more than 30% of the identified lineages (31.25%, 170/544) (Figure 1A,E,F and Figure 2). Of note, similar to previous waves of SARS-CoV-2 infections in Cyprus, this initial period of the fifth wave was characterized by a substantially increasing rate of positivity (Figure 1A–C) [24,25].

SARS-CoV-2 cases continued to increase over the next three-month period (January 2022–March 2022), forming the first peak of the fifth wave in January 2022 (Figure 1A and Figure 2). This period marked an increase in the prevalence of the Omicron variant (Table 1, Figure 1E,F and Figure 2), with Omicron 1 being the most common variant in January 2022 (Figure 1E,F and Figure 2), accounting for 91.76% (624/680). Specifically, the most prevalent lineages were BA.1.1 (26.91%, 183/680) and BA.1 (24.56%, 167/680). This prevalence of the Omicron 1 lineage continued over February 2022 (75.71%, 508/671), although there was a minor drop in SARS-CoV-2 cases (Figure 1A), with BA.1.1 (33.53%, 225/671) and BA.1 (14.16%, 95/671) being the most prevalent. However, Omicron 2 lineages began increasing, with BA.2 as the second most prevalent lineage in February 2022 (19.67%, 132/671). The last month of this period, March 2022, showed an increase in SARS-CoV-2 cases again, forming the second peak of the fifth wave. March 2022 also reflected a shift in prevalence, with Omicron 2 becoming the dominant variant (80.77%, 630/780). Specifically, the most prevalent lineage during this month was BA.2, which accounted for the vast majority of identified lineages (66.79%, 521/780). Importantly, the second most prevalent lineage, BA.1.1, only constituted 10.77% (84/780) of the lineages identified during this month, highlighting the dominance of BA.2 (Omicron 2).

The dominance of Omicron 2 (BA.2) even continued for the next three-month period (April 2022–June 2022), with 73.28% (709/967) and 53.98% (522/967) of the total sequences, respectively (Table 1). However, during this period, the number of SARS-CoV-2 cases began dropping until May 2022, signifying the end of the fifth wave (Figure 1A and Figure 2), with Omicron 2 still being identified as the most common variant until May 2022 (81.77%, 148/181) (Table 1, Figure 1E,F and Figure 2). Nonetheless, May 2022 marked the first identification of both the Omicron 4 (2.21%, 4/181) and Omicron 5 (11.61%, 21/181) variants in Cyprus, and by the end of this three-month period, when SARS-CoV-2 cases started to rise again, the landscape of circulating variants had changed greatly (Table 1, Figure 1A,E,F and Figure 2). Specifically, in June 2022, no Omicron 1 sequences were identified, Omicron 2 was no longer dominant (27.49%, 80/291), Omicron 4 (11.68%, 34/291) had a low prevalence, and Omicron 5 (60.14%, 175/291) became the most prevalent variant, signifying the beginning of the sixth wave (Table 1, Figure 1A,E,F and Figure 2). However, unlike Omicron 2, which was dominated by a single lineage (BA.2), several Omicron 5 lineages were identified during this three-month period (April 2022–June 2022), with the two most prevalent Omicron 5 lineages being BA.5.1 (44.9%, 88/196) and BA.5.2 (20.41%, 40/196) (Table 1).

The final period (July 2022–October 2022) encompassed the majority of the sixth wave, with SARS-CoV-2 cases increasing and forming a peak in July 2022, declining by August 2022, and signifying the end of the sixth wave in Cyprus by September 2022 (Figure 1A and Figure 2). During this period, there was also an increase in percent positivity (Figure 1B,C). The sixth wave was almost entirely dominated by Omicron 5, and the most prevalent lineages of Omicron 5 in July 2022, when the peak formed, were BA.5.1 (32.76%, 96/293) and BA.5.2 (23.55%, 69/293). The prevalence of Omicron 5, however, was not just limited to the sixth wave; it expanded throughout the whole July 2022–October 2022 period, constituting 91.99% (686/745) of the total sequences (Table 1, Figure 1E,F and Figure 2). Additionally, the most prevalent Omicron 5 lineages of the whole July 2022–October 2022 period remained BA.5.1 (24.78%, 170/686) and BA.5.2 (26.68%, 183/686). Conversely, Omicron 2, which was the most prevalent variant of the previous period (April 2022–June 2022), only constituted 2.55% (19/745) of the sequences of this period, while Omicron 4 remained relatively low in prevalence at 5.36% (40/745) (Table 1, Figure 1E,F and Figure 2).

In summary, by the end of the sampling period, the fifth and sixth waves of infections had been identified, each characterized by particular dominant groups of lineages. These lineages were (in order of chronological appearance) Omicron 1 (BA.1 and BA.1.1), Omicron 2 (BA.2), and Omicron 5 (BA.5.1 and BA.5.2).

### 3.2. SARS-CoV-2 Spike Protein Mutations

**Figure 3:**
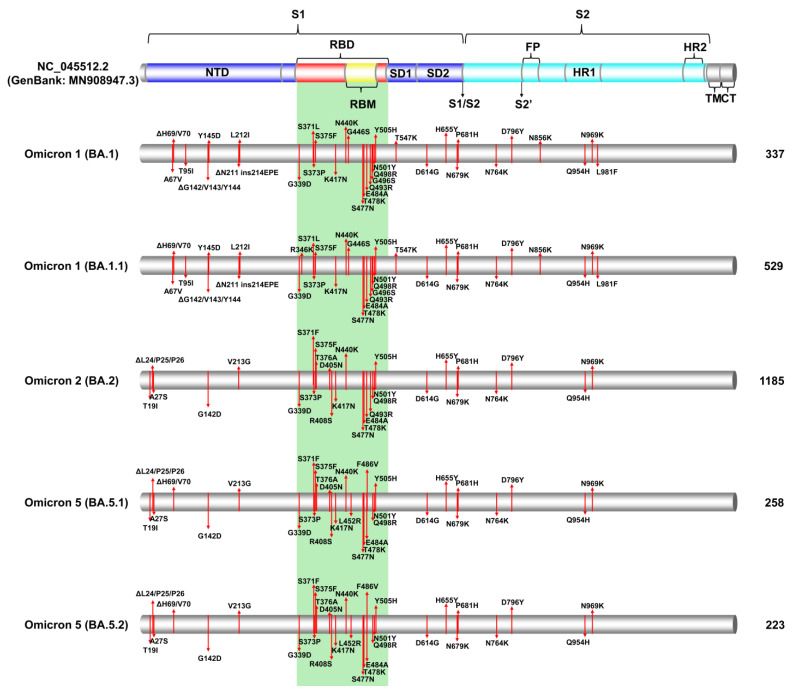
The figure effectively illustrates the significant S protein mutations within these prominent SARS-CoV-2 lineages during the specified period, shedding light on the molecular characteristics of the virus during the 5th and 6th waves in Cyprus. These mutations were identified through the analysis of Cypriot SARS-CoV-2 sequences collected between October 2021 and October 2022. Specifically, the figure focuses on the most common S protein mutations found in Omicron 1 (BA.1, BA.1.1), Omicron 2 (BA.2), and Omicron 5 (BA.5.1, BA.5.2) lineages. A colored cylinder represents the essential domains of the SARS-CoV-2 S protein (GenBank: MN908947.3), including the N-terminal domain (NTD); the receptor-binding domain (RBD), highlighted in red; the receptor-binding motif (RBM) in yellow; subdomains 1 and 2 (SD1 and SD2); fusion peptide (FP); subunit 1 (S1) in blue; subunit 2 (S2) in cyan; heptad repeats (HRs); the transmembrane domain (TM) in gray; and the cytoplasmic tail (CT) in gray. Cleavage sites (S1/S2 and S2′) are marked with black arrows. The green-highlighted area corresponds to the receptor-binding domain (RBD) [42,43,44,45,46,47,48,49,50,51]. Mutations are indicated by red lines, denoting the locations of the most common mutations identified in all or nearly all sequences within a given lineage. The figure also provides information on the total number of sequences analyzed for each lineage in this study, displayed on the right side of the figure.

Spike protein mutations were identified as the most prevalent lineages during the fifth and sixth waves of SARS-CoV-2 infections in Cyprus (Figure 3, Tables S1 and S2). Namely, these were Omicron 1 (BA.1, BA.1.1) with Omicron 2 (BA.2) during the fifth wave and Omicron 5 (BA.5.1, BA.5.2) during the sixth wave (Figure 1 and Figure 2 and Table 1). This section focuses on the spike protein due to its importance in the transmissibility and lifecycle of the virus, coupled with its prominence as a vaccine and diagnostic target [52,53].

#### 3.2.1. Common Spike Protein Mutations: The Most Prevalent Lineages/Variants in Cyprus

The number of mutations identified in the most prevalent lineages during the fifth and sixth waves highlighted the towering difference from those of previous waves [24] (Figure 3, Tables S1 and S2). Since the most prevalent lineages of the fifth and sixth waves belonged to the Omicron VOC, they had more than 30 mutations in the spike protein, compared to previous prevalent VOCs such as Alpha and Delta, which had approximately 10 mutations [13,14]. The Delta (B.1.617.2) variant and its AY. sublineages, which were the most common lineages during the previous wave (fourth wave), were still identified until February 2022, while Omicron was dominant (Table 1, Figure 1 and Figure 2). The most common Delta (B.1.617.2) mutations were T19R, G142D, ΔE156/F157, R158G, L452R, T478K, D614G, P681R, and D950N (Table S1). However, there was a plethora of Delta AY. sublineages that were identified (45 AY. lineages), and not all contained the above set of mutations, such as AY.4.2, which additionally encompassed Y145H and A222V (Table S1). However, despite this diversity within the Delta variant, the Omicron VOC was far more diverse, with 121 total lineages (Table 1).

After Delta, Omicron 1 reached high prevalence and was dominant during the first peak of the fifth wave, with the BA.1 and BA.1.1 lineages. The most common BA.1 spike mutations were A67V, ΔH69/V70, T95I, ΔG142/V143/Y144, Y145D, ΔN211, L212I, ins214EPE, G339D, S371L, S373P, S375F, K417N, N440K, G446S, S477N, T478K, E484A, Q493R, G496S, Q498R, N501Y, Y505H, T547K, D614G, H655Y, N679K, P681H, N764K, D796Y, N856K, Q954H, N969K, and L981F (Figure 3). Notably, the three-amino acid deletion and substitution ΔG142/V143/Y144, Y145D, is also reported as G142D, ΔV143/Y144/Y145 [54]. The other highly prevalent Omicron 1 lineage, BA.1.1, a sublineage of the parental BA.1, encompassed the R346K mutation in addition to the aforementioned mutations (Figure 3, Table S1). Furthermore, there were 18 other lower-prevalence Omicron 1 sublineages found in this dataset that also hosted mutations not present in the parental BA.1. These were BA.1.1.1, BA.1.1.11, BA.1.1.14, BA.1.1.15, and BA.1.1.18, which also contained R346K, similar to BA.1.1, BA.1.8 with P809S, and BA.1.17.2 with A701V (Table S1). Omicron 1 shared mutations found in other VOCs, such as, but not limited to, ΔH69/V70, K417N, T478K, and N501Y, highlighting the ability of the Omicron variant to evolve and acquire high-risk mutations [7,55].

Next, the Omicron 2 VOC was predominant during the second peak of the fifth wave with the BA.2 lineage (Omicron 2 parental lineage) (Table 1, Figure 1 and Figure 2). The most common BA.2 spike mutations were T19I, ΔL24/P25/P26, A27S, G142D, V213G, G339D, S371F, S373P, S375F, T376A, D405N, R408S, K417N, N440K, S477N, T478K, E484A, Q493R, Q498R, N501Y, Y505H, D614G, H655Y, N679K, P681H, N764K, D796Y, Q954H, and N969K (Figure 3). BA.2 was significantly more prevalent than any other Omicron 2 lineage, with 77.86% (1185/1522) of Omicron 2 sequences (Table 1); however, there was also a higher degree of variability among the remaining Omicron 2 lineages (< 30) identified during this study period compared to Omicron 1. Some of the most notable Omicron 2 sublineages identified in this study, reported to be of high risk, were BA.2.3.20 with the mutations K444R, N450D, L452M, N460K, E484R, and Q493R reversal; BA.2.12.1 with L452Q and S704L; and BN.1.3, a sublineage of BA.2.75, with K147E, W152R, F157L, I210V, G257S, G339H, R346T, K356T, G446S, N460K, F490S, and Q493R reversal [5,56,57,58,59]. Moreover, XBB.1 and XBB.2 were identified, which are sublineages of the XBB (Omicron 2) recombinant encompassing the mutations V83A, ΔY144, H146Q, Q183E, V213E, G339H, R346T, L368I, V445P, G446S, N460K, F486S, F490S, and Q493R reversal. XBB.1 additionally contained G252V, while XBB.2 had D253G (Table S1) [5,56,57,58,59].

Following Omicron 2, Omicron 5 became the most prevalent variant during the sixth wave, with the predominant lineages being BA.5.1 and BA.5.2 (Table 1, Figure 1 and Figure 2). Both BA.5.1 and BA.5.2 shared the same spike mutations: T19I, ΔL24/P25/P26, A27S, ΔH69/V70, G142D, V213G, G339D, S371F, S373P, S375F, T376A, D405N, R408S, K417N, N440K, L452R, S477N, T478K, E484A, F486V, Q498R, N501Y, Y505H, D614G, H655Y, N679K, P681H, N764K, D796Y, Q954H, and N969K (Figure 3). BA.5.2 was defined by the D16G mutation in ORF9b [60,61]. Nonetheless, Omicron 5 was identified as the most diverse of the three Omicron subvariants, with 59 lineages identified in this study (Table 1). Notably, among the low-prevalence Omicron 5 sublineages were the “BF”. group of lineages, with BF.7 reported to have led to large spikes of SARS-CoV-2 infections [62]. Members of “BF.” primarily encompassed the R346T mutation, while ΔY144, which was common for the Alpha variant and Omicron 1, was identified in BF.7.5.1 (Table S1) [63]. Additionally, ΔY144 was found in BQ.1.8, which is part of the “BQ.” group of Omicron 5 lineages that gained notoriety with large spikes of infections worldwide [64]. The members of “BQ.” primarily encompassed the K444T and N460K mutations, while the R346T mutation was also found in BQ.1 sublineages, such as BQ.1.1, which even rose to prevalence in many countries briefly [64]. Importantly, during the sixth wave, when Omicron 5 was prevalent, Omicron 4 was also in circulation at a lower prevalence. The parental lineages for Omicron 4 and 5 shared the same spike mutations; however, BA.4.6 also encompassed the R346T mutation found in BF.7 and BQ.1.1 [64].

**Figure 4:**
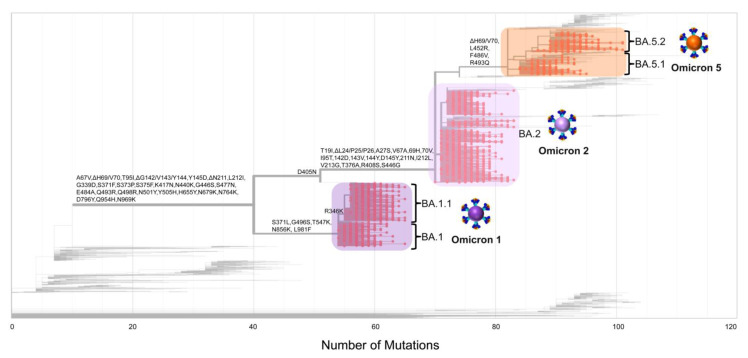
This figure effectively visualizes the evolutionary relationships and mutation patterns within these prominent SARS-CoV-2 lineages during the specified time frame, providing valuable insights into the viral dynamics during the 5th and 6th waves in Cyprus from October 2021 to October 2022. The maximum likelihood tree portrays the evolution of mutations and was generated using Nextclade (https://clades.nextstrain.org, last accessed on 31 May 2023 [27]). The analysis includes SARS-CoV-2 sequences classified as Omicron 1 (BA.1, BA.1.1), Omicron 2 (BA.2), and Omicron 5 (BA.5.1, BA.5.2). The maximum likelihood tree represents the evolutionary relationships between these lineages. Mutations that originated earlier during the pandemic are typically positioned on the left-hand side of the figure, while those that emerged or reappeared as SARS-CoV-2 evolved into new lineages are situated on the right-hand side. Highlighted in purple, lilac, and orange rectangles are the sequences of the most prevalent lineages from the 5th wave (Omicron 1-BA.1, BA.1.1, and Omicron 2-BA.2) and the 6th wave (Omicron 5-BA.5.1, BA.5.2) in Cyprus, respectively. Red dots on the tree symbolize the sequences belonging to Omicron 1 (BA.1, BA.1.1), Omicron 2 (BA.2), and Omicron 5 (BA.5.1, BA.5.2) used in this study.

As the pandemic progressed, increasingly diverse SARS-CoV-2 lineages were identified, each with varying sets of mutations. Additionally, many lineages evolved to include mutations that were also present in previous variants associated with substantial surges in new infections [14,65]. This phenomenon is evident in Figure 4, where ΔH69/V70 deletions are present in both Omicron 1 and Omicron 5. Notably, despite the closer genetic relationship between Omicron 2 and Omicron 5, this deletion was observed in Omicron 5 and Omicron 1 [14,65]. Figure 3 and Figure 4 show that despite the increased number of mutations in the spike protein, the majority of them were situated within the S1 subunit, specifically in the receptor-binding domain (RBD) and N-terminal domain (NTD), as with previous variants. These regions were also reported to have increased selection pressures exerted upon them relative to other spike regions [66]. Thus, during the study period, Cyprus experienced the passage of Delta and the emergence of Omicron. After that, different Omicron subvariants cycled into prevalence, each with their own set of mutations, with the final one being Omicron 5 in this study. However, by the end of this study, Omicron 5 sublineages, such as BQ.1.1, were circulating, along with Omicron 2 sublineages, such as BN.1.3, and even recombinants of Omicron 2like XBB sublineages, all exhibiting recurrent high-risk mutations that included R346, K444, L452, N460, and F486 (Figure 3 and Figure 4, Table S1) [67].

#### 3.2.2. Identification of Omicron-like Spike Protein Mutations in Delta Variants, Nicknamed Deltacron Variants: In-Depth Molecular Characterization

The examination of our dataset unveiled Delta sequences harboring atypical mutations reminiscent of those frequently observed in Omicron 1, a variant that briefly cocirculated alongside Delta. More precisely, these sequences were ascertained in the nascent phase of the fifth wave, spanning from December 2021 to January 2022 (as depicted in Figure 2). To facilitate comprehension among the general public, these sequences were designated “Deltacron”. This nomenclature choice aimed to simplify discourse and enable subsequent investigations into these unique genomic entities. The Deltacron sequences emerged through the analysis of weekly SARS-CoV-2 reports conducted within our laboratory. These reports are integral to our comprehensive efforts encompassing epidemiological, genetic, and phylogenetic inquiries pertaining to SARS-CoV-2 infections within the Cyprus region. This initiative was undertaken collaboratively with the Ministry of Health. Specifically, these sequences were derived from specimens obtained from both hospitalized and nonhospitalized individuals with SARS-CoV-2 infections in Cyprus (see Section 2, Materials and Methods, for further details). The Deltacron sequences had a Delta backbone but encompassed mutations that included A67V, T95I, ΔH69/V70, ΔG142/V143/Y144, and Y145D (Table S2), which were also common for the then cocirculating Omicron 1. Notably, mutations such as T95I and G142 were also found in Delta lineages (Figure 3, Table S1), and as mentioned previously, ΔH69/V70 recurrently emerged in various lineages, including B.1.1.7 (Alpha VOC) [14,65], highlighting the possibility of this re-emergence occurring in this instance as well [14]. Moreover, these mutations were identified in regions such as the NTD of the SARS-CoV-2 spike protein, which are known antigenic supersites and mutational hotspots, thus conferring a logical basis for the natural acquisition of the aforementioned mutations [68,69].

It is imperative to underscore that the processes of RNA extraction and polymerase chain reaction (PCR) amplification were executed by distinct laboratory entities. Notably, the identification of Deltacron sequences occurred across diverse NGS runs within the purview of the contracted organization, Medicover Genetics. Moreover, during the period of Deltacron identification, analogous sequences were submitted to and acknowledged in online repositories maintained by reputable research institutions worldwide. These esteemed laboratories, located in diverse regions, including Israel, India, Thailand, France, and Italy, collectively contributed to the accumulation of such sequences. Examples of Global Initiative on Sharing All Influenza Data (GISAID) identifiers associated with these sequences, although not exhaustively listed, comprise EPI_ISL_8296059, EPI_ISL_8329009, EPI_ISL_8374888, EPI_ISL_8355657, and EPI_ISL_8375468) [70].

However, to meticulously explore the potential origins of these findings, including whether they stemmed from contamination or coinfection scenarios or were indeed representative of the Deltacron phenomenon, a comprehensive sequencing analysis was conducted. This analysis aimed to dissect instances in which a particular specimen might harbor a Delta variant concurrently with Omicron 1, as illustrated in Figure S1A–C. The outcomes of these analyses revealed that a scenario involving contamination or coinfection would likely give rise to a frameshift mutation occurring at positions 21,987 to 21,990 (specifically, GTGT, with numbering referencing GenBank entry MN908947.3). This investigation delved into the molecular intricacies, with particular emphasis on the sequencing analysis focused on the amplification of SARS-CoV-2 genomes. To facilitate this analysis, the ARTIC V3 primer set [71,72] was employed as part of the sequencing protocol. The primer set employed, previously recognized for encountering challenges in effectively interacting with the Delta variant, has been linked to suboptimal binding of the ARTIC V3 right primer 72 [73]. Of particular importance, as per our current analyses, it has been determined that the definitive binding position for this primer occurs within the genomic coordinates of 22,014 to 22,038. This puts the location of the characteristic six-nucleotide Delta deletion (AGTTCA at positions 22,029–22,034, resulting in ΔE156/F157, R158G) within the binding site of the ARTIC V3 right 72 primer (Figure S1A,B). However, a largely underrepresented aspect is the analogous primer binding concern encountered with the ARTIC V3 primer set in relation to Omicron 1 variants. In this context, the left primer within ARTIC V3 primer pair 73 (spanning positions 21,962 to 21,990) manifested suboptimal binding characteristics with Omicron 1 variants. This was primarily attributed to a distinctive nine-nucleotide deletion (GTGTTTATT) positioned at coordinates 21,987 to 21,995. This genetic alteration led to consequential amino acid deletions and substitutions, specifically ΔG142/V143/Y144, and Y145D, as delineated in Figure S1A,B. This deletion distinctly impacted four nucleotides (GTGT) located at the 3′ end of the left ARTIC V3 73 primer, as illustrated in Figure S1B. Consequently, in scenarios where Omicron 1 was coexisting within Delta samples, the amplification process would lead to the preferential amplification of Omicron 1 by the right 72 primer, while the left 73 primer would tend to amplify Delta sequences. Thus, when dealing with a sample containing both Delta and Omicron 1 variants, the sequencing outcome would exhibit the absence of solely the initial four nucleotides (GTGT) from the broader nine-nucleotide Omicron 1 deletion (GTGTTTATT), situated within positions 21,987 to 21,990. This disparity would ultimately result in a frameshift mutation. This distinctive frameshift is notably evident in Figure S1C by the alignment of sequencing fragments from an Omicron 1 sample, where the right ARTIC V3 primer pair 72 amplified Omicron 1, and the alignment of the sequencing fragments from a Delta sample, where the ARTIC V3 primer pair 73 amplified Delta, as shown in Figure S1.

None of the Deltacron sequences that were shared through our submissions to the GISAID database or those received from the aforementioned international sources displayed the previously stated frameshift characteristic. It is worth noting, however, that we pinpointed alternative sequences within our dataset that exhibited the frameshift trait indicative of potential contamination or coinfection. This particular phenomenon, while relatively uncommon, was observed in a limited subset (0.08%, 5 out of 5677) of cases, excluding instances of missing data and duplicate entries. Consequently, these instances were deemed unsuitable for submission and were therefore omitted, as demonstrated in Figure S2 [8,74]. Precisely, these particular sequences distinctly manifested the frameshift alteration as previously outlined, which is anticipated when a sample contains both the Delta and Omicron 1 variants. In these Delta sequences featuring the frameshift phenomenon, the sequencing fragments derived from the ARTIC V3 right primer 72 exhibit the genomic characteristics associated with Omicron 1. This was discernible through the presence of the distinctive nine-nucleotide Omicron 1 deletion (GTGTTTATT) at coordinates 21,987 to 21,995. Conversely, the sequencing fragments obtained from the ARTIC V3 left primer 73 distinctly showcased the Delta variant, as indicated by the observable six-nucleotide deletion (AGTTCA) representing Delta/AY, spanning positions 22,029 to 22,034. Furthermore, notably, the nucleotide sequence amplified by ARTIC V3 primer 73 commences with TTATC (as indicated in Figure S2), occupying positions 21,991 to 21,995. If the ARTIC V3 primer 73 had indeed facilitated the amplification of Omicron 1, the nucleotides TTATC at positions 21,991 to 21,995 would have been subject to deletion (as depicted in Figure S2). Consequently, in the scenario where the right ARTIC V3 primer 73 amplified the Delta variant and the left ARTIC V3 primer 72 amplified Omicron 1, the result would be the emergence of a four-nucleotide deletion positioned at 21,987 to 21,990. This specific deletion instigated the aforementioned frameshift event, as elegantly illustrated in Figure S2.

This frameshift event was similarly identified within an Omicron 1 sequence present in our dataset. This observation underscores the possibility of contamination or coinfection, consequently rendering the sequence ineligible for deposition. As previously highlighted, the left ARTIC V3 73 primer exhibited suboptimal binding characteristics with Omicron 1. Thus, within an Omicron 1 sample concurrently harboring the Delta variant, the binding affinity of the left ARTIC V3 73 primer to Omicron 1 would indeed be compromised due to the nine-nucleotide deletion (GTGTTTATT) at positions 21,987 to 21,995 (resulting in ΔG142/V143/Y144, and Y145D mutations). As a result, the manifestation of the aforementioned frameshift was anticipated. This premise gains further support through the identification of a sequence that aligns with these expectations, as exemplified in Figure S3, thereby reinforcing the validity of the frameshift hypothesis.

An added rationale bolstering the credibility of the identification of Delta genomes displaying Omicron 1 signatures stems from the observation of Delta sequences within this dataset that were devoid of the hallmark Delta deletion at positions 22,029 to 22,034 (resulting in ΔE156/F157 and R158G mutations), as documented in Table S1. Specifically, since this deletion was not present, it would not constitute a hindrance to the right ARTIC V3 primer 72. However, deletions such as A67V, ΔH69/V70, T95I, ΔG142/V143/Y144, and Y145D were still identified in those Delta sequences that lacked the characteristic Delta deletion 22,029–22,034 (ΔE156/F157, R158G) (Table S1). Furthermore, the presence of the Omicron 1 deletion 21,987–21,995 (ΔG142/V143/Y144, Y145D) in these sequences served as a positive indication that primer 73 would have amplified Delta, due to the suboptimal binding of the left 73 primer with Omicron 1 genomes. However, deletion 22,029–22,034 (ΔE156/F157, R158G) was not present in all Delta sequences of this dataset (Table S1). Indeed, it is noteworthy that on a global scale, the distinctive Delta deletion at positions 22,029 to 22,034 was not universally present across all submitted Delta sequences. Notably, it was reported to be detected in approximately 98.7% of the B.1.617.2 and AY sublineages [60]. Nevertheless, notwithstanding the comprehensive array of arguments presented thus far, it remains imperative to conduct a holistic consideration of all conceivable scenarios.

By elucidating the aforementioned justifications, it was effectively demonstrated that none of the Deltacron sequences submitted exhibited the anticipated frameshift arising from a four-nucleotide deletion at 21,987 to 21,990. This is a characteristic expected to emerge in the sequencing output of a Delta sample potentially tainted by Omicron 1 contamination or coinfection. The investigation underscored that this frameshift occurrence directly resulted from the distinct deletions inherent to the specific variants, found within the binding domains of the ARTIC V3 right 72 and left 73 primers.

Specifically, the presence of the characteristic Delta deletion at positions 22,029 to 22,034 contributed to the suboptimal binding of the former primer, while a similar suboptimal binding for the latter primer was instigated by the 21,987 to 21,995 deletion associated with Omicron 1. This interplay led to the ARTIC V3 right 72 primer displaying a propensity for amplifying the Omicron 1 variant, while the ARTIC V3 left 73 primer favored the amplification of Delta sequences. This divergent amplification behavior culminated in the aforementioned frameshift event. Consequently, driven by the amalgamation of these rationales and considering the virus’s inherent biological and evolutionary potential for such mutations and recombination events, the prompt dissemination of our findings prior to the publication of the peer-reviewed manuscript was deemed essential.

### 3.3. Phylogeny of Cypriot SARS-CoV-2 Sequences

**Figure 5:**
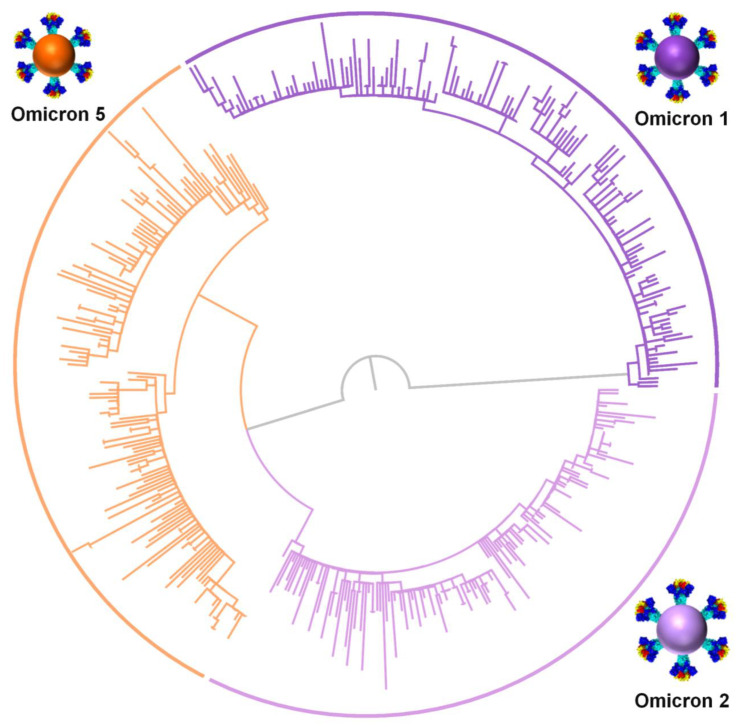
Maximum likelihood phylogenic tree of the 450 representative Cypriot SARS-CoV-2 genomes classified as Omicron 1 (BA.1, BA.1.1), 2 (BA.2), and 5 (BA.5.1, BA.5.2). The sequences of the most prevalent lineages of the fifth wave Omicron 1 (BA.1, BA.1.1) and 2 (BA.2), as well as the sixth wave Omicron 5 (BA.5.1, BA.5.2), are highlighted in purple, lilac, and orange, respectively.

The phylogeny of Omicron 1 (BA.1, BA.1.1), 2 (BA.2), and 5 (BA.5.1, BA.5.2) confirmed the genetic relationships among the three variants that comprised the fifth and sixth waves of SARS-CoV-2 infection in Cyprus. As shown in Figure 5, Omicron 1 is more genetically distant than both Omicron 2 and 5, which is consistent with the previous findings of this study (Figure 4). This analysis serves as a clear indication of the variant subset delineation employed for downstream time-scaled migration histories.

### 3.4. Time-Scaled Migration Histories

**Figure 6:**
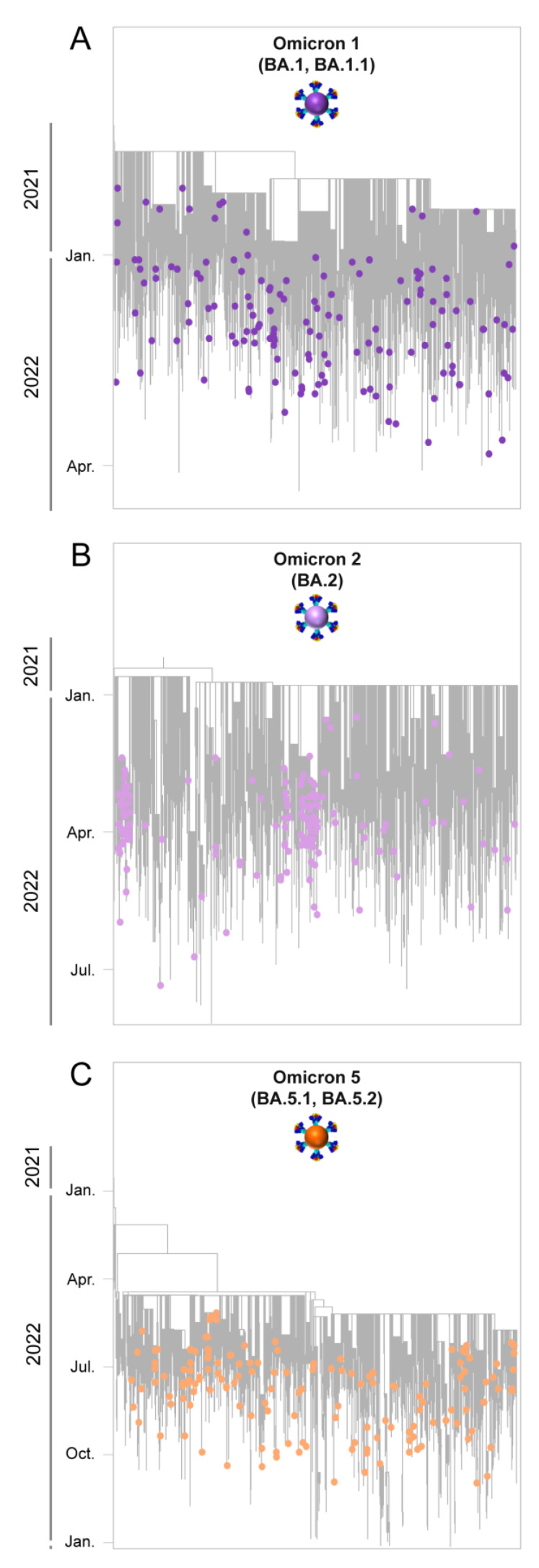
Time-scaled migration history for the datasets used for reconstructing the history of the spread of (**A**) Omicron 1 (BA.1 and BA.1.1, purple circles), (**B**) Omicron 2 (BA.2, lilac circles), and (**C**) Omicron 5 (BA.5.1 and BA.5.2, orange circles). Gray tips represent reference sequences downloaded from GISAID (accessed on 12 February 2023) [70].

**Figure 7:**
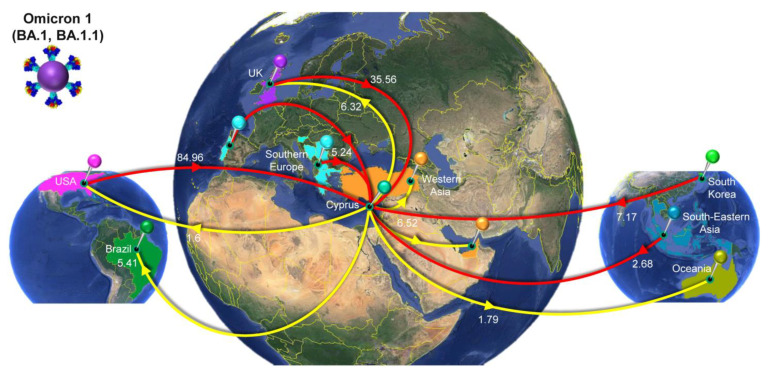
Map of SARS-CoV-2 Omicron 1 (BA.1, BA.1.1) transmission between Cyprus and other countries. The geographic origins of SARS-CoV-2 Omicron 1 (BA.1, BA.1.1) imported into Cyprus are depicted with red lines; exports from Cyprus to other countries are depicted with yellow lines. Countries acting as “sources” or “sinks” for SARS-CoV-2 Omicron 1 (BA.1, BA.1.1) transmission are highlighted and labeled, and the estimated average number of migration events is indicated. In southern Europe, the highlighted countries are Bosnia and Herzegovina, North Macedonia, Serbia, Portugal, Slovenia, and Greece. In Western Asia, the highlighted countries are Turkey and the United Arab Emirates. In South-Eastern Asia, the highlighted countries are Indonesia, Singapore, Thailand, the Philippines, and Malaysia. In Oceania, the highlighted countries are Australia, the Northern Mariana Islands, and Guam. To enhance the clarity of the figure, only the locations with the five highest estimated total average numbers of importation or exportation events are displayed (Table 2). Map images courtesy of Google Earth Pro 7.3.2.5776 and 7.3.4.8642 (14 December 2015). Global view centered on North and South America (left), 5°49′53.21″ N 81°12′52.44″ W, Eye alt 9503.85 km. Europe (middle), 36°16′38.78″ N 36°07′29.71″ E, Eye alt 7949.12 km. South-Eastern Asia and Oceania (right), 1°14′19.88″ N 112°15′56.16″ E, Eye alt 11,201.60 km. US Dept. of State Geographer, DATA SIO, NOAA, U.S. Navy, NGA, and GEBCO. Image Landsat/Copernicus. 2018 and 2023 © Google. https://www.google.com/earth/versions/#earth-pro (accessed on 10 April 2019 and 16 July 2023).

**Figure 8:**
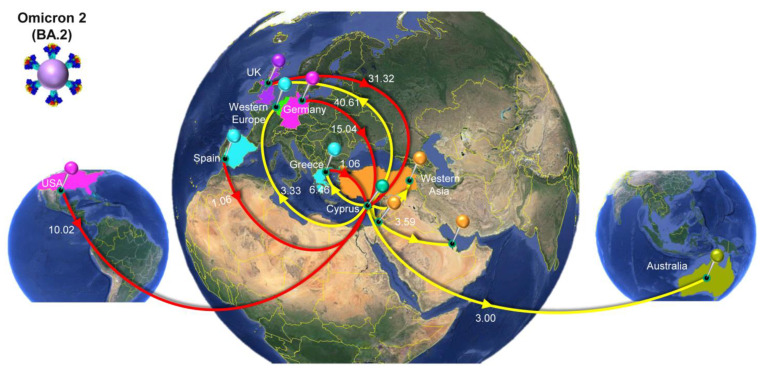
Map of SARS-CoV-2 Omicron 2 (BA.2) transmission between Cyprus and other countries. The geographic origins of SARS-CoV-2 Omicron 2 (BA.2) imported into Cyprus are depicted with red lines; exports from Cyprus to other countries are depicted with yellow lines. Countries acting as “sources” or “sinks” for SARS-CoV-2 Omicron 2 (BA.2) transmission are highlighted and labeled, and the estimated average number of migration events is indicated. In Western Europe, the highlighted countries are Luxembourg, Belgium, and the Netherlands. In Western Asia, the highlighted countries are Bahrain, Turkey, and Israel. To enhance the clarity of the figure, only the locations with the five highest estimated total average numbers of importation or exportation events are displayed (Table 2). Map images courtesy of Google Earth Pro 7.3.2.5776 and 7.3.4.8642 (14 December 2015). Global view centered on North and South America (left), 5°49′53.21″ N 81°12′52.44″ W, Eye alt 9503.85 km. Europe (middle), 36°16′38.78″ N 36°07′29.71″ E, Eye alt 7949.12 km. South-Eastern Asia and Oceania (right), 1°14′19.88″ N 112°15′56.16″ E, Eye alt 11,201.60 km. US Dept. of State Geographer, DATA SIO, NOAA, U.S. Navy, NGA, and GEBCO. Image Landsat/Copernicus. 2018 and 2023 © Google. https://www.google.com/earth/versions/#earth-pro (accessed on 10 April 2019 and 16 July 2023).

**Figure 9:**
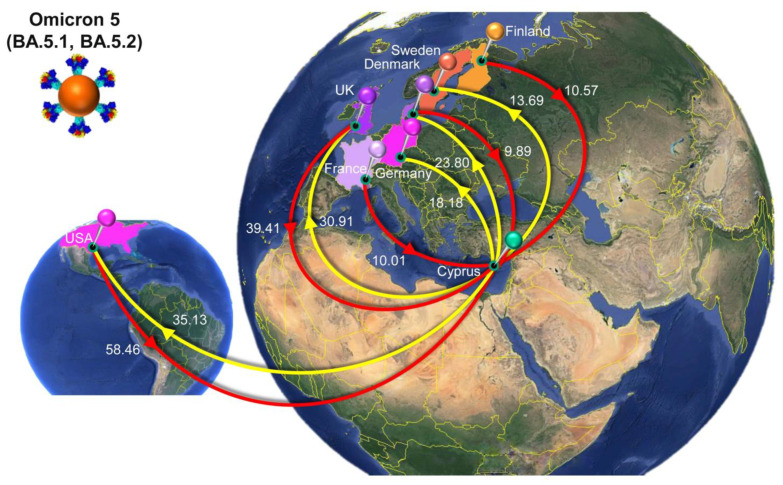
Map of SARS-CoV-2 Omicron 5 (BA.5.1, BA.5.2) transmission between Cyprus and other countries. The geographic origins of SARS-CoV-2 Omicron 5 (BA.5.1, BA.5.2) imported into Cyprus are depicted with red lines; exports from Cyprus to other countries are depicted with yellow lines. Countries acting as “sources” or “sinks” for SARS-CoV-2 Omicron 5 (BA.5.1, BA.5.2) transmission are highlighted and labeled, and the estimated average number of migration events is indicated. To enhance the clarity of the figure, only the locations with the five highest estimated total average numbers of importation or exportation events are displayed (Table 2). Map images courtesy of Google Earth Pro 7.3.2.5776 and 7.3.4.8642 (14 December 2015). Global view centered on North and South America (left), 5°49′53.21″ N 81°12′52.44″ W, Eye alt 9503.85 km. Europe (right), 36°16′38.78″ N 36°07′29.71″ E, Eye alt 7949.12 km. US Dept. of State Geographer, DATA SIO, NOAA, U.S. Navy, NGA, and GEBCO. Image Landsat/Copernicus. 2018 and 2023 © Google. https://www.google.com/earth/versions/#earth-pro (accessed on 10 April 2019 and 16 July 2023).

The migration histories were reconstructed using representative sequences of the most prevalent lineages during the fifth and sixth waves of SARS-CoV-2 infections in Cyprus (Figure 6, Figure 7, Figure 8 and Figure 9, Table 2). The locations of the highest estimated importation and exportation events for Omicron 1 (BA.1 and BA.1.1), Omicron 2 (BA.2), and Omicron 5 (BA.5.1 and BA.5.2) can be visualized in Figure 7, Figure 8 and Figure 9, respectively, while the overall data are available in Table 2.

**Figure 10:**
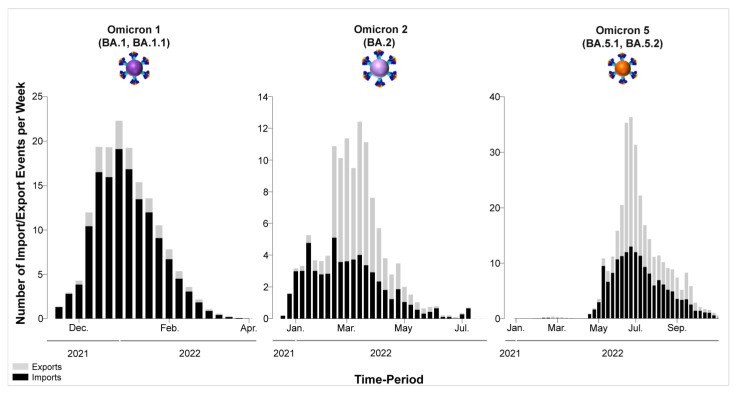
This visual representation effectively conveys the temporal patterns of SARS-CoV-2 import and export activities involving Cyprus, offering insights into the dynamics of viral transmission and movement during the period under consideration. Each column in the graph corresponds to one week, with the width indicating the duration of that week and the height of each column representing the mean estimated total number of migration events (to or from Cyprus) occurring during that specific week. Exports from Cyprus are depicted in gray columns, while imports into Cyprus are shown in black columns. The *y*-axis represents the number of import/export events per week, quantifying the flow of SARS-CoV-2 to and from Cyprus. The *x*-axis represents time, showcasing the progression of weeks over the specified period.

Omicron 1 was the most prevalent variant during the first peak of the fifth wave, with BA.1 and BA.1.1, and the first import event was estimated to be 20 November 2021 (95% highest probability density (HPD): 17 November 2021–24 November 2021) from the USA (0.98 posterior support). Unlike previous periods (second wave, B.1.258 [24,25]), there did not seem to be a large Cypriot clade in line with the scenario of continuous inland transmission (Figure 6A). Instead, the Cypriot Omicron 1 sequences in Figure 6A (left clade BA.1, right clade BA.1.1) were interspersed, indicative of a situation driven by an influx of new Omicron 1 infections. Indeed, the fifth wave, characterized by BA.1 and BA.1.1, largely resulted from the importation of new infections. It began with a low number of cases in late December 2021, peaked in January 2022, and ultimately declined sharply in February 2022. Although imports of Omicron 1 reached as high as almost 20 per week, exports remained comparatively lower and never exceeded half of the maximum number of imports at any point during Omicron 1′s reign (Figure 10). This disparity between the weekly imports and exports can be seen in Table 2, with the total average number of imports (139.06, 100%) being more than six times that of the exports (21.62, 100%). The origins of Omicron 1 in Cyprus were estimated to be the USA with high support, which was in line with the majority of imports also being from the USA and accounting for 84.96 (61.10%) of the total imports, followed by the UK with 35.56 (25.57%) of imports (Figure 7, Table 2). Albeit to a lesser extent, the analyses also revealed South Korea (7.17, 5.16%), Southern Europe (5.24, 3.77%), and South-Eastern Asia (2.68, 1.93%) as the remaining top 5 sources of Omicron 1 in Cyprus. Conversely, the USA (1.6, 7.40%) was the least frequent location to which Cyprus exported Omicron 1, while the UK (6.32, 29.23%) retained a prominent position even as an export location. The remaining location sinks of Omicron 1 were Western Asia (6.52, 30.16%), Brazil (5.41, 25.02%), and Oceania (1.79, 8.28%). Overall, these data showed a plethora of locations across four continents involved in the traffic of Omicron 1 to and from Cyprus, with the USA as the origin and main import source, followed by the UK, which also acted as a prominent export sink, along with Asian, European, and Oceanic locations playing an important role in the transmission of Omicron 1 to and from Cyprus.

The second peak of the fifth wave of SARS-CoV-2 infections in Cyprus, which was primarily composed of Omicron 2 (BA.2) lineages, was estimated to have been imported on 27 December 2021 (95%HPD: 21 December 2021–2 January 2022), from the UK (0.98 posterior support) based on this dataset. Importantly, the first Omicron 2 sample was collected on 15 December 2021, although, due to convergence and rooting issues, this sample was excluded from the time-scaled migration analysis. Upon its exclusion, the estimated earliest date of import for the dataset was 27 December 2021. Thus, while this estimate indicates a potential secondary introduction or spread, the presence of the 15 December 2021 sample suggests that Omicron 2 was already in Cyprus by mid-December. However, unlike the interspersed Omicron 1 sequences shown in Figure 6A, two clades could be discerned for Omicron 2 (Figure 6B). Despite the aggregation of Cypriot Omicron 2 sequences into two clades, it is unlikely that they represented inland transmission clusters, as they were not highly supported and did not comprise the entirety of their respective clades. Specifically, the left clade included 53 sequences, 36 of which were Cypriot, and the right clade encompassed 86 sequences, 50 of which were Cypriot (Figure 6B). Interestingly, Omicron 2 was characterized by lower levels of imports and exports relative to Omicron 1, with imports only reaching approximately five per week (Figure 10). Conversely, exports showed a marked increase during the end of February, reaching almost 10 per week before finally plummeting again by the end of April 2022 (Figure 10). Nonetheless, despite the variance in the distribution of imports and exports in Omicron 2, the final number of migration events was approximately equal for both (Table 2). Specifically, there were 59.95 (100%) total import events and 61.55 (100%) export events, with the UK accounting for the majority of both imports (31.32, 52.24%) and exports (40.61, 65.98%) (Figure 8, Table 2). The second highest location regarding imports was Germany, equating to 15.04 import events (25.09%), which was only half as much as the primary source, the UK (Figure 8, Table 2). The remaining three locations of the top five sources were the USA (15.04, 16.71%), Greece, and Spain (1.06, 1.77%). On the other hand, the second highest sink toward which Omicron 2 was exported was Greece (6.46, 10.50%), with 1/6 of export events coming from the primary sink, the UK. The remaining three of the top five locations of exports were Western Asia (3.59, 5.83%), Western Europe (3.33, 5.41%), and Australia (3, 4.87%). Additionally, the USA was still identified as a sink to which Omicron 2 was exported, albeit to a lesser extent. Therefore, Omicron 1 and Omicron 2 shared similar source and sink locations, primarily the UK and USA, with Europe, Asia, and Oceania also identified as source/sink locations for both of these datasets.

The sixth wave of SARS-CoV-2 infections in Cyprus, primarily characterized by the Omicron 5 (BA.5.1 and BA.5.2) lineages, was estimated to have been introduced on 18 March 2022 (95% HPD: 24 January 2022–7 May 2022), although this estimate comes with a high degree of uncertainty. The initial import of Omicron 5 was found to be most likely from the USA (posterior probability of 0.31), albeit with low confidence, as Denmark followed closely with a posterior probability of 0.30. Additionally, the UK and Spain had relatively proximate posterior probabilities of 0.12 and 0.17, respectively, further emphasizing the uncertainty in pinpointing the origin. The dispersal of Omicron 5 Cypriot taxa was similar to that of Omicron 1 (Figure 6A,C), without any clades forming that would be indicative of inland transmission. This pattern was also apparent from the high numbers of imports and exports, particularly when compared to the Omicron 1 and Omicron 2 datasets (Figure 10). Omicron 5 was identified as having the highest number of exports among the three datasets, exceeding twenty per week, while the exports of Omicron 1 and Omicron 2 never surpassed 20 per week. Comparatively, the weekly imports of Omicron 5 may have never reached the highest levels of exports, but this variant was imported over a longer period. In contrast, exports of this variant only spiked from the end of June to July 2022 (as depicted in Figure 10). In fact, the total average number of imports was estimated at 165.84 (100%), while exports were estimated at 151.99 (100%), as shown in Table 2. Similar to Omicron 1 and Omicron 2, the USA was identified as having a prominent role in the traffic of Omicron 5 to and from Cyprus and was responsible for the majority of imports (58.46, 35.25%). The UK was the second most common source of Omicron 5 to Cyprus (39.41, 23.76%), while the other three of the top five sources of Omicron 5 to Cyprus were Finland (10.57, 6.37%), France (10.01, 6.04%), and Denmark (9.89, 5.96) (Table 2, Figure 9). Similarly, the exports were focused on North America as well as Northern and Western Europe. Specifically, the USA and UK were the two primary Omicron 5 sinks, with 35.13 (23.11%) and 30.91 (20.34%) exports, respectively. The remaining three of the top five export locations were Denmark (23.8, 15.66%), Germany (18.18, 11.96%), and Sweden (13.69, 9.01%) (Table 2, Figure 9). Interestingly, Omicron 5 was the only one out of the three datasets with minimal imports from Asia and essentially no exports to this continent (Table 2).

An important consideration in these analyses is the representation of sequences in our dataset and other sequences from different locations submitted to databases such as GISAID [70]. In these analyses, the USA and UK emerged as highly significant locations for import and export in relation to Cyprus, with approximately 4.5 million and 2.9 million SARS-CoV-2 sequences submitted from 2021 to the start of 2023, respectively [70]. In fact, 2.3 million of those submitted by the USA were classified as Omicron, while 1.4 million Omicron sequences were submitted by the UK. In contrast, other locations, such as Greece, which has long-standing cultural connections with Cyprus, had approximately 25 thousand submitted SARS-CoV-2 sequences over the same period, 16.3 thousand of which were Omicron sequences. Although there were approximately 21 times more sequences deposited after 2021 (1.2 thousand sequences submitted by December 2020), it is essential to keep in mind the absolute number of sequences submitted by specific locations.

## 4. Discussion

### 4.1. Overview of the SARS-CoV-2 Waves Identified in This Study

Within the scope of this investigation, a comprehensive analysis encompassing 4700 SARS-CoV-2 sequences was conducted. This endeavor culminated in the identification of a total of 167 distinct SARS-CoV-2 lineages and sublineages that were prevalent in Cyprus from October 2021 to October 2022. This marked a notable escalation compared to the preceding period, which spanned from November 2020 to October 2021. The earlier interval was characterized by the predominance of specific variants, namely B.1.258 (during the second wave), Alpha (during the third wave), and Delta (during the fourth wave). In this earlier phase, a relatively limited spectrum comprising only 61 distinct lineages and sublineages was observed [24]. This augmentation in genomic diversity coincided with the emergence of the Omicron variant in December 2021, which subsequently instigated the onset of the fifth and sixth waves of SARS-CoV-2 infections. Notably, during the fifth wave, the prominent subvariants were Omicron 1 and Omicron 2. As the sixth wave unfolded, Omicron 5 emerged as the prevailing subvariant. This temporal progression is effectively illustrated in Figure 1 and Figure 2.

### 4.2. Delta Variant Variability and Deltacron Genomes

At the initiation of the fifth wave, there was an observable upswing in infections linked to the Delta variant, as depicted in Figure 2. Throughout the relatively brief duration when the Delta variant exhibited prominence in this study—spanning from October to December 2021 and encompassing January 2022—an elevated level of variability within the Delta lineage was noted. This contrasted with the preceding period, spanning from November 2020 to October 2021 [24] (Figure S5).

Specifically, during the fourth wave and leading up to October 2021, a total of 34 distinct Delta lineages were identified. In contrast, within the scope of this study, a more comprehensive identification of 46 different Delta lineages was accomplished (as detailed in Table 1) [24]. As the pandemic evolved, this trend toward increasing variability within the Delta lineage persisted. This expansion of variability was compounded by the inherent propensity of the SARS-CoV-2 genome to accrue mutations. Consequently, the mutational distinctions among various Delta variants underwent significant amplification over this subsequent period [75].

During the progression of the fifth wave (December 2021–January 2022), a noteworthy development emerged wherein atypical mutations characteristic of the Omicron 1 variant were discerned within the Delta variants. To facilitate comprehension, this observation was informally designated “Deltacron”. The swift introduction of this term aimed to promptly communicate the phenomenon to the scientific community, thereby equipping health authorities with a proactive stance to safeguard public health. After conducting an exhaustive series of analyses aimed at unraveling this phenomenon (as detailed in Results Section 3.2.2) and considering the potential gravity of the implications involved, we concluded that it was judicious to adopt a transparent approach. Consequently, we promptly communicated the identification of these unprecedented mutations within Delta variants. Given the timeliness and urgency of these data, such transparency was considered appropriate. However, it is noteworthy that the news of this observation was disseminated rapidly and extensively through various media channels, including mass media and social platforms. Unfortunately, this dissemination led to a heightened sensationalization of the findings, generating a media frenzy that in turn caused unnecessary apprehension and concern among the general populace [76]. Consequently, a multitude of hypotheses arose to elucidate the matter, with two principal theories gaining prominence. The first theory posited the potential occurrence of a recombination event between the two variants, while the second theory postulated contamination or coinfection, potentially exacerbated by the suboptimal binding properties of the ARTIC V3 primers [74].

Nonetheless, through our extensive analyses, we effectively demonstrated that the anticipated sequencing outcome of a Delta sample harboring potential Omicron 1 contamination or coinfection would result in a frameshift. This frameshift would be attributed to the suboptimal binding of the ARTIC V3 right 72 primer with the Delta variant and the corresponding suboptimal binding of the ARTIC V3 left 73 primer with the Omicron 1 variant. This interaction would lead to a specific four-nucleotide deletion encompassing positions 21,987 to 21,990. Importantly, none of the sequences we submitted to the GISAID database exhibited this particular configuration. This evidence-based finding substantiates the conclusions derived from our investigation.

However, in a relatively short time, analogous Deltacron phenomena began to be documented across a multitude of nations, spanning from France, the Netherlands, Denmark, and the United Kingdom to Brazil and the United States, among others [77,78,79,80,81,82,83,84,85,86,87]. Specifically, occurrences of recombination between the Delta (AY.4, AY.x) and Omicron (BA.1, BA.1.1) variants were identified, and these recombinants were designated XD, XF, and XS, with the letter “X” denoting the recombination event itself. These Delta-Omicron recombinant entities were characterized as genetic mosaics, combining features of both Delta and Omicron genomes. XD, for instance, showcased the Omicron spike protein mutations in conjunction with a Delta backbone. In other instances, breakpoints within the ORF1ab region were observed, with the remainder of the genome reflecting Omicron characteristics [77,78,79,80,81,82,83,84,85,86,87,88,89,90,91]. These discoveries garnered considerable attention and were subsequently monitored by the World Health Organization (WHO). Comprehensive investigations were launched to ascertain the potential risks posed by these recombinants and to evaluate the effectiveness of existing vaccines in addressing them [55,80,88,89,91].

Consequently, the evolution of the concept of Deltacron can be traced from the initial identification of Omicron signatures within Delta variants to a sequence of stages encompassing controversy, gradual acceptance, recognition as a potential threat, and eventually becoming a focal point for numerous scientific publications and a subject of mass and social media coverage [76,77,78,79,80,81,82,83,84,85,86,87]. Despite the inherent risk of social media’s propensity to amplify and propagate misinformation, a challenge that could inadvertently fuel hesitancy in promptly sharing time-sensitive scientific data, we decided to disseminate our initial findings [92,93]. This choice played a pivotal role in highlighting an important phenomenon, elevating awareness, and fostering heightened vigilance among relevant stakeholders [22,74,84,85,86,87]. Our commitment bore fruit, as the scientific community adeptly navigated through the noise and distortion generated by social media amplification, thereby effectively addressing the implications of the cocirculation of two of the most infectious and impactful variants that have emerged during this pandemic [79,80,94,95].

### 4.3. The First Peak of the Fifth Wave: Omicron 1

The emergence of Omicron and its import into Cyprus (December 2021) quickly increased from the number of SARS-CoV-2 cases, and the generation of the fifth wave started with Omicron 1. Our analyses showed that Omicron 1 was first imported to Cyprus from the USA, and so were the majority of imports, with the UK being the second major import location (Figure 7, Table 2). The Ministry of Health mounted a quick response against Omicron through policies implemented to halt its further spread. Starting on the 15th of December 2021, these policies included the mandatory isolation of individuals positive for Omicron and their close contacts, regardless of vaccination status [96]. Furthermore, certain holiday events were canceled, and social events/aggregations such as weddings, sports events, and theaters were only allowed for vaccinated individuals with a negative test. The testing of passengers arriving in Cyprus following a 72 h period from their arrival was also required [96]. Additional policies were instated as Omicron spread rapidly both in Cyprus and around the globe [96,97,98]. Nonetheless, despite the policies implemented in Cyprus, SARS-CoV-2 cases continued to increase, reaching 107 thousand cases (January 2021), whereas even when the infamous Delta variant was prevalent, monthly cases never exceeded 25 thousand (Figure 1A). This unprecedentedly high number of cases, driven by Omicron 1, signified the first peak of the fifth wave in Cyprus. In fact, Omicron was the most dominant in a plethora of countries, including the UK and USA, despite the measures and travel restrictions placed to circumvent the progression of this variant [99,100]. However, it is important to keep in mind that Omicron was by far the most infectious variant to ever emerge during this pandemic, and it would be daunting to consider the cases that would have resulted without these quick interventions and measures by governments and policymakers [97,98]. As with every new variant that emerged, it was followed by a new wave of infections and a corresponding increase in mortality. In response, strict public health measures were implemented, with which the public largely complied, which resulted in a decrease in cases. However, with each successive wave, compliance decreased, largely due to accumulated fatigue, even in the face of the emergence of new variants [101].

### 4.4. The Second Peak of the Fifth Wave: Omicron 2

The continued efforts to stop the initial torrent of Omicron 1 infections were proven successful, and the ongoing transmission was stymied in February 2022. Halting the increase in new infections and stabilizing the situation was met with the cautious alleviation (announced on 15 February 2022) of implementation placed during December 2021 and January 2022 with respect to citizen compliance and in efforts to resume normalcy [96]. However, after February, infections started increasing again, and as with every surge of new infections in the past, this one was underlined by a new variant/lineage. In this case, this was Omicron 2, which eventually resulted in the second peak of the fifth wave in March 2022 and ultimately declined by April 2022 (Figure 1 and Figure 2). The UK was estimated to be the origin of Omicron 2 in Cyprus and accounted for the majority of imports to the island. Apart from the UK, most of the imports were from the European region, specifically Germany, Greece, and Spain, while the USA was again identified as an import location (Figure 8, Table 2). As with Omicron 1, a similar situation also occurred in various parts of the world, with a surge of new infections driven by Omicron 2, which was reported to be more transmissible than Omicron 1 [98,102,103]. Following the decline in Omicron 2 SARS-CoV-2 cases after April 2022 and with the announcement of the third and fourth booster vaccines (4 April 2022), the prudent easement of measures was steadily underway (7 April 2022) [96]. These measures entailed the diminution of venues requiring proof of vaccination and/or negative PCR/rapid tests, such as nonmedical workplaces, provided proper health regulation and self-tests were performed [96]. Additionally, the release of individuals positive for SARS-CoV-2 from isolation was reduced to seven days, provided there was a negative test at the end of that period [96]. The change in public health policies aimed to gradually support the transition of the public to a near-normal lifestyle while still being respectful of the still-circulating SARS-CoV-2.

### 4.5. The Sixth Wave: Omicron 5

The well-being of the community, lessening “pandemic fatigue”, and allowing each individual to exercise self-protection against the virus using the accumulated knowledge and strategies taught up to that point, were key to returning to normalcy (Ministry announcements 20 April, 10 May, and 13 May 2022) [96,104,105]. Indeed, following the fifth wave with Omicron 1 and 2, the number of cases drastically decreased. However, during the summer of 2022 (June–July), there was a moderate and brief increase in cases, which denoted the sixth wave, underlined by mostly Omicron 5 infections, with Omicron 4 accounting for only a minority of the infections (Figure 1 and Figure 2). This strengthened the premise that seasonality was not the strongest factor for the increase in SARS-CoV-2 cases since waves of infection emerged at different times of the year (Figure 1 and Figure 2) [24,106,107]. Moreover, the generation of new waves of infection was more dependent on the emergence of new, more infectious variants with different antigenic profiles than the currently circulating variants [14,108,109]. In fact, similar to the fifth wave, the USA was identified both as an origin location for Omicron 5 and as comprising the majority of imports along with the UK (Figure 9, Table 2). This situation also occurred worldwide, with new waves of infection being driven by Omicron 5 (and/or Omicron 4) in a plethora of countries [110,111]. The Cypriot self-protection policies continued, despite the increase in cases, with the directed measures (28 June 2022) for self-protection being upgraded to the mandatory use of masks around vulnerable populations and healthcare facilities [96]. The sixth wave with Omicron 5 declined in August 2022, with no notable increases in the number of infections until the end of the sampling period.

### 4.6. The Dominance of Omicron

The Omicron variant has dominated over all other variants in Cyprus and worldwide since its identification in November 2021. During the time of this study and since Omicron was identified, there were approximately 3.2 million new tourist arrivals in Cyprus [112]. In contrast, during the previous study period (November 2020–October 2021), there were almost half the number of new arrivals (approximately 1.7 million) [24,112]. With no lockdowns in place, the increase in new arrivals from all over the world resulted in a higher impact of new SARS-CoV-2 infections, underscoring the significant global impact of the variant on the island, with similar scenarios occurring at a global scale [98,99,100,102,103,110,111]. Nonetheless, the infectivity of Omicron was so immense that despite any measures placed, its spread could not be abrogated; however, the measures and policies probably protected people from an even higher burden [99,100]. Yet, despite Omicron’s higher infectivity and spread, it has led to relatively fewer hospitalizations and deaths in contrast to other variants, such as Delta [99,113,114,115]. The lower virulence of Omicron does not constitute a reason for complacency since, with its exceptionally high variability, the emergence of a variant that shares its infectivity but has a higher virulence is entirely possible [108]. After all, this scenario occurred in the past with Alpha and Delta [108]. Omicron was also reported to have been associated with reduced vaccine effectiveness, and natural immunity could not provide adequate protection, even when the individual was previously infected with non-Omicron variants [116,117]. Thus, it is important to consider the molecular characteristics of the Omicron genome, which made this variant the most prolific and diverse variant to have ever emerged. In fact, as of August 2023, over 1000 Omicron lineages and sublineages have been identified, including recombinants [118]. Within Omicron, and depending on lineage, there are at least 30 mutations in the S gene, which have a large impact on immune evasion and the transmissibility of SARS-CoV-2 [119]. Omicron mutations such as K417N, L452RQ, S477N, T478K, E484A, F486V, Q493R, N501Y, and Y505H have been associated with immune evasion [120]. Some mutations were also reported to increase transmissibility apart from immune evasion, including but not limited to N501Y [121]. Other mutations that increase transmissibility are Q498R, H655Y, N679K, and P681H, since they can improve cell receptor attachment [121]. Several variants of Omicron, such as BQ, BA.2.75, BA.2.12.1, and XBB, have also emerged, which have further acquired high-risk mutations, such as R346T, K444T, N460K, and L452R/Q [122].

As the pandemic progressed, Omicron continued to dominate even after the sixth wave, albeit with a substantial drop in new infections. The WHO also declared the end of the COVID-19 emergency phase (5 May 2023), and currently (August 2023), only subvariants of XBB are primarily circulating in Cyprus; however, none have resulted in a new wave of infections in Cyprus akin to previous waves [15,16,17,123].

### 4.7. Conclusions

To conclude, this study describes the SARS-CoV-2 epidemic within Cyprus, spanning from October 2021 to October 2022. Over this period, the Delta and Omicron variants (including subvariants 1, 2, and 5) were identified, consequently instigating the emergence of the fifth and sixth waves of SARS-CoV-2 infections within Cyprus. Specifically, the fifth wave was initially propelled by Omicron 1, which was succeeded in short order by Omicron 2. The sixth wave, on the other hand, was characterized by the dominance of Omicron 5. While the Delta variant was notable at the outset of the study period, it was gradually overshadowed by the ascendancy of Omicron, especially toward the conclusion of the fifth wave. Through spatiotemporal analyses, the United States and the United Kingdom emerged as the primary sources of SARS-CoV-2 spread to and from Cyprus. Other locations, including Greece, South Korea, France, Germany, Brazil, Spain, Australia, Denmark, Sweden, and Italy, exhibited a lesser degree of implication in terms of transmission dynamics. As COVID-19 enters an era characterized by a diverse range of variants, often with lower numbers of cases and hospitalizations compared to prior waves, it remains crucial to uphold vigilance. This requires a sustained effort in surveillance and diagnostic testing, enriched by comprehensive genomic epidemiological studies. These layers of data will enable public health authorities to make informed decisions, ranging from the imposition of travel bans to the initiation of targeted self-protection measures. Such proactive steps are particularly crucial when there is a potential upswing in infections, driven by the emergence of new variants with altered antigenic properties or increased transmissibility. Drawing upon the lessons of the past, it is vital to be prepared for such potential scenarios that could threaten public health in the future.

## Figures and Tables

**Table 1:** SARS-CoV-2 lineages identified from 4700 sequences in Cyprus from October 2021 to October 2022.

Time Period	Oct–Dec 2021	Jan–Mar 2022	Apr–June 2022	Jul–Oct 2022	Total
Lineage	Νumber of Sequences per Lineage (%)	Νumber of Sequences per Lineage (%)	Νumber of Sequences per Lineage (%)	Νumber of Sequences per Lineage (%)	Νumber of Sequences per Lineage (%)
AY.3	2 (0.23)	-	-	-	2 (0.04)
AY.4	103 (12.02)	3 (0.14)	-	-	106 (2.26)
AY.4.2	33 (3.85)	5 (0.23)	-	-	38 (0.81)
AY.4.2.1	3 (0.35)	-	-	-	3 (0.06)
AY.4.2.5	-	1 (0.05)	-	-	1 (0.02)
AY.4.5	4 (0.47)	-	-	-	4 (0.09)
AY.4.6	1 (0.12)	-	-	-	1 (0.02)
AY.5	3 (0.35)	-	-	-	3 (0.06)
AY.6	2 (0.23)	-	-	-	2 (0.04)
AY.7.2	19 (2.22)	3 (0.14)	-	-	22 (0.47)
AY.9.2	5 (0.58)	-	-	-	5 (0.11)
AY.23	1 (0.12)	-	-	-	1 (0.02)
AY.25	1 (0.12)	-	-	-	1 (0.02)
AY.33	1 (0.12)	-	-	-	1 (0.02)
AY.34	1 (0.12)	-	-	-	1 (0.02)
AY.34.1	1 (0.12)	-	-	-	1 (0.02)
AY.36	4 (0.47)	-	-	-	4 (0.09)
AY.42	1 (0.12)	-	-	-	1 (0.02)
AY.43	192 (22.4)	5 (0.23)	-	-	197 (4.19)
AY.43.8	1 (0.12)	-	-	-	1 (0.02)
AY.46.5	2 (0.23)	-	-	-	2 (0.04)
AY.46.6	4 (0.47)	-	-	-	4 (0.09)
AY.60	2 (0.23)	-	-	-	2 (0.04)
AY.98	32 (3.73)	-	-	-	32 (0.68)
AY.98.1	11 (1.28)	-	-	-	11 (0.23)
AY.103	4 (0.47)	-	-	-	4 (0.09)
AY.106	1 (0.12)	-	-	-	1 (0.02)
AY.107	-	1 (0.05)	-	-	1 (0.02)
AY.109	1 (0.12)	-	-	-	1 (0.02)
AY.111	1 (0.12)	-	-	-	1 (0.02)
AY.114	1 (0.12)	-	-	-	1 (0.02)
AY.116	1 (0.12)	-	-	-	1 (0.02)
AY.119.2	1 (0.12)	-	-	-	1 (0.02)
AY.120	2 (0.23)	-	-	-	2 (0.04)
AY.120.2.1	1 (0.12)	-	-	-	1 (0.02)
AY.121	35 (4.08)	10 (0.47)	-	-	45 (0.96)
AY.122	106 (12.37)	9 (0.42)	-	-	115 (2.45)
AY.124	3 (0.35)	-	-	-	3 (0.06)
AY.124.1	1 (0.12)	-	-	-	1 (0.02)
AY.125	3 (0.35)	-	-	-	3 (0.06)
AY.126	17 (1.98)	1 (0.05)	-	-	18 (0.38)
AY.127	33 (3.85)	11 (0.52)	-	-	44 (0.94)
AY.127.2	1 (0.12)	-	-	-	1 (0.02)
AY.128	2 (0.23)	-	-	-	2 (0.04)
AY.129	1 (0.12)	-	-	-	1 (0.02)
B.1.617.2	43 (5.02)	4 (0.19)	-	-	47 (1)
BA.1	62 (7.23)	272 (12.76)	3 (0.31)	-	337 (7.17)
BA.1.1	23 (2.68)	492 (23.09)	14 (1.45)	-	529 (11.26)
BA.1.1.1	-	71 (3.33)	-	-	71 (1.51)
BA.1.1.11	1 (0.12)	1 (0.05)	-	-	2 (0.04)
BA.1.1.14	-	-	1 (0.10)	-	1 (0.02)
BA.1.1.15	7 (0.82)	150 (7.04)	-	-	157 (3.34)
BA.1.1.18	-	2 (0.09)	-	-	2 (0.04)
BA.1.7	-	1 (0.05)	-	-	1 (0.02)
BA.1.8	-	1 (0.05)	-	-	1 (0.02)
BA.1.10	1 (0.12)	-	-	-	1 (0.02)
BA.1.13	-	1 (0.05)	1 (0.10)	-	2 (0.04)
BA.1.15	9 (1.05)	16 (0.75)	-	-	25 (0.53)
BA.1.15.1	8 (0.93)	6 (0.28)	-	-	14 (0.3)
BA.1.16	2 (0.23)	14 (0.66)	-	-	16 (0.34)
BA.1.17	14 (1.63)	84 (3.94)	1 (0.10)	-	99 (2.11)
BA.1.17.2	39 (4.55)	155 (7.27)	-	-	194 (4.13)
BA.1.18	2 (0.23)	12 (0.56)	-	-	14 (0.3)
BA.1.19	1 (0.12)	1 (0.05)	-	-	2 (0.04)
BA.1.20	-	2 (0.09)	-	-	2 (0.04)
BA.1.21	-	1 (0.05)	-	-	1 (0.02)
BA.2	1 (0.12)	660 (30.97)	522 (53.98)	2 (0.27)	1185 (25.21)
BA.2.1	-	1 (0.05)	8 (0.83)	-	9 (0.19)
BA.2.2	-	-	1 (0.10)	-	1 (0.02)
BA.2.3	-	23 (1.08)	26 (2.69)	-	49 (1.04)
BA.2.3.15	-	-	1 (0.10)	-	1 (0.02)
BA.2.3.20	-	-	-	6 (0.81)	6 (0.13)
BA.2.5	-	1 (0.05)	4 (0.41)	-	5 (0.11)
BA.2.9	-	68 (3.19)	60 (6.2)	-	128 (2.72)
BA.2.9.3	-	-	1 (0.10)	1 (0.13)	2 (0.04)
BA.2.9.5	-	2 (0.09)	-	-	2 (0.04)
BA.2.10	-	5 (0.23)	3 (0.31)	-	8 (0.17)
BA.2.10.3	-	-	1 (0.10)	-	1 (0.02)
BA.2.12.1	-	-	43 (4.45)	4 (0.54)	47 (1)
BA.2.18	-	1 (0.05)	2 (0.21)	-	3 (0.06)
BA.2.19	-	-	2 (0.21)	-	2 (0.04)
BA.2.23	-	-	1 (0.10)	-	1 (0.02)
BA.2.23.1	-	-	1 (0.10)	-	1 (0.02)
BA.2.31	-	-	1 (0.10)	-	1 (0.02)
BA.2.36	-	-	3 (0.31)	-	3 (0.06)
BA.2.37	-	32 (1.5)	13 (1.34)	-	45 (0.96)
BA.2.38	-	-	-	2 (0.27)	2 (0.04)
BA.2.39	-	1 (0.05)	-	-	1 (0.02)
BA.2.41	-	-	1 (0.10)	-	1 (0.02)
BA.2.56	-	-	1 (0.10)	-	1 (0.02)
BA.2.63	-	1 (0.05)	2 (0.21)	-	3 (0.06)
BA.2.65	-	1 (0.05)	5 (0.52)	-	6 (0.13)
BA.2.68	-	-	1 (0.10)	-	1 (0.02)
BA.2.71	-	-	4 (0.41)	-	4 (0.09)
BA.2.72	-	-	1 (0.10)	-	1 (0.02)
BN.1.3	-	-	-	1 (0.13)	1 (0.02)
BA.2.78	-	-	1 (0.10)	-	1 (0.02)
XBB.1	-	-	-	2 (0.27)	2 (0.04)
XBB.2	-	-	-	1 (0.13)	1 (0.02)
XL	-	-	1 (0.10)	-	1 (0.02)
XAL	-	-	3 (0.31)	-	3 (0.06)
BA.4	-	-	24 (2.48)	24 (3.22)	48 (1.02)
BA.4.1	-	-	11 (1.14)	11 (1.48)	22 (0.47)
BA.4.1.1	-	-	1 (0.10)	-	1 (0.02)
BA.4.1.5	-	-	-	1 (0.13)	1 (0.02)
BA.4.4	-	-	2 (0.21)	1 (0.13)	3 (0.06)
BA.4.6	-	-	-	2 (0.27)	2 (0.04)
BA.4.7	-	-	-	1 (0.13)	1 (0.02)
BA.5	-	-	1 (0.10)	5 (0.67)	6 (0.13)
BA.5.1	-	-	88 (9.1)	170 (22.82)	258 (5.49)
BA.5.1.1	-	-	1 (0.10)	-	1 (0.02)
BA.5.1.2	-	-	4 (0.41)	4 (0.54)	8 (0.17)
BA.5.1.3	-	-	3 (0.31)	10 (1.34)	13 (0.28)
BA.5.1.5	-	-	-	3 (0.40)	3 (0.06)
BA.5.1.10	-	-	-	2 (0.27)	2 (0.04)
BK.1	-	-	-	1 (0.13)	1 (0.02)
BA.5.1.12	-	-	-	3 (0.40)	3 (0.06)
BA.5.1.17	-	-	-	1 (0.13)	1 (0.02)
BA.5.1.22	-	-	-	2 (0.27)	2 (0.04)
BA.5.1.23	-	-	-	2 (0.27)	2 (0.04)
BA.5.2	-	-	40 (4.14)	183 (24.56)	223 (4.74)
BA.5.2.1	-	-	32 (3.31)	107 (14.36)	139 (2.96)
BF.1	-	-	6 (0.62)	5 (0.67)	11 (0.23)
BF.2	-	-	-	1 (0.13)	1 (0.02)
BF.4	-	-	-	3 (0.40)	3 (0.06)
BF.5	-	-	2 (0.21)	23 (3.09)	25 (0.53)
BF.7	-	-	-	15 (2.01)	15 (0.32)
BF.7.1	-	-	-	2 (0.27)	2 (0.04)
BF.7.4.2	-	-	-	1 (0.13)	1 (0.02)
BF.7.5	-	-	-	1 (0.13)	1 (0.02)
BF.7.5.1	-	-	-	1 (0.13)	1 (0.02)
BF.7.8	-	-	-	1 (0.13)	1 (0.02)
BF.10	-	-	-	5 (0.67)	5 (0.11)
BF.11	-	-	-	11 (1.48)	11 (0.23)
BF.11.2	-	-	-	1 (0.13)	1 (0.02)
BF.14	-	-	-	1 (0.13)	1 (0.02)
BF.19	-	-	-	1 (0.13)	1 (0.02)
BA.5.2.2	-	-	-	1 (0.13)	1 (0.02)
BA.5.2.3	-	-	1 (0.10)	4 (0.54)	5 (0.11)
BA.5.2.13	-	-	-	1 (0.13)	1 (0.02)
BA.5.2.14	-	-	-	1 (0.13)	1 (0.02)
BA.5.2.18	-	-	-	1 (0.13)	1 (0.02)
BA.5.2.20	-	-	-	12 (1.61)	12 (0.26)
BA.5.2.22	-	-	-	1 (0.13)	1 (0.02)
CK.2	-	-	-	1 (0.13)	1 (0.02)
BA.5.2.26	-	-	-	1 (0.13)	1 (0.02)
BA.5.2.33	-	-	-	2 (0.27)	2 (0.04)
BA.5.2.35	-	-	-	1 (0.13)	1 (0.02)
BA.5.3	-	-	1 (0.10)	-	1 (0.02)
BA.5.3.1	-	-	-	1 (0.13)	1 (0.02)
BE.1	-	-	9 (0.93)	21 (2.82)	30 (0.64)
BE.1.1	-	-	1 (0.10)	27 (3.62)	28 (0.6)
BE.1.1.1	-	-	-	4 (0.54)	4 (0.09)
BQ.1	-	-	-	2 (0.27)	2 (0.04)
BQ.1.1	-	-	-	7 (0.94)	7 (0.15)
BQ.1.1.1	-	-	-	1 (0.13)	1 (0.02)
BQ.1.1.11	-	-	-	2 (0.27)	2 (0.04)
BQ.1.3	-	-	-	1 (0.13)	1 (0.02)
BQ.1.5	-	-	-	2 (0.27)	2 (0.04)
BQ.1.8	-	-	-	1 (0.13)	1 (0.02)
BA.5.3.2	-	-	1 (0.10)	-	1 (0.02)
BA.5.3.3	-	-	1 (0.10)	6 (0.81)	7 (0.15)
BA.5.3.4	-	-	-	1 (0.13)	1 (0.02)
BA.5.5	-	-	2 (0.21)	4 (0.54)	6 (0.13)
BA.5.6	-	-	2 (0.21)	3 (0.40)	5 (0.11)
BA.5.8	-	-	1 (0.10)	1 (0.13)	2 (0.04)
BA.5.9	-	-	-	11 (1.48)	11 (0.23)
Total	857	2131	967	745	4700

**Table 2:** The estimated number of migration events towards and from Cyprus.

Variant/Lineage ^a^	From ^b^	To ^c^	Average ^d^	Lower ^e^	Upper ^f^
**Omicron 1 (BA.1, BA.1.1)**	All ^g^	Cyprus	139.06	127	150
	USA	Cyprus	84.96	74	99
	UnitedKingdom	Cyprus	35.56	23	47
	SouthKorea	Cyprus	7.17	3	11
	SouthernEurope	Cyprus	5.24	2	9
	SouthEasternAsia	Cyprus	2.68	0	6
	NorthernEurope	Cyprus	1.47	0	9
	France	Cyprus	1.11	0	5
	Germany	Cyprus	0.87	0	5
	Cyprus	All	21.62	8	34
	Cyprus	WesternAsia	6.52	2	10
	Cyprus	UnitedKingdom	6.32	0	18
	Cyprus	Brazil	5.41	0	12
	Cyprus	Oceania	1.79	0	8
	Cyprus	USA	1.60	0	9
**Omicron 2 (BA.2)**	All	Cyprus	59.95	50	69
	UnitedKingdom	Cyprus	31.32	20	42
	Germany	Cyprus	15.04	8	22
	USA	Cyprus	10.02	3	17
	Greece	Cyprus	1.06	0	4
	Spain	Cyprus	1.06	0	2
	Australia	Cyprus	0.98	0	4
	EasternEurope	Cyprus	0.46	0	2
	Cyprus	All	61.55	52	71
	Cyprus	UnitedKingdom	40.61	30	50
	Cyprus	Greece	6.46	4	9
	Cyprus	WesternAsia	3.59	1	6
	Cyprus	WesternEurope	3.33	1	6
	Cyprus	Australia	3.00	0	8
	Cyprus	Oceania	1.82	0	3
	Cyprus	USA	1.42	0	8
	Cyprus	Mauritius	1.32	0	2
**Omicron 5 (BA.5.1, BA.5.2)**	All	Cyprus	165.84	146	194
	USA	Cyprus	58.46	37	79
	UnitedKingdom	Cyprus	39.41	22	55
	Finland	Cyprus	10.57	5	20
	France	Cyprus	10.01	0	19
	Denmark	Cyprus	9.89	3	23
	Spain	Cyprus	8.51	0	30
	Japan	Cyprus	7.96	3	12
	Germany	Cyprus	7.13	0	13
	Italy	Cyprus	5.53	0	10
	WesternAsia	Cyprus	2.75	0	7
	Israel	Cyprus	2.65	0	10
	Slovenia	Cyprus	1.29	0	3
	Australia	Cyprus	1.02	0	3
	EasternEurope	Cyprus	0.47	0	3
	WesternEurope	Cyprus	0.19	0	1
	Cyprus	All	151.99	127	186
	Cyprus	USA	35.13	18	52
	Cyprus	UnitedKingdom	30.91	18	44
	Cyprus	Denmark	23.80	15	32
	Cyprus	Germany	18.18	10	25
	Cyprus	Sweden	13.69	8	19
	Cyprus	Italy	8.29	0	14
	Cyprus	Greece	7.90	5	10
	Cyprus	WesternEurope	4.70	0	12
	Cyprus	Slovakia	2.39	0	8
	Cyprus	Curacao	1.80	0	2
	Cyprus	Russia	1.41	0	8
	Cyprus	EasternEurope	1.23	0	10
	Cyprus	Israel	1.02	0	8
	Cyprus	Slovenia	0.91	0	8
	Cyprus	SouthernEurope	0.63	0	4

^a^ The Pango system of classification, along with the WHO Greek alphabet nomenclature system, was used to indicate variants/lineages [5,14,61]. ^b,c^ “From” and “To” refer to countries/subregions where migration events began from or were directed to, respectively (country/subregion was based on a United Nations (UN) geographical subregion). ^d–f^ The average Markov jumps are based on the lower and upper bounds of the 95% HPD interval migration events towards and from Cyprus. ^g^ “All” represents the aggregation of the migration events.

## Data Availability

The SARS-CoV-2 sequences analyzed in this study will be made accessible upon publication of the manuscript. These sequences will be deposited in the GISAID database [70] for broader scientific access and utilization. It is important to note that only the subset of 4700 sequences that have been designated as of “good quality” under the parameter “qc.overallStatus” will be shared with GISAID. This selective approach is adopted to prevent potential misinterpretations of mutations that may arise from data that could have been generated during sequencing and assembly processes, as previously noted in our earlier study [24,27]. By sharing high-quality sequences, the research community can benefit from reliable and accurate genetic information pertaining to SARS-CoV-2, facilitating further research and insights into the virus.

## Supplementary Materials

The following are available online at https://www.mdpi.com/article/10.3390/v15091933/s1, Table S1. Common S protein mutations of Delta and Omicron lineages that were identified in this dataset. Table S2. Uncommon S protein mutations of Delta and Omicron lineages that were identified in this dataset [124]. Figure S1. The postulated sequencing results from ARTIC V3 primer pairs 72 and 73. Figure S2. The sequencing results from ARTIC V3 primer pairs 72 and 73 of a Delta sample with a frameshift due to a 4-nucleotide deletion at positions 21,987–21,990. Figure S3. The sequencing results from ARTIC V3 primer pairs 72 and 73 of an Omicron 1 sample with a frameshift due to a 4-nucleotide deletion at positions 21,987–21,990. Figure S4. Alignment of representative Deltacron sequences from Cyprus and other countries. Figure S5. Maximum likelihood phylogenetic tree construction encompassing Delta variants from the fourth and fifth waves of SARS-CoV-2 infection in Cyprus.

## References

[1] Rabi F.A., Al Zoubi M.S., Al-Nasser A.D., Kasasbeh G.A., Salameh D.M. (2020). Sars-cov-2 and coronavirus disease 2019: What we know so far. Pathogens.

[2] Nicola M., Alsafi Z., Sohrabi C., Kerwan A., Al-Jabir A., Iosifidis C., Agha M., Agha R. (2020). The socio-economic implications of the coronavirus pandemic (COVID-19): A review. Int. J. Surg..

[3] Dong E., Du H., Gardner L. (2020). An interactive web-based dashboard to track COVID-19 in real time. Lancet Infect. Dis..

[4] Center for Systems Science and Engineering (CSSE) at Johns Hopkins University (JHU). COVID-19 Dashboard. https://gisanddata.maps.arcgis.com/apps/dashboards/bda7594740fd40299423467b48e9ecf6.

[5] The World Health Organization (WHO) Tracking SARS-CoV-2 Variants. https://www.who.int/activities/tracking-SARS-CoV-2-variants.

[6] Kumar A., Parashar R., Kumar S., Faiq M.A., Kumari C., Kulandhasamy M., Narayan R.K., Jha R.K., Singh H.N., Prasoon P. (2022). Emerging SARS-CoV-2 variants can potentially break set epidemiological barriers in COVID-19. J. Med. Virol..

[7] Ahmad A., Fawaz M.A.M., Aisha A. (2022). A comparative overview of SARS-CoV-2 and its variants of concern. Le Infez. Med..

[8] Tosta S., Moreno K., Schuab G., Fonseca V., Segovia F.M.C., Kashima S., Elias M.C., Sampaio S.C., Ciccozzi M., Alcantara L.C.J. (2023). Global SARS-CoV-2 genomic surveillance: What we have learned (so far). Infect. Genet. Evol..

[9] Wagner A.L. What Makes a “Wave” of Disease? An Epidemiologist Explains. https://theconversation.com/what-makes-a-wave-of-disease-an-epidemiologist-explains-141573.

[10] da Silva S.J.R., Kohl A., Pena L., Pardee K. (2023). Recent insights into SARS-CoV-2 omicron variant. Rev. Med. Virol..

[11] Tatsi E.-B., Filippatos F., Michos A. (2021). SARS-CoV-2 variants and effectiveness of vaccines: A review of current evidence. Epidemiol. Infect..

[12] Viana R., Moyo S., Amoako D.G., Tegally H., Scheepers C., Althaus C.L., Anyaneji U.J., Bester P.A., Boni M.F., Chand M. (2022). Rapid epidemic expansion of the SARS-CoV-2 Omicron variant in southern Africa. Nature.

[13] Islam F., Dhawan M., Nafady M.H., Emran T.B., Mitra S., Choudhary O.P., Akter A. (2022). Understanding the omicron variant (B.1.1.529) of SARS-CoV-2: Mutational impacts, concerns, and the possible solutions. Ann. Med. Surg..

[14] Markov P.V., Ghafari M., Beer M., Lythgoe K., Simmonds P., Stilianakis N.I., Katzourakis A. (2023). The evolution of SARS-CoV-2. Nat. Rev. Microbiol..

[15] Karyakarte R.P., Das R., Rajmane M.V., Dudhate S., Agarasen J., Pillai P., Chandankhede P.M., Labhshetwar R.S., Gadiyal Y., Kulkarni P.P. (2023). Chasing SARS-CoV-2 XBB.1.16 Recombinant Lineage in India and the Clinical Profile of XBB.1.16 cases in Maharashtra, India. medRxiv.

[16] Callaway E. (2022). COVID “variant soup” is making winter surges hard to predict. Nature.

[17] Callaway E. (2023). COVID’s future: Mini-waves rather than seasonal surges. Nature.

[18] Parra-Lucares A., Segura P., Rojas V., Pumarino C., Saint-Pierre G., Toro L. (2022). Emergence of SARS-CoV-2 Variants in the World: How Could This Happen?. Life.

[19] Rodrigo G.-L., Estibalitz L.-S., Roselyn L.-M., Alejandro S.-F., Carlos S.-V. (2022). The New SARS-CoV-2 Variants and Their Epidemiological Impact in Mexico. MBio.

[20] Andrés C., Piñana M., Borràs-Bermejo B., González-Sánchez A., García-Cehic D., Esperalba J., Rando A., Zules-Oña R.-G., Campos C., Codina M.G. (2022). A year living with SARS-CoV-2: An epidemiological overview of viral lineage circulation by whole-genome sequencing in Barcelona city (Catalonia, Spain). Emerg. Microbes Infect..

[21] da Silva M.S., Gularte J.S., Filippi M., Demoliner M., Girardi V., Mosena A.C.S., de Abreu Góes Pereira V.M., Hansen A.W., Weber M.N., de Almeida P.R. (2022). Genomic and epidemiologic surveillance of SARS-CoV-2 in Southern Brazil and identification of a new Omicron-L452R sublineage. Virus Res..

[22] Menezes D., Fonseca P.L., de Araújo J.L., Souza R.P. (2022). SARS-CoV-2 Genomic Surveillance in Brazil: A Systematic Review with Scientometric Analysis. Viruses.

[23] The COVID-19 Genomics UK (COG-UK) Consortium (2020). An integrated national scale SARS-CoV-2 genomic surveillance network. Lancet Microbe.

[24] Chrysostomou A.C., Vrancken B., Haralambous C., Alexandrou M., Aristokleous A., Christodoulou C., Gregoriou I., Ioannides M., Kalakouta O., Karagiannis C. (2022). Genomic Epidemiology of the SARS-CoV-2 Epidemic in Cyprus from November 2020 to October 2021: The Passage of Waves of Alpha and Delta Variants of Concern. Viruses.

[25] Chrysostomou A.C., Vrancken B., Koumbaris G., Themistokleous G., Aristokleous A., Masia C., Eleftheriou C., Ioannou C., Stylianou D.C., Ioannides M. (2021). A Comprehensive Molecular Epidemiological Analysis of SARS-CoV-2 Infection in Cyprus from April 2020 to January 2021: Evidence of a Highly Polyphyletic and Evolving Epidemic. Viruses.

[26] O’Toole Á., Scher E., Underwood A., Jackson B., Hill V., McCrone J.T., Colquhoun R., Ruis C., Abu-Dahab K., Taylor B. (2021). Assignment of epidemiological lineages in an emerging pandemic using the pangolin tool. Virus Evol..

[27] Aksamentov I., Roemer C., Hodcroft E., Neher R. (2021). Nextclade: Clade assignment, mutation calling and quality control for viral genomes. J. Open Source Softw..

[28] Suzuki Y., Nishimura M., Inoue T., Kobayashi Y. (2020). Methods for reducing the number of sequences in molecular evolutionary analyses. Meta Gene.

[29] Katoh K., Standley D.M. (2013). MAFFT multiple sequence alignment software version 7: Improvements in performance and usability. Mol. Biol. Evol..

[30] Larsson A. (2014). AliView: A fast and lightweight alignment viewer and editor for large datasets. Bioinformatics.

[31] Nguyen L.T., Schmidt H.A., Von Haeseler A., Minh B.Q. (2015). IQ-TREE: A fast and effective stochastic algorithm for estimating maximum-likelihood phylogenies. Mol. Biol. Evol..

[32] Guindon S., Dufayard J.F., Lefort V., Anisimova M., Hordijk W., Gascuel O. (2010). New algorithms and methods to estimate maximum-likelihood phylogenies: Assessing the performance of PhyML 3.0. Syst. Biol..

[33] Hoang D.T., Chernomor O., Von Haeseler A., Minh B.Q., Vinh L.S. (2018). UFBoot2: Improving the ultrafast bootstrap approximation. Mol. Biol. Evol..

[34] To T.-H., Jung M., Lycett S., Gascuel O. (2016). Fast Dating Using Least-Squares Criteria and Algorithms. Syst. Biol..

[35] Lemey P., Rambaut A., Drummond A.J., Suchard M.A. (2009). Bayesian phylogeography finds its roots. PLoS Comput. Biol..

[36] Lemey P., Rambaut A., Bedford T., Faria N., Bielejec F., Baele G., Russell C.A., Smith D.J., Pybus O.G., Brockmann D. (2014). Unifying Viral Genetics and Human Transportation Data to Predict the Global Transmission Dynamics of Human Influenza H3N2. PLoS Pathog..

[37] Minin V.N., Suchard M.A. (2008). Fast, accurate and simulation-free stochastic mapping. Philos. Trans. R. Soc. B Biol. Sci..

[38] KIOS Research and Innovation Center of Excellence (KIOS CoE) H εξάπλωση της COVID-19 στη Κύπρο (The Spread of COVID-19 in Cyprus). https://covid19.ucy.ac.cy/.

[39] Press and Information Office Aνακοινωθέντα (Press Releases)-Aνακοίνωση του Υπουργείου Υγείας για νέα περιστατικά της νόσου COVID-19 (Announcement of the Ministry of Health of New COVID-19 Incidents. https://www.pio.gov.cy/ανακοινωθέντα/?keyword=Aνακοίνωση+του+Υπουργείου+Υγείας+για+νέα+περιστατικά+της+νόσου+COVID-19&startdate=&enddate=&category=&submitbtn=Aναζήτηση.

[40] Centers for Disease Control and Prevention (CDC) Calculating SARS-CoV-2 Laboratory Test Percent Positivity: CDC Methods and Considerations for Comparisons and Interpretation. https://www.cdc.gov/coronavirus/2019-ncov/lab/resources/calculating-percent-positivity.html.

[41] Liu L., Wang P., Nair M.S., Yu J., Rapp M., Wang Q., Luo Y., Chan J.F.W., Sahi V., Figueroa A. (2020). Potent neutralizing antibodies against multiple epitopes on SARS-CoV-2 spike. Nature.

[42] Bateman A., Martin M.J., O’Donovan C., Magrane M., Alpi E., Antunes R., Bely B., Bingley M., Bonilla C., Britto R. (2017). UniProt: The universal protein knowledgebase. Nucleic Acids Res..

[43] Ma J., Acevedo A.C., Wang Q. (2021). High-Potency Polypeptide-based Interference for Coronavirus Spike Glycoproteins. bioRxiv.

[44] Khelashvili G., Plante A., Doktorova M., Weinstein H. (2021). Ca(2+)-dependent mechanism of membrane insertion and destabilization by the SARS-CoV-2 fusion peptide. Biophys. J..

[45] Wang P., Nair M.S., Liu L., Iketani S., Luo Y., Guo Y., Wang M., Yu J., Zhang B., Kwong P.D. (2021). Antibody Resistance of SARS-CoV-2 Variants B.1.351 and B.1.1.7. Nature.

[46] Kim S., Lee J.H., Lee S., Shim S., Nguyen T.T., Hwang J., Kim H., Choi Y.O., Hong J., Bae S. (2020). The progression of sars coronavirus 2 (SARS-CoV-2): Mutation in the receptor binding domain of spike gene. Immune Netw..

[47] Mittal A., Manjunath K., Ranjan R.K., Kaushik S., Kumar S., Verma V. (2020). COVID-19 pandemic: Insights into structure, function, and hACE2 receptor recognition by SARS-CoV-2. PLoS Pathog..

[48] Huang Y., Yang C., Xu X.f., Xu W., Liu S.w. (2020). Structural and functional properties of SARS-CoV-2 spike protein: Potential antivirus drug development for COVID-19. Acta Pharmacol. Sin..

[49] Xia X. (2021). Domains and Functions of Spike Protein in Sars-Cov-2 in the Context of Vaccine Design. Viruses.

[50] Gobeil S.M.C., Janowska K., McDowell S., Mansouri K., Parks R., Manne K., Stalls V., Kopp M.F., Henderson R., Edwards R.J. (2021). D614G Mutation Alters SARS-CoV-2 Spike Conformation and Enhances Protease Cleavage at the S1/S2 Junction. Cell Rep..

[51] Sasaki M., Uemura K., Sato A., Toba S., Sanaki T., Maenaka K., Hall W.W., Orba Y., Sawa H. (2021). SARS-CoV-2 variants with mutations at the S1/S2 cleavage site are generated in vitro during propagation in TMPRSS2-deficient cells. PLoS Pathog..

[52] Kapoor K., Chen T., Tajkhorshid E. (2022). Posttranslational modifications optimize the ability of SARS-CoV-2 spike for effective interaction with host cell receptors. Proc. Natl. Acad. Sci. USA.

[53] Magazine N., Zhang T., Wu Y., McGee M.C., Veggiani G., Huang W. (2022). Mutations and Evolution of the SARS-CoV-2 Spike Protein. Viruses.

[54] Hodcroft E.B. CoVariants: SARS-CoV-2 Mutations and Variants of Interest. https://covariants.org/.

[55] Shrestha L.B., Foster C., Rawlinson W., Tedla N., Bull R.A. (2022). Evolution of the SARS-CoV-2 omicron variants BA.1 to BA.5: Implications for immune escape and transmission. Rev. Med. Virol..

[56] Beheshti Namdar A., Keikha M. (2022). BA. 2.12.1 is a new omicron offshoot that is a highly contagious but not severe disease. Ann. Med. Surg..

[57] Parums D.V. (2022). Editorial: World Health Organization (WHO) Variants of Concern Lineages Under Monitoring (VOC-LUM) in Response to the Global Spread of Lineages and Sublineages of Omicron, or B.1.1.529, SARS-CoV-2. Med. Sci. Monit. Int. Med. J. Exp. Clin. Res..

[58] Philip A.M., Ahmed W.S., Biswas K.H. (2023). Reversal of the unique Q493R mutation increases the affinity of Omicron S1-RBD for ACE2. Comput. Struct. Biotechnol. J..

[59] Dijokaite-Guraliuc A., Das R., Zhou D., Ginn H.M., Liu C., Duyvesteyn H.M.E., Huo J., Nutalai R., Supasa P., Selvaraj M. (2023). Rapid escape of new SARS-CoV-2 Omicron variants from BA.2-directed antibody responses. Cell Rep..

[60] Chen C., Nadeau S., Yared M., Voinov P., Xie N., Roemer C., Stadler T. (2022). CoV-Spectrum: Analysis of globally shared SARS-CoV-2 data to identify and characterize new variants. Bioinformatics.

[61] Rambaut A., Holmes E.C., O’Toole Á., Hill V., McCrone J.T., Ruis C., du Plessis L., Pybus O.G. (2020). A dynamic nomenclature proposal for SARS-CoV-2 lineages to assist genomic epidemiology. Nat. Microbiol..

[62] Singh J.K., Anand S., Srivastava S.K. (2023). Is BF.7 more infectious than other Omicron subtypes: Insights from structural and simulation studies of BF.7 spike RBD variant. Int. J. Biol. Macromol..

[63] Chakraborty C., Bhattacharya M., Sharma A.R. (2022). Present variants of concern and variants of interest of severe acute respiratory syndrome coronavirus 2: Their significant mutations in S-glycoprotein, infectivity, re-infectivity, immune escape and vaccines activity. Rev. Med. Virol..

[64] Planas D., Bruel T., Staropoli I., Guivel-Benhassine F., Porrot F., Maes P., Grzelak L., Prot M., Mougari S., Planchais C. (2023). Resistance of Omicron subvariants BA.2.75.2, BA.4.6, and BQ.1.1 to neutralizing antibodies. Nat. Commun..

[65] Focosi D., Quiroga R., McConnell S., Johnson M.C., Casadevall A. (2023). Convergent Evolution in SARS-CoV-2 Spike Creates a Variant Soup from Which New COVID-19 Waves Emerge. Int. J. Mol. Sci..

[66] Saputri D.S., Li S., van Eerden F.J., Rozewicki J., Xu Z., Ismanto H.S., Davila A., Teraguchi S., Katoh K., Standley D.M. (2020). Flexible, Functional, and Familiar: Characteristics of SARS-CoV-2 Spike Protein Evolution. Front. Microbiol..

[67] Peng Q., Zhou R., Liu N., Wang H., Xu H., Zhao M., Yang D., Au K.-K., Huang H., Liu L. (2022). Naturally occurring spike mutations influence the infectivity and immunogenicity of SARS-CoV-2. Cell. Mol. Immunol..

[68] McCallum M., De Marco A., Lempp F.A., Tortorici M.A., Pinto D., Walls A.C., Beltramello M., Chen A., Liu Z., Zatta F. (2021). N-terminal domain antigenic mapping reveals a site of vulnerability for SARS-CoV-2. Cell.

[69] Carabelli A.M., Peacock T.P., Thorne L.G., Harvey W.T., Hughes J., de Silva T.I., Peacock S.J., Barclay W.S., de Silva T.I., Towers G.J. (2023). SARS-CoV-2 variant biology: Immune escape, transmission and fitness. Nat. Rev. Microbiol..

[70] Khare S., Gurry C., Freitas L., Schultz M.B., Bach G., Diallo A., Akite N., Ho J., Lee R.T.C., Yeo W. (2021). GISAID’s Role in Pandemic Response. China CDC Wkly..

[71] Artic-Network Artic-ncov2019. https://github.com/artic-network/artic-ncov2019/blob/master/primer_schemes/nCoV-2019/V3/nCoV-2019.tsv.

[72] Quick J., Loman N. (2020). RTIC Network: Artic. Network. https://artic.network/resources/ncov/ncov-amplicon-v3.pdf.

[73] Sanderson T., Barrett J.C. (2021). Variation at Spike position 142 in SARS-CoV-2 Delta genomes is a technical artifact caused by dropout of a sequencing amplicon. Wellcome Open Res..

[74] Kreier F. (2022). Deltacron: The story of the variant that wasn’t. Nature.

[75] Dhawan M., Sharma A., Priyanka, Thakur N., Rajkhowa T.K., Choudhary O.P. (2022). Delta variant (B.1.617.2) of SARS-CoV-2: Mutations, impact, challenges and possible solutions. Hum. Vaccin. Immunother..

[76] Robinson J., Banerjee I. (2022). Omicron: Subvariants, Flurona, Deltacron media misinformation and plot twists. J. Adv. Intern. Med..

[77] Wang J., Fatima Muhammad S., Aman S., Khan A., Munir S., Khan M., Mohammad A., Waheed Y., Munir M., Guo L. (2022). Structural communication fingerprinting and dynamic investigation of RBD-hACE2 complex from BA.1  × AY.4 recombinant variant (Deltacron) of SARS-CoV-2 to decipher the structural basis for enhanced transmission. J. Biomol. Struct. Dyn..

[78] Evans J.P., Qu P., Zeng C., Zheng Y.-M., Carlin C., Bednash J.S., Lozanski G., Mallampalli R.K., Saif L.J., Oltz E.M. (2022). Neutralization of the SARS-CoV-2 Deltacron and BA.3 Variants. N. Engl. J. Med..

[79] Farheen S., Araf Y., Tang Y.-D., Zheng C. (2022). The Deltacron conundrum: Its origin and potential health risks. J. Med. Virol..

[80] Maulud S.Q., Hasan D.A., Ali R.K., Rashid R.F., Saied A.A., Dhawan M., Priyanka, Choudhary O.P. (2022). Deltacron: Apprehending a new phase of the COVID-19 pandemic. Int. J. Surg..

[81] Moisan A., Mastrovito B., De Oliveira F., Martel M., Hedin H., Leoz M., Nesi N., Schaeffer J., Ar Gouilh M., Plantier J.-C. (2022). Evidence of Transmission and Circulation of Deltacron XD Recombinant Severe Acute Respiratory Syndrome Coronavirus 2 in Northwest France. Clin. Infect. Dis..

[82] Johnson R., Mangwana N., Sharma J.R., Muller C.J.F., Malemela K., Mashau F., Dias S., Ramharack P., Kinnear C., Glanzmann B. (2022). Delineating the Spread and Prevalence of SARS-CoV-2 Omicron Sublineages (BA.1–BA.5) and Deltacron Using Wastewater in the Western Cape, South Africa. J. Infect. Dis..

[83] Focosi D., Maggi F. (2022). Recombination in Coronaviruses, with a Focus on SARS-CoV-2. Viruses.

[84] Lacek K.A., Rambo-Martin B.L., Batra D., Zheng X.-Y., Hassell N., Sakaguchi H., Peacock T., Groves N., Keller M., Wilson M.M. (2022). SARS-CoV-2 Delta-Omicron Recombinant Viruses, United States. Emerg. Infect. Dis..

[85] Colson P., Fournier P.-E., Delerce J., Million M., Bedotto M., Houhamdi L., Yahi N., Bayette J., Levasseur A., Fantini J. (2022). Culture and identification of a “Deltamicron” SARS-CoV-2 in a three cases cluster in southern France. J. Med. Virol..

[86] Simon-Loriere E., Montagutelli X., Lemoine F., Donati F., Touret F., Bourret J., Prot M., Munier S., Attia M., Conquet L. (2022). Rapid characterization of a Delta-Omicron SARS-CoV-2 recombinant detected in Europe. Biol. Sci..

[87] Sant’Anna F.H., Finger Andreis T., Salvato R.S., Muterle Varela A.P., Comerlato J., Gregianini T.S., Barcellos R.B., de Souza Godinho F.M., Resende P.C., da Luz Wallau G. (2023). Incipient Parallel Evolution of SARS-CoV-2 Deltacron Variant in South Brazil. Vaccines.

[88] Chavda V.P., Mishra T., Vuppu S. (2023). Immunological Studies to Understand Hybrid/Recombinant Variants of SARS-CoV-2. Vaccines.

[89] Chakraborty C., Bhattacharya M., Sharma A.R., Dhama K. (2022). Recombinant SARS-CoV-2 variants XD, XE, and XF: The emergence of recombinant variants requires an urgent call for research–Correspondence. Int. J. Surg..

[90] Thakur P., Thakur V., Kumar P., Singh Patel S.K. (2022). Emergence of novel omicron hybrid variants: BA(x), XE, XD, XF more than just alphabets. Int. J. Surg..

[91] Chavda V.P., Vuppu S., Mishra T., Balar P. (2023). The Emergence of Hybrid Variants of SARS-CoV-2: Towards Hybrid Immunity. Vaccines.

[92] Cinelli M., De Francisci Morales G., Galeazzi A., Quattrociocchi W., Starnini M. (2021). The echo chamber effect on social media. Proc. Natl. Acad. Sci. USA.

[93] Abbas J., Wang D., Su Z., Ziapour A. (2021). The Role of Social Media in the Advent of COVID-19 Pandemic: Crisis Management, Mental Health Challenges and Implications. Risk Manag. Healthc. Policy.

[94] Karbalaei M., Keikha M. (2022). Deltacron is a recombinant variant of SARS-CoV-2 but not a laboratory mistake. Ann. Med. Surg..

[95] Wang L., Gao G.F. (2022). The “Wolf” Is Indeed Coming: Recombinant “Deltacron” SARS-CoV-2 Detected. China CDC Wkly..

[96] Republic of Cyprus Ministry of Health New Coronavirus Disease (COVID-19). https://www.pio.gov.cy/coronavirus/eng/categories/important-announcements.

[97] Rahmani S., Rezaei N. (2022). Omicron (B.1.1.529) variant: Development, dissemination, and dominance. J. Med. Virol..

[98] Thakur V., Ratho R.K. (2022). OMICRON (B.1.1.529): A new SARS-CoV-2 variant of concern mounting worldwide fear. J. Med. Virol..

[99] Elliott P., Eales O., Steyn N., Tang D., Bodinier B., Wang H., Elliott J., Whitaker M., Atchison C., Diggle P.J. (2023). Twin peaks: The Omicron SARS-CoV-2 BA.1 and BA.2 epidemics in England. Science.

[100] Eales O., de Oliveira Martins L., Page A.J., Wang H., Bodinier B., Tang D., Haw D., Jonnerby J., Atchison C., Ashby D. (2022). Dynamics of competing SARS-CoV-2 variants during the Omicron epidemic in England. Nat. Commun..

[101] Agyapon-Ntra K., McSharry P.E. (2023). A global analysis of the effectiveness of policy responses to COVID-19. Sci. Rep..

[102] Rahimi F., Talebi Bezmin Abadi A. (2022). The Omicron subvariant BA.2: Birth of a new challenge during the COVID-19 pandemic. Int. J. Surg..

[103] Tiecco G., Storti S., Arsuffi S., Degli Antoni M., Focà E., Castelli F., Quiros-Roldan E. (2022). Omicron BA.2 Lineage, the “Stealth” Variant: Is It Truly a Silent Epidemic? A Literature Review. Int. J. Mol. Sci..

[104] Scandurra C., Bochicchio V., Dolce P., Valerio P., Muzii B., Maldonato N.M. (2023). Why people were less compliant with public health regulations during the second wave of the Covid-19 outbreak: The role of trust in governmental organizations, future anxiety, fatigue, and COVID-19 risk perception. Curr. Psychol..

[105] Zarowsky Z., Rashid T. (2023). Resilience and Wellbeing Strategies for Pandemic Fatigue in Times of COVID-19. Int. J. Appl. Posit. Psychol..

[106] Gavenčiak T., Monrad J.T., Leech G., Sharma M., Mindermann S., Bhatt S., Brauner J., Kulveit J. (2022). Seasonal variation in SARS-CoV-2 transmission in temperate climates: A Bayesian modelling study in 143 European regions. PLoS Comput. Biol..

[107] Cappi R., Casini L., Tosi D., Roccetti M. (2022). Questioning the seasonality of SARS-CoV-2: A Fourier spectral analysis. BMJ Open.

[108] Pascall D.J., Vink E., Blacow R., Bulteel N., Campbell A., Campbell R., Clifford S., Davis C., da Silva Filipe A., El Sakka N. (2023). Directions of change in intrinsic case severity across successive SARS-CoV-2 variant waves have been inconsistent. J. Infect..

[109] López-Andreo M.J., Vicente-Romero M.R., Bernal E., Navarro-González I., Salazar-Martínez F., Cánovas-Cánovas V., Gil-Ortuño C., Riquelme-Rocamora M.G., Solano F., Ibáñez-López F.J. (2023). Whole Sequencing and Detailed Analysis of SARS-CoV-2 Genomes in Southeast Spain: Identification of Recurrent Mutations in the 20E (EU1) Variant with Some Clinical Implications. Diseases.

[110] Islam M.R., Shahriar M., Bhuiyan M.A. (2022). The latest Omicron BA.4 and BA.5 lineages are frowning toward COVID-19 preventive measures: A threat to global public health. Heal. Sci. Rep..

[111] Desingu P.A., Nagarajan K. (2022). The emergence of Omicron lineages BA.4 and BA.5, and the global spreading trend. J. Med. Virol..

[112] Cyprus Statistical Service Tourism–Predefined Tables. https://www.cystat.gov.cy/en/KeyFiguresList?s=51&fbclid=IwAR0mThPdhjg-Uj64Q2kAW7ibreEZgx1i4PftJZ_orJimRXU1AOKpbUXExB0.

[113] Tabatabai M., Juarez P.D., Matthews-Juarez P., Wilus D.M., Ramesh A., Alcendor D.J., Tabatabai N., Singh K.P. (2023). An Analysis of COVID-19 Mortality during the Dominancy of Alpha, Delta, and Omicron in the USA. J. Prim. Care Community Health.

[114] Ward I.L., Bermingham C., Ayoubkhani D., Gethings O.J., Pouwels K.B., Yates T., Khunti K., Hippisley-Cox J., Banerjee A., Walker A.S. (2022). Risk of covid-19 related deaths for SARS-CoV-2 omicron (B.1.1.529) compared with delta (B.1.617.2): Retrospective cohort study. Br. Med. J..

[115] Mohapatra R.K., Tiwari R., Sarangi A.K., Islam M.R., Chakraborty C., Dhama K. (2022). Omicron (B.1.1.529) variant of SARS-CoV-2: Concerns, challenges, and recent updates. J. Med. Virol..

[116] Stowe J., Andrews N., Kirsebom F., Ramsay M., Bernal J.L. (2022). Effectiveness of COVID-19 vaccines against Omicron and Delta hospitalisation, a test negative case-control study. Nat. Commun..

[117] Chemaitelly H., Ayoub H.H., Coyle P., Tang P., Yassine H.M., Al-Khatib H.A., Smatti M.K., Hasan M.R., Al-Kanaani Z., Al-Kuwari E. (2022). Protection of Omicron sub-lineage infection against reinfection with another Omicron sub-lineage. Nat. Commun..

[118] Memorial Sloan Kettering Cancer Center Library SARS-CoV-2: It’s All “Omicron”. https://libguides.mskcc.org/SARS2/Omicron.

[119] Christoph J., Dorota K., Lennart K., Fabian Z., Timo J., Sparrer K.M., Frank K. (2022). Omicron: What Makes the Latest SARS-CoV-2 Variant of Concern So Concerning?. J. Virol..

[120] Ke H., Chang M.R., Marasco W.A. (2022). Immune Evasion of SARS-CoV-2 Omicron Subvariants. Vaccines.

[121] Mohapatra R.K., Verma S., Kandi V., Sarangi A.K., Seidel V., Das S.N., Behera A., Tuli H.S., Sharma A.K., Dhama K. (2023). The SARS-CoV-2 Omicron Variant and its Multiple Sub-lineages: Transmissibility, Vaccine Development, Antiviral Drugs, Monoclonal Antibodies, and Strategies for Infection Control–A Review. ChemistrySelect.

[122] Wang Q., Iketani S., Li Z., Liu L., Guo Y., Huang Y., Bowen A.D., Liu M., Wang M., Yu J. (2023). Alarming antibody evasion properties of rising SARS-CoV-2 BQ and XBB subvariants. Cell.

[123] Lenharo M. (2023). WHO declares end to COVID-19′s emergency phase. Nature.

[124] Gangavarapu K., Latif A.A., Mullen J.L., Alkuzweny M., Hufbauer E., Tsueng G., Haag E., Zeller M., Aceves C.M., Zaiets K. (2023). Outbreak.info genomic reports: Scalable and dynamic surveillance of SARS-CoV-2 variants and mutations. Nat. Methods.

