# Supplementary material for: Unraveling the Dynamics of Omicron (BA.1, BA.2, and BA.5) Waves and Emergence of the Deltacton Variant: Genomic Epidemiology of the SARS-CoV-2 Epidemic in Cyprus (Oct 2021–Oct 2022)
Source: Viruses. 2023 Sep 15;15(9):1933. doi: 10.3390/v15091933 (PMC10535466; doi:10.3390/v15091933)
Supplement: Supplementary file 1 [file viruses-15-01933-s001.zip › viruses-2614562-supplementary.pdf]

Table S1. Common S Protein Mutations of Delta and Omicron Lineages that were Identified in this Dataset[a].

Total Sequences[b]	Lineage[c]	Mutations and Number of Sequences Identified for Each Mutation										
47	B.1.617.2	T19R	G142D[d]	ΔE156	ΔF157	R158G	L452R	T478K	D614G	P681R	D950N	
		47	20	45	45	45	47	47	47	47	43	
2	AY.3	T19R	G142D	ΔE156	ΔF157	R158G	L452R	T478K	D614G	P681R	D950N	
		2	2	2	2	2	2	2	2	2	2	
106	AY.4	T19R	T95I	G142D[d]	ΔE156	ΔF157	R158G	L452R	T478K	D614G	P681R	D950N
		106	103	39	102	102	102	105	105	106	106	80
38	AY.4.2	T19R	T95I	G142D[d]	Y145H	ΔE156	ΔF157	R158G	A222V	L452R	T478K	D614G
		38	36	7	36	38	38	38	38	38	38	38
		P681R	D950N									
		38	34									
3	AY.4.2.1	T19R	V36F	T95I	Y145H	ΔE156	ΔF157	R158G	A222V	L452R	T478K	D614G
		3	3	3	3	3	3	3	3	3	3	3
		P681R	D950N									
		3	3									
1	AY.4.2.5	T19R	T95I	G142D	Y145H	ΔE156	ΔF157	R158G	A222V	G446V	L452R	T478K
		1	1	1	1	1	1	1	1	1	1	1
		D614G	P681R	D950N								
		1	1	1								
4	AY.4.5	T19R	T95I	G142D[d]	ΔE156	ΔF157	R158G	L452R	T478K	D614G	P681R	D950N
		4	4	1	4	4	4	4	4	4	4	3
		D1259Y										
		4										
1	AY.4.6	T19R	T95I	ΔE156	ΔF157	R158G	L452R	T478K	D614G	P681R	D950N	
		1	1	1	1	1	1	1	1	1	1	
3	AY.5	T19R	ΔE156	ΔF157	R158G	L452R	T478K	D614G	P681R	D950N[d]		
		3	3	3	3	3	3	3	3	2		

2	AY.6	T19R	G142D[d]	ΔE156	ΔF157	R158G	L452R	T478K	D614G	P681R	D950N	
		2	1	2	2	2	2	2	2	2	2	
22	AY.7.2	T19R	ΔE156	ΔF157	R158G	L452R	T478K	D614G	P681R	A688V[e]	D950N	
		22	19	19	19	22	22	22	22	17	20	
5	AY.9.2	T19R	ΔE156	ΔF157	R158G	A222V	L452R	T478K	D614G	P681R	D950N	
		5	5	5	5	5	5	5	5	5	5	
1	AY.23	T19R	ΔE156	ΔF157	R158G	L452R	T478K	D614G	P681R	D950N	V1264L	
		1	1	1	1	1	1	1	1	1	1	
1	AY.25	T19R	ΔE156	ΔF157	R158G	L452R	T478K	D614G	P681R	D950N		
		1	1	1	1	1	1	1	1	1		
1	AY.33	T19R	T29A	G142D	ΔE156	ΔF157	R158G	T250I	L452R	T478K	D614G	P681R
		1	1	1	1	1	1	1	1	1	1	1
1	AY.34	T19R	T95I	ΔE156	ΔF157	R158G	L452R	T478K	D614G	Q677H	P681R	D950N
		1	1	1	1	1	1	1	1	1	1	1
1	AY.34.1	T19R	T95I	ΔE156	ΔF157	R158G	L452R	T478K	D614G	Q677H	P681R	
		1	1	1	1	1	1	1	1	1	1	
4	AY.36	T19R	T95I	ΔE156	ΔF157	R158G	L452R	T478K	D614G	P681R	D950N[d]	V1104L
		4	4	4	4	4	4	4	4	4	2	4
1	AY.42	T19R	T95I	ΔE156	ΔF157	R158G	L452R	T478K	D614G	P681R		
		1	1	1	1	1	1	1	1	1		
197	AY.43	T19R	ΔE156	ΔF157	R158G	L452R	T478K	D614G	P681R	D950N		
		197	192	192	192	197	197	197	197	150		
1	AY.43.8	T19R	G142D	ΔE156	ΔF157	R158G	L452R	T478K	D614G	P681R		
		1	1	1	1	1	1	1	1	1		
2	AY.46.5	T19R	ΔE156	ΔF157	R158G	L452R	T478K	D614G	P681R			
		2	2	2	2	2	2	2	2			
4	AY.46.6	T19R	ΔE156	ΔF157	R158G	L452R	T478K	D614G	P681R	D950N[d]		
		4	4	4	4	4	4	4	4	1		
2	AY.60	T19R	ΔE156	ΔF157	R158G	A222V	L452R	T478K	D614G	P681R	D950N	
		2	2	2	2	2	2	2	2	2	2	
32	AY.98	T19R	G142D[d]	ΔE156	ΔF157	R158G	L452R	T478K	D614G	P681R	D950N	

		32	15	31	31	31	32	32	32	32	29	
11	AY.98.1	T19R	ΔE156	ΔF157	R158G	P251L	L452R	T478K	D614G	P681R	D950N	
		11	11	11	11	11	11	11	11	11	11	
4	AY.103	T19R	G142D[d]	ΔE156	ΔF157	R158G	L452R	T478K	D614G	P681R	D950N	
		4	1	4	4	4	4	4	4	4	4	
1	AY.106	T19R	T95I	ΔE156	ΔF157	R158G	S255F	L452R	T478K	D614G	P681R	
		1	1	1	1	1	1	1	1	1	1	
1	AY.107	T19R	T95I	ΔE156	ΔF157	R158G	L452R	T478K	D614G	P681R	D950N	H1101Y
		1	1	1	1	1	1	1	1	1	1	1
1	AY.109	T19R	ΔE156	ΔF157	R158G	L452R	T478K	D614G	P681R	D950N		
		1	1	1	1	1	1	1	1	1		
1	AY.111	T19R	T95I	ΔE156	ΔF157	R158G	L452R	T478K	D614G	P681R	D950N	
		1	1	1	1	1	1	1	1	1	1	
1	AY.114	T19R	T95I	ΔE156	ΔF157	R158G	L452R	T478K	D614G	P681R	D950N	
		1	1	1	1	1	1	1	1	1	1	
1	AY.116	T19R	T95I	ΔE156	ΔF157	R158G	L452R	T478K	D614G	P681R	D950N	
		1	1	1	1	1	1	1	1	1	1	
1	AY.119.2	T19R	T95I	G142D	ΔE156	ΔF157	R158G	L452R	T478K	D614G	Q675H	P681R
		1	1	1	1	1	1	1	1	1	1	1
2	AY.120	T19R	T95I[d]	ΔE156	ΔF157	R158G	L452R	T478K	D614G	P681R	D950N	
		2	1	2	2	2	2	2	2	2	2	
1	AY.120.2.1	T19R	H66Y	T95I	G142D	ΔE156	ΔF157	R158G	L452R	T478K	D614G	P681R
		1	1	1	1	1	1	1	1	1	1	1
		D950N										
		1										
45	AY.121	T19R	T95I	ΔE156	ΔF157	R158G	L452R	T478K	D614G	P681R	D950N	
		45	44	45	45	45	44	45	45	45	39	
115	AY.122	T19R	ΔE156	ΔF157	R158G	L452R	T478K	D614G	P681R	D950N		
		115	112	112	112	115	115	115	115	89		
3	AY.124	T19R	T95I	ΔE156	ΔF157	R158G	L452R	T478K	D614G	P681R	D950N	
		3	3	3	3	3	3	3	3	3	3	

1	AY.124.1	T19R	T95I	ΔE156	ΔF157	R158G	L452R	T478K	D614G	P681R	D950N	
		1	1	1	1	1	1	1	1	1	1	
3	AY.125	T19R	T95I	ΔE156	ΔF157	R158G	L452R	T478K	D614G	P681R	D950N[d]	
		3	3	3	3	3	3	3	3	3	2	
18	AY.126	T19R	T95I	ΔE156	ΔF157	R158G	L452R	T478K	D614G	P681R	I850L	D950N
		18	14	17	17	17	18	18	18	18	17	16
44	AY.127	T19R	T95I	G142D[d]	ΔE156	ΔF157	R158G	L452R	T478K	D614G	P681R	D950N
		44	41	10	44	44	44	44	44	44	44	42
1	AY.127.2	T19R	ΔE156	ΔF157	R158G	L452R	T478K	D614G	P681R	T859N	D950N	
		1	1	1	1	1	1	1	1	1	1	
2	AY.128	T19R	T95I	G142D	ΔE156	ΔF157	R158G	L452R	T478K	D614G	P681R	D950N[d]
		2	2	2	2	2	2	2	2	2	2	1
1	AY.129	T19R	T95I	ΔE156	ΔF157	R158G	L452R	T478K	D614G	P681R	D950N	
		1	1	1	1	1	1	1	1	1	1	
337	BA.1	A67V	ΔH69	ΔV70	T95I	ΔG142	ΔV143	ΔY144	Y145D	ΔN211	L212I	ins214EPE
		336	336	336	337	337	337	337	337	319	319	304
		G339D	S371L	S373P	S375F	K417N	N440K	G446S	S477N	T478K	E484A	Q493R
		335	323	325	326	310	316	331	327	328	326	331
		G496S	Q498R	N501Y	Y505H	T547K	D614G	H655Y	N679K	P681H	N764K	D796Y
		331	330	330	330	336	337	337	337	337	336	337
		N856K	Q954H	N969K	L981F							
		337	337	337	337							
529	BA.1.1	A67V	ΔH69	ΔV70	T95I	ΔG142	ΔV143	ΔY144	Y145D	ΔN211	L212I	ins214EPE
		529	529	529	529	529	529	528	529	522	522	512
		G339D	R346K	S371L	S373P	S375F	K417N	N440K	G446S	S477N	T478K	E484A
		528	528	523	524	524	518	527	525	525	525	524
		Q493R	G496S	Q498R	N501Y	Y505H	T547K	D614G	H655Y	N679K	P681H	N764K
		525	525	525	525	526	529	529	529	529	529	529
		D796Y	N856K	Q954H	N969K	L981F						
		529	528	529	529	529						
71	BA.1.1.1	A67V	ΔH69	ΔV70	T95I	ΔG142	ΔV143	ΔY144	Y145D	ΔN211	L212I	ins214EPE

		71	71	71	71	71	71	71	71	70	70	65
		G339D	R346K	S371L	S373P	S375F	K417N	N440K	G446S	S477N	T478K	E484A
		71	71	69	69	69	69	70	71	68	68	69
		Q493R	G496S	Q498R	N501Y	Y505H	T547K	D614G	H655Y	N679K	P681H	N764K
		70	70	70	69	69	71	71	71	71	71	71
		D796Y	N856K	Q954H	N969K	L981F						
		71	71	71	71	71						
2	BA.1.1.11	A67V	ΔH69	ΔV70	T95I	ΔG142	ΔV143	ΔY144	Y145D	ΔN211	L212I	ins214EPE
		2	2	2	2	2	2	2	2	2	2	2
		G339D	R346K	S371L	S373P	S375F	K417N[d]	N440K	G446S	S477N	T478K	E484A
		2	2	2	2	2	1	2	2	2	2	2
		Q493R	G496S	Q498R	N501Y	Y505H	T547K	D614G	H655Y	N679K	P681H	N764K
		2	2	2	2	2	2	2	2	2	2	2
		D796Y	N856K	Q954H	N969K	L981F						
		2	2	2	2	2						
1	BA.1.1.14	A67V	ΔH69	ΔV70	T95I	ΔG142	ΔV143	ΔY144	Y145D	ΔN211	L212I	ins214EPE
		1	1	1	1	1	1	1	1	1	1	1
		G339D	R346K	S371L	S373P	S375F	K417N	N440K	G446S	S477N	T478K	E484A
		1	1	1	1	1	1	1	1	1	1	1
		Q493R	G496S	Q498R	N501Y	Y505H	T547K	D614G	H655Y	N679K	P681H	N764K
		1	1	1	1	1	1	1	1	1	1	1
		D796Y	N856K	Q954H	N969K	L981F						
		1	1	1	1	1						
157	BA.1.1.15	A67V	ΔH69	ΔV70	T95I	ΔG142	ΔV143	ΔY144	Y145D	ΔN211	L212I	ins214EPE
		157	157	157	157	157	157	157	157	154	154	151
		G339D	R346K	S371L	S373P	S375F	K417N	N440K	G446S	S477N	T478K	E484A
		157	157	156	156	156	155	155	156	155	155	157
		Q493R	G496S	Q498R	N501Y	Y505H	T547K	D614G	H655Y	N679K	P681H	N764K
		156	156	156	156	156	157	157	157	157	157	157
		D796Y	N856K	Q954H	N969K	L981F						
		157	157	157	157	157						

2	BA.1.1.18	A67V	ΔH69	ΔV70	T95I	ΔG142	ΔV143	ΔY144	Y145D	ΔN211	L212I	ins214EPE
		2	2	2	2	2	2	2	2	2	2	2
		G339D	R346K	S371L	S373P	S375F	K417N	N440K	G446S	S477N	T478K	E484A
		2	2	2	2	2	2	2	2	2	2	2
		Q493R	G496S	Q498R	N501Y	Y505H	T547K	D614G	H655Y	N679K	P681H	N764K
		2	2	2	2	2	2	2	2	2	2	2
		D796Y	N856K	Q954H	N969K	L981F						
		2	2	2	2	2						
1	BA.1.7	A67V	ΔH69	ΔV70	T95I	ΔG142	ΔV143	ΔY144	Y145D	ΔN211	L212I	ins214EPE
		1	1	1	1	1	1	1	1	1	1	1
		G339D	S371L	S373P	S375F	K417N	N440K	G446S	S477N	T478K	E484A	Q493R
		1	1	1	1	1	1	1	1	1	1	1
		G496S	Q498R	N501Y	Y505H	T547K	D614G	H655Y	N679K	P681H	N764K	D796Y
		1	1	1	1	1	1	1	1	1	1	1
		N856K	Q954H	N969K	L981F							
		1	1	1	1							
1	BA.1.8	A67V	ΔH69	ΔV70	T95I	ΔG142	ΔV143	ΔY144	Y145D	ΔN211	L212I	ins214EPE
		1	1	1	1	1	1	1	1	1	1	1
		G339D	S371L	S373P	S375F	K417N	N440K	G446S	S477N	T478K	E484A	Q493R
		1	1	1	1	1	1	1	1	1	1	1
		G496S	Q498R	N501Y	Y505H	T547K	D614G	H655Y	N679K	P681H	N764K	D796Y
		1	1	1	1	1	1	1	1	1	1	1
		P809S	N856K	Q954H	N969K	L981F						
		1	1	1	1	1						
1	BA.1.10	A67V	ΔH69	ΔV70	T95I	ΔG142	ΔV143	ΔY144	Y145D	ΔN211	L212I	ins214EPE
		1	1	1	1	1	1	1	1	1	1	1
		G339D	S371L	S373P	S375F	K417N	G446S	S477N	T478K	E484A	Q493R	G496S
		1	1	1	1	1	1	1	1	1	1	1
		Q498R	N501Y	Y505H	T547K	D614G	H655Y	N679K	P681H	N764K	D796Y	N856K
		1	1	1	1	1	1	1	1	1	1	1
		Q954H	N969K	L981F								

		1	1	1								
2	BA.1.13	A67V	ΔH69	ΔV70	T95I	ΔG142	ΔV143	ΔY144	Y145D	ΔN211	L212I	ins214EPE
		2	2	2	2	2	2	2	2	2	2	2
		G339D	S371L	S373P	S375F	K417N	N440K	G446S	S477N	T478K	E484A	Q493R
		2	2	2	2	2	2	2	2	2	2	2
		G496S	Q498R	N501Y	Y505H	T547K	D614G	H655Y	N679K	P681H	N764K	D796Y
		2	2	2	2	2	2	2	2	2	2	2
		N856K	Q954H	N969K	L981F							
		2	2	2	2							
25	BA.1.15	A67V	ΔH69	ΔV70	T95I	ΔG142	ΔV143	ΔY144	Y145D	ΔN211	L212I	ins214EPE
		25	25	25	25	25	25	25	25	22	22	22
		G339D	S371L	S373P	S375F	K417N	N440K	G446S	S477N	T478K	E484A	Q493R
		25	23	23	23	19	24	24	23	23	24	24
		G496S	Q498R	N501Y	Y505H	T547K	D614G	H655Y	N679K	P681H	N764K	D796Y
		24	24	24	24	25	25	25	25	25	25	25
		N856K	Q954H	N969K	L981F							
		25	25	25	25							
14	BA.1.15.1	A67V	ΔH69	ΔV70	T95I	ΔG142	ΔV143	ΔY144	Y145D	ΔN211	L212I	ins214EPE
		14	14	14	14	14	14	14	14	12	12	12
		G339D	S371L	S373P	S375F	K417N[d]	N440K	G446S	S477N	T478K	E484A	Q493R
		14	13	13	13	8	14	14	13	13	14	14
		G496S	Q498R	N501Y	Y505H	T547K	D614G	H655Y	N679K	P681H	N764K	D796Y
		13	13	13	13	14	14	14	14	14	14	14
		N856K	Q954H	N969K	L981F	I1081V						
		14	14	14	14	14						
16	BA.1.16	A67V	ΔH69	ΔV70	T95I	ΔG142	ΔV143	ΔY144	Y145D	ΔN211	L212I	ins214EPE
		16	16	16	16	16	16	16	16	12	12	12
		G339D	S371L	S373P	S375F	K417N	N440K	G446S	S477N	T478K	E484A	Q493R
		16	15	15	15	14	16	16	16	16	15	16
		G496S	Q498R	N501Y	Y505H	T547K	D614G	H655Y	N679K	P681H	N764K	D796Y
		16	16	16	16	16	16	16	16	16	16	16

		N856K	Q954H	N969K	L981F							
		16	16	16	16							
99	BA.1.17	A67V	ΔH69	ΔV70	T95I	ΔG142	ΔV143	ΔY144	Y145D	ΔN211	L212I	ins214EPE
		99	99	99	98	99	99	99	99	92	92	87
		G339D	S371L	S373P	S375F	K417N	N440K	G446S	S477N	T478K	E484A	Q493R
		96	94	94	94	92	97	98	98	98	97	96
		G496S	Q498R	N501Y	Y505H	T547K	D614G	H655Y	N679K	P681H	N764K	D796Y
		97	98	97	97	99	98	98	99	99	99	99
		N856K	Q954H	N969K	L981F							
		99	99	99	99							
194	BA.1.17.2	A67V	ΔH69	ΔV70	T95I	ΔG142	ΔV143	ΔY144	Y145D	ΔN211	L212I	ins214EPE
		194	194	194	194	194	194	194	194	186	186	180
		G339D	S371L	S373P	S375F	K417N	N440K	G446S	S477N	T478K	E484A	Q493R
		193	192	193	193	179	186	190	192	192	192	193
		G496S	Q498R	N501Y	Y505H	T547K	D614G	H655Y	N679K	P681H	A701V	N764K
		191	191	191	190	194	194	194	194	194	194	194
		D796Y	N856K	Q954H	N969K	L981F						
		194	194	194	194	194						
14	BA.1.18	A67V	ΔH69	ΔV70	T95I	ΔG142	ΔV143	ΔY144	Y145D	ΔN211	L212I	ins214EPE
		14	14	14	14	14	14	14	14	13	13	13
		G339D	S371L	S373P	S375F	K417N	N440K	G446S	S477N	T478K	E484A	Q493R
		14	13	13	13	12	12	13	13	13	13	13
		G496S	Q498R	N501Y	Y505H	T547K	D614G	H655Y	N679K	P681H	N764K	D796Y
		13	13	13	13	13	14	14	14	14	14	14
		N856K	Q954H	N969K	L981F							
		14	14	14	14							
2	BA.1.19	A67V	ΔH69	ΔV70	T95I	ΔG142	ΔV143	ΔY144	Y145D	ΔN211[d]	L212I[d]	ins214EPE[d]
		2	2	2	2	2	2	2	2	1	1	1
		G339D	S371L[d]	S373P[d]	S375F[d]	K417N	N440K[d]	G446S[d]	S477N[d]	T478K[d]	E484A[d]	Q493R[d]
		2	1	1	1	2	1	1	1	1	1	1
		G496S[d]	Q498R[d]	N501Y[d]	Y505H[d]	T547K	D614G	H655Y	N679K	P681H	N764K	D796Y

		1	1	1	1	2	2	2	2	2	2	2
		N856K	Q954H	N969K	L981F							
		2	2	2	2							
2	BA.1.20	A67V	ΔH69	ΔV70	T95I	ΔG142	ΔV143	ΔY144	Y145D	ΔN211	L212I	ins214EPE
		2	2	2	2	2	2	2	2	2	2	2
		G339D	S371L	S373P	S375F	K417N	N440K	G446S	S477N	T478K	E484A	Q493R
		2	2	2	2	2	2	2	2	2	2	2
		G496S	Q498R	N501Y	Y505H	T547K	D614G	H655Y	N679K	P681H	N764K	D796Y
		2	2	2	2	2	2	2	2	2	2	2
		N856K	Q954H	N969K	L981F							
		2	2	2	2							
1	BA.1.21	A67V	ΔH69	ΔV70	T95I	ΔG142	ΔV143	ΔY144	Y145D	ΔN211	L212I	G339D
		1	1	1	1	1	1	1	1	1	1	1
		S371L	S373P	S375F	K417N	N440K	G446S	S477N	T478K	E484A	Q493R	G496S
		1	1	1	1	1	1	1	1	1	1	1
		Q498R	N501Y	Y505H	T547K	D614G	H655Y	N679K	P681H	N764K	D796Y	N856K
		1	1	1	1	1	1	1	1	1	1	1
		Q954H	N969K	L981F								
		1	1	1								
1185	BA.2	T19I	ΔL24	ΔP25	ΔP26	A27S	G142D	V213G	G339D	S371F	S373P	S375F
		1184	1185	1185	1185	1185	1184	1184	1181	1178	1182	1182
		T376A	D405N	R408S	K417N	N440K	S477N	T478K	E484A	Q493R	Q498R	N501Y
		1179	1185	1185	1185	1184	1185	1185	1185	1185	1185	1185
		Y505H	D614G	H655Y	N679K	P681H	N764K	D796Y	Q954H	N969K		
		1185	1185	1185	1184	1185	1185	1185	1185	1185		
9	BA.2.1	T19I	ΔL24	ΔP25	ΔP26	A27S	G142D	V213G	G339D	S371F	S373P	S375F
		9	9	9	9	9	9	9	9	9	9	9
		T376A	D405N	R408S	K417N	N440K	S477N	T478K	E484A	Q493R	Q498R	N501Y
		9	9	9	9	9	9	9	9	9	9	9
		Y505H	D614G	H655Y	N679K	P681H	N764K	D796Y	Q954H	N969K		
		9	9	9	9	9	9	9	9	9		

1	BA.2.2	T19I	ΔL24	ΔP25	ΔP26	A27S	G142D	V213G	G339D	S371F	S373P	S375F
		1	1	1	1	1	1	1	1	1	1	1
		T376A	D405N	R408S	K417N	N440K	S477N	T478K	E484A	Q493R	Q498R	N501Y
		1	1	1	1	1	1	1	1	1	1	1
		Y505H	D614G	H655Y	N679K	P681H	N764K	D796Y	Q954H	N969K		
		1	1	1	1	1	1	1	1	1		
49	BA.2.3	T19I	ΔL24	ΔP25	ΔP26	A27S	G142D	V213G	G339D	S371F	S373P	S375F
		49	49	49	49	49	49	49	49	49	49	49
		T376A	D405N	R408S	K417N	N440K	S477N	T478K	E484A	Q493R	Q498R	N501Y
		49	49	49	49	49	49	49	49	49	49	49
		Y505H	D614G	H655Y	N679K	P681H	N764K	D796Y	Q954H	N969K		
		49	49	49	49	49	49	49	49	49		
1	BA.2.3.15	T19I	ΔL24	ΔP25	ΔP26	A27S	G142D	V213G	D215E	G339D	S371F	S373P
		1	1	1	1	1	1	1	1	1	1	1
		S375F	T376A	D405N	R408S	K417N	N440K	S477N	T478K	E484A	Q493R	Q498R
		1	1	1	1	1	1	1	1	1	1	1
		N501Y	Y505H	D614G	H655Y	N679K	P681H	N764K	D796Y	Q954H	N969K	
		1	1	1	1	1	1	1	1	1	1	
6	BA.2.3.20	T19I	ΔL24	ΔP25	ΔP26	A27S	G142D	M153T	N164K	V213G	H245N	G257D
		6	6	6	6	6	6	6	6	6	6	6
		G339D	S371F	S373P	S375F	T376A	D405N	R408S	K417N	N440K	K444R	N450D
		6	6	6	6	6	6	6	6	6	6	6
		L452M	N460K	S477N	T478K	E484R	Q498R	N501Y	Y505H	D614G	H655Y	N679K
		6	6	6	6	6	6	6	6	6	6	6
		P681H	N764K	D796Y	Q954H	N969K						
		6	6	6	6	6						
5	BA.2.5	T19I	ΔL24	ΔP25	ΔP26	A27S	G142D	V213G	G339D	S371F	S373P	S375F
		5	5	5	5	5	5	5	5	5	5	5
		T376A	D405N	R408S	K417N	N440K	S477N	T478K	E484A	Q493R	Q498R	N501Y
		5	5	5	5	5	5	5	5	5	5	5
		Y505H	D614G	H655Y	N679K	P681H	N764K	D796Y	Q954H	N969K		

		5	5	5	5	5	5	5	5	5		
128	BA.2.9	T19I	ΔL24	ΔP25	ΔP26	A27S	G142D	V213G	G339D	S371F	S373P	S375F
		128	128	128	128	128	128	128	125	122	126	126
		T376A	D405N	R408S	K417N	N440K	S477N	T478K	E484A	Q493R	Q498R	N501Y
		125	128	128	128	128	128	128	128	128	128	128
		Y505H	D614G	H655Y	N679K	P681H	N764K	D796Y	Q954H	N969K		
		128	128	128	128	128	128	128	128	128		
2	BA.2.9.3	T19I	ΔL24	ΔP25	ΔP26	A27S	W64R	G142D	V213G	G339D	S371F	S373P
		2	2	2	2	2	2	2	2	2	2	2
		S375F	T376A	D405N	R408S	K417N	N440K	S477N	T478K	E484A	Q493R	Q498R
		2	2	2	2	2	2	2	2	2	2	2
		N501Y	Y505H	D614G	H655Y	N679K	P681H	N764K	D796Y	Q954H	N969K	
		2	2	2	2	2	2	2	2	2	2	
2	BA.2.9.5	T19I	ΔL24	ΔP25	ΔP26	A27S	G142D	V213G	G339D	S371F[d]	S373P	S375F[d]
		2	2	2	2	2	2	2	2	1	2	1
		T376A[d]	D405N	R408S	K417N	N440K	S477N	T478K	E484A	Q493R	Q498R	N501Y
		1	2	2	2	2	2	2	2	2	2	2
		Y505H	D614G	H655Y	N679K	P681H	N764K	D796Y	Q954H	N969K		
		2	2	2	2	2	2	2	2	2		
8	BA.2.10	T19I	ΔL24	ΔP25	ΔP26	A27S	G142D	V213G	G339D	S371F	S373P	S375F
		8	8	8	8	8	8	8	8	8	8	8
		T376A	D405N	R408S	K417N	N440K	S477N	T478K	E484A	Q493R	Q498R	N501Y
		8	8	8	8	8	8	8	8	8	8	8
		Y505H	D614G	H655Y	N679K	P681H	N764K	D796Y	Q954H	N969K		
		8	8	8	8	8	8	8	8	8		
1	BA.2.10.3	T19I	ΔL24	ΔP25	ΔP26	A27S	I68T	G142D	V213G	G339D	S371F	S373P
		1	1	1	1	1	1	1	1	1	1	1
		S375F	T376A	D405N	R408S	K417N	N440K	S477N	T478K	E484A	Q493R	Q498R
		1	1	1	1	1	1	1	1	1	1	1
		N501Y	Y505H	D614G	H655Y	N679K	P681H	N764K	D796Y	Q954H	N969K	
		1	1	1	1	1	1	1	1	1	1	

47	BA.2.12.1	T19I	ΔL24	ΔP25	ΔP26	A27S	G142D	V213G	G339D	S371F	S373P	S375F
		47	47	47	47	47	47	47	47	47	47	47
		T376A	D405N	R408S	K417N	N440K	L452Q	S477N	T478K	E484A	Q493R	Q498R
		47	47	47	47	47	47	47	47	47	47	47
		N501Y	Y505H	D614G	H655Y	N679K	P681H	S704L	N764K	D796Y	Q954H	N969K
		47	47	47	47	47	47	47	47	47	47	47
3	BA.2.18	T19I	ΔL24	ΔP25	ΔP26	A27S	G142D	V213G	G339D	S371F	S373P	S375F
		3	3	3	3	3	3	3	3	3	3	3
		T376A	D405N	R408S	K417T	N440K	S477N	T478K	E484A	Q493R	Q498R	N501Y
		3	3	3	3	3	3	3	3	3	3	3
		Y505H	D614G	H655Y	N679K	P681H	N764K	D796Y	Q954H	N969K		
		3	3	3	3	3	3	3	3	3		
2	BA.2.19	T19I	ΔL24	ΔP25	ΔP26	A27S	G142D	V213G	G339D	S371F	S373P	S375F
		2	2	2	2	2	2	2	2	2	2	2
		T376A	D405N	R408S	K417N	N440K	S477N	T478K	E484A	Q493R	Q498R	N501Y
		2	2	2	2	2	2	2	2	2	2	2
		Y505H	D614G	H655Y	N679K	P681H	N764K	D796Y	Q954H	N969K		
		2	2	2	2	2	2	2	2	2		
1	BA.2.23	T19I	ΔL24	ΔP25	ΔP26	A27S	G142D	V213G	G339D	S371F	S373P	S375F
		1	1	1	1	1	1	1	1	1	1	1
		T376A	D405N	R408S	K417N	N440K	S477N	T478K	E484A	Q493R	Q498R	N501Y
		1	1	1	1	1	1	1	1	1	1	1
		Y505H	D614G	H655Y	N679K	P681H	N764K	D796Y	Q954H	N969K		
		1	1	1	1	1	1	1	1	1		
1	BA.2.23.1	T19I	ΔL24	ΔP25	ΔP26	A27S	G142D	V213G	G339D	S371F	S373P	S375F
		1	1	1	1	1	1	1	1	1	1	1
		T376A	D405N	R408S	K417N	N440K	S477N	T478K	E484A	Q493R	Q498R	N501Y
		1	1	1	1	1	1	1	1	1	1	1
		Y505H	D614G	H655Y	N679K	P681H	N764K	D796Y	Q954H	N969K		
		1	1	1	1	1	1	1	1	1		
1	BA.2.31	T19I	ΔL24	ΔP25	ΔP26	A27S	G142D	V213G	G339D	S371F	S373P	S375F

		1	1	1	1	1	1	1	1	1	1	1
		T376A	D405N	R408S	K417N	N440K	S477N	T478K	E484A	Q493R	Q498R	N501Y
		1	1	1	1	1	1	1	1	1	1	1
		Y505H	D614G	H655Y	N679K	P681H	N764K	D796Y	Q954H	N969K		
		1	1	1	1	1	1	1	1	1		
3	BA.2.36	T19I	ΔL24	ΔP25	ΔP26	A27S	I68T	G142D	V213G	G339D	S371F	S373P
		3	3	3	3	3	3	3	3	3	3	3
		S375F	T376A	D405N	R408S	K417N	N440K	S477N	T478K	E484A	Q493R	Q498R
		3	3	3	3	3	3	3	3	3	3	3
		N501Y	Y505H	D614G	H655Y	N679K	P681H	N764K	D796Y	Q954H	N969K	
		3	3	3	3	3	3	3	3	3	3	
45	BA.2.37	T19I	ΔL24	ΔP25	ΔP26	A27S	G142D	V213G	G339D	S371F	S373P	S375F
		45	45	45	45	45	45	45	45	45	45	45
		T376A	D405N	R408S	K417N	N440K	S477N	T478K	E484A	Q493R	Q498R	N501Y
		44	45	45	45	45	45	45	45	45	45	45
		Y505H	D614G	H655Y	N679K	P681H	N764K	D796Y	Q954H	N969K		
		45	45	45	45	45	45	45	45	45		
2	BA.2.38	T19I	ΔL24	ΔP25	ΔP26	A27S	G142D	V213G	G339D	S371F	S373P	S375F
		2	2	2	2	2	2	2	2	2	2	2
		T376A	D405N	R408S	K417T	N440K	S477N	T478K	E484A	Q493R	Q498R	N501Y
		2	2	2	2	2	2	2	2	2	2	2
		Y505H	D614G	H655Y	N679K	P681H	N764K	D796Y	Q954H	N969K		
		2	2	2	2	2	2	2	2	2		
1	BA.2.39	T19I	ΔL24	ΔP25	ΔP26	A27S	G142D	V213G	G339D	S371F	S373P	S375F
		1	1	1	1	1	1	1	1	1	1	1
		T376A	D405N	R408S	K417N	N440K	S477N	T478K	E484A	Q493R	Q498R	N501Y
		1	1	1	1	1	1	1	1	1	1	1
		Y505H	D614G	H655Y	N679K	P681H	N764K	D796Y	L858I	Q954H	N969K	
		1	1	1	1	1	1	1	1	1	1	
1	BA.2.41	T19I	ΔL24	ΔP25	ΔP26	A27S	G142D	V213G	G339D	S371F	S373P	S375F
		1	1	1	1	1	1	1	1	1	1	1

		T376A	D405N	R408S	K417N	N440K	S477N	T478K	E484A	Q493R	Q498R	N501Y
		1	1	1	1	1	1	1	1	1	1	1
		Y505H	D614G	H655Y	N679K	P681H	N764K	D796Y	Q954H	N969K	T1231S	
		1	1	1	1	1	1	1	1	1	1	
1	BA.2.56	T19I	ΔL24	ΔP25	ΔP26	A27S	G142D	V213G	G339D	S371F	S373P	S375F
		1	1	1	1	1	1	1	1	1	1	1
		T376A	D405N	R408S	K417N	N440K	L452M	S477N	T478K	E484A	Q493R	Q498R
		1	1	1	1	1	1	1	1	1	1	1
		N501Y	Y505H	D614G	H655Y	N679K	P681H	N764K	D796Y	Q954H	N969K	
		1	1	1	1	1	1	1	1	1	1	
3	BA.2.63	T19I	ΔL24	ΔP25	ΔP26	A27S	G142D	V213G	G339D	S371F	S373P	S375F
		3	3	3	3	3	3	3	3	3	3	3
		T376A	D405N	R408S	K417N	N440K	S477N	T478K	E484A	Q493R	Q498R	N501Y
		3	3	3	3	3	3	3	3	3	3	3
		Y505H	D614G	H655Y	N679K	P681H	N764K	D796Y	Q954H	N969K		
		3	3	3	3	3	3	3	3	3		
6	BA.2.65	T19I	ΔL24	ΔP25	ΔP26	A27S	G142D	V213G	G339D	S371F	S373P	S375F
		6	6	6	6	6	6	6	6	5	5	5
		T376A	D405N	R408S	K417N	N440K	S477N	T478K	E484A	Q493R	Q498R	N501Y
		5	6	6	6	6	6	6	6	6	6	6
		Y505H	D614G	H655Y	N679K	P681H	N764K	D796Y	Q954H	N969K		
		6	6	6	6	6	6	6	6	6		
1	BA.2.68	T19I	ΔL24	ΔP25	ΔP26	A27S	G142D	V213G	G339D	S371F	S373P	S375F
		1	1	1	1	1	1	1	1	1	1	1
		T376A	D405N	R408S	K417N	N440K	S477N	T478K	E484A	Q493R	Q498R	N501Y
		1	1	1	1	1	1	1	1	1	1	1
		Y505H	D614G	H655Y	N679K	P681H	N764K	D796Y	Q954H	N969K		
		1	1	1	1	1	1	1	1	1		
4	BA.2.71	T19I	ΔL24	ΔP25	ΔP26	A27S	W64R	G142D	V213G	G339D	S371F	S373P
		4	4	4	4	4	4	4	4	4	4	4
		S375F	T376A	D405N	R408S	K417N	N440K	S477N	T478K	E484A	Q493R	Q498R

		4	4	4	4	4	4	4	4	4	4	4
		N501Y	Y505H	D614G	H655Y	N679K	P681H	N764K	D796Y	Q954H	N969K	
		4	4	4	4	4	4	4	4	4	4	
1	BA.2.72	T19I	ΔL24	ΔP25	ΔP26	A27S	G142D	L212S	V213G	G339D	S371F	S373P
		1	1	1	1	1	1	1	1	1	1	1
		S375F	T376A	D405N	R408S	K417N	N440K	S477N	T478K	E484A	Q493R	Q498R
		1	1	1	1	1	1	1	1	1	1	1
		N501Y	Y505H	D614G	H655Y	N679K	P681H	N764K	D796Y	Q954H	N969K	
		1	1	1	1	1	1	1	1	1	1	
1	BN.1.3	T19I	ΔL24	ΔP25	ΔP26	A27S	G142D	K147E	W152R	F157L	I210V	V213G
		1	1	1	1	1	1	1	1	1	1	1
		G257S	G339H	R346T	K356T	S371F	S373P	S375F	T376A	D405N	R408S	K417N
		1	1	1	1	1	1	1	1	1	1	1
		N440K	G446S	N460K	S477N	T478K	E484A	F490S	Q498R	N501Y	Y505H	D614G
		1	1	1	1	1	1	1	1	1	1	1
		H655Y	N679K	P681H	N764K	D796Y	Q954H	N969K				
		1	1	1	1	1	1	1				
2	XBB.1	T19I	ΔL24	ΔP25	ΔP26	V83A	G142D	ΔY144	H146Q	Q183E	V213E	G252V
		2	2	2	2	2	2	2	2	2	2	2
		G339H	R346T	L368I	S371F	S373P	S375F	T376A	D405N	R408S	K417N	N440K
		2	2	2	2	2	2	2	2	2	2	2
		V445P	G446S	N460K	S477N	T478K	E484A	F486S	F490S	Q498R	N501Y	Y505H
		2	2	2	2	2	2	2	2	2	2	2
		D614G	H655Y	N679K	P681H	N764K	D796Y	Q954H	N969K			
		2	2	2	2	2	2	2	2			
1	XBB.2	T19I	ΔL24	ΔP25	ΔP26	V83A	G142D	ΔY144	H146Q	Q183E	V213E	D253G
		1	1	1	1	1	1	1	1	1	1	1
		G339H	R346T	L368I	S371F	S373P	S375F	T376A	D405N	R408S	K417N	N440K
		1	1	1	1	1	1	1	1	1	1	1
		V445P	G446S	N460K	S477N	T478K	E484A	F486S	F490S	Q498R	N501Y	Y505H
		1	1	1	1	1	1	1	1	1	1	1

		D614G	H655Y	N679K	P681H	N764K	D796Y	Q954H	N969K			
		1	1	1	1	1	1	1	1			
1	XL	T19I	ΔL24	ΔP25	ΔP26	A27S	G142D	V213G	G339D	S371F	S373P	S375F
		1	1	1	1	1	1	1	1	1	1	1
		T376A	D405N	R408S	K417N	N440K	S477N	T478K	E484A	Q493R	Q498R	N501Y
		1	1	1	1	1	1	1	1	1	1	1
		Y505H	D614G	H655Y	N679K	P681H	N764K	D796Y	Q954H	N969K		
		1	1	1	1	1	1	1	1	1		
3	XAL	L8F	T19I	ΔL24	ΔP25	ΔP26	A27S	G142D	V213G	G339D	S371F	S373P
		3	3	3	3	3	3	3	3	3	3	3
		S375F	T376A	D405N	R408S	K417N	N440K	S477N	T478K	E484A	Q493R	Q498R
		3	3	3	3	3	3	3	3	3	3	3
		N501Y	Y505H	D614G	H655Y	N679K	P681H	N764K	D796Y	Q954H	N969K	
		3	3	3	3	3	3	3	3	3	3	
1	BA.2.78	T19I	ΔL24	ΔP25	ΔP26	A27S	G142D	V213G	G339D	S371F	S373P	S375F
		1	1	1	1	1	1	1	1	1	1	1
		T376A	D405N	R408S	K417N	N440K	S477N	T478K	E484A	Q493R	Q498R	N501Y
		1	1	1	1	1	1	1	1	1	1	1
		Y505H	D614G	H655Y	N679K	P681H	N764K	D796Y	Q954H	N969K		
		1	1	1	1	1	1	1	1	1		
48	BA.4	T19I	ΔL24	ΔP25	ΔP26	A27S	ΔH69	ΔV70	G142D	V213G	G339D	S371F
		48	48	48	48	48	48	48	48	48	48	48
		S373P	S375F	T376A	D405N	R408S	K417N	N440K	L452R	S477N	T478K	E484A
		48	48	48	48	48	48	48	48	48	48	48
		F486V	Q498R	N501Y	Y505H	D614G	H655Y	N679K	P681H	N764K	D796Y	Q954H
		48	48	48	48	48	48	48	48	48	48	48
		N969K										
		48										
22	BA.4.1	V3G	T19I	ΔL24	ΔP25	ΔP26	A27S	ΔH69	ΔV70	G142D	V213G	G339D
		22	22	22	22	22	22	22	22	22	22	22
		S371F	S373P	S375F	T376A	D405N	R408S	K417N	N440K	L452R	S477N	T478K

		22	22	22	22	22	22	22	22	22	22	22
		E484A	F486V	Q498R	N501Y	Y505H	D614G	H655Y	N679K	P681H	N764K	D796Y
		22	19	22	22	22	22	22	22	22	22	22
		Q954H	N969K									
		22	22									
1	BA.4.1.1	V3G	T19I	ΔL24	ΔP25	ΔP26	A27S	ΔH69	ΔV70	G142D	V213G	G339D
		1	1	1	1	1	1	1	1	1	1	1
		S371F	S373P	S375F	T376A	D405N	R408S	K417N	N440K	L452R	S477N	T478K
		1	1	1	1	1	1	1	1	1	1	1
		E484A	F486V	Q498R	N501Y	Y505H	D614G	H655Y	N679K	P681H	N764K	D796Y
		1	1	1	1	1	1	1	1	1	1	1
		Q954H	N969K									
		1	1									
1	BA.4.1.5	V3G	T19I	ΔL24	ΔP25	ΔP26	A27S	ΔH69	ΔV70	G142D	V213G	G339D
		1	1	1	1	1	1	1	1	1	1	1
		S371F	S373P	S375F	T376A	D405N	R408S	K417N	N440K	L452R	S477N	T478K
		1	1	1	1	1	1	1	1	1	1	1
		E484A	F486V	Q498R	N501Y	Y505H	D614G	H655Y	N679K	P681H	N764K	D796Y
		1	1	1	1	1	1	1	1	1	1	1
		Q954H	N969K									
		1	1									
3	BA.4.4	T19I	ΔL24	ΔP25	ΔP26	A27S	ΔH69	ΔV70	G142D	V213G	G339D	S371F
		3	3	3	3	3	3	3	3	3	3	3
		S373P	S375F	T376A	D405N	R408S	K417N	N440K	L452R	S477N	T478K	E484A
		3	3	3	3	3	3	3	3	3	3	3
		F486V	Q498R	N501Y	Y505H	D614G	H655Y	N658S	N679K	P681H	N764K	D796Y
		3	3	3	3	3	3	3	3	3	3	3
		Q954H	N969K									
		3	3									
2	BA.4.6	T19I	ΔL24	ΔP25	ΔP26	A27S	ΔH69	ΔV70	G142D	V213G	G339D	R346T
		2	2	2	2	2	2	2	2	2	2	2

		S371F	S373P	S375F	T376A	D405N	R408S	K417N	N440K	L452R	S477N	T478K
		2	2	2	2	2	2	2	2	2	2	2
		E484A	F486V	Q498R	N501Y	Y505H	D614G	H655Y	N658S	N679K	P681H	N764K
		2	2	2	2	2	2	2	2	2	2	2
		D796Y	Q954H	N969K								
		2	2	2								
		T19I	ΔL24	ΔP25	ΔP26	A27S	ΔH69	ΔV70	G142D	V213G	G339D	R346S
		1	1	1	1	1	1	1	1	1	1	1
		S371F	S373P	S375F	T376A	D405N	R408S	K417N	N440K	L452R	S477N	T478K
1	BA.4.7	1	1	1	1	1	1	1	1	1	1	1
		E484A	F486V	Q498R	N501Y	Y505H	D614G	H655Y	N658S	N679K	P681H	N764K
		1	1	1	1	1	1	1	1	1	1	1
		D796Y	Q954H	N969K								
		1	1	1								
		T19I	ΔL24	ΔP25	ΔP26	A27S	ΔH69	ΔV70	G142D	V213G	G339D	S371F
		6	6	6	6	6	6	6	6	6	6	6
		S373P	S375F	T376A	D405N	R408S	K417N	N440K	L452R	S477N	T478K	E484A
6	BA.5	6	6	6	6	6	6	6	6	6	6	6
		F486V	Q498R	N501Y	Y505H	D614G	H655Y	N679K	P681H	N764K	D796Y	Q954H
		6	6	6	6	6	6	6	6	6	6	6
		N969K										
		6										
		T19I	ΔL24	ΔP25	ΔP26	A27S	ΔH69	ΔV70	G142D	V213G	G339D	S371F
		258	258	258	258	258	258	258	257	258	258	257
		S373P	S375F	T376A	D405N	R408S	K417N	N440K	L452R	S477N	T478K	E484A
258	BA.5.1	258	258	258	258	256	257	257	257	258	258	258
		F486V	Q498R	N501Y	Y505H	D614G	H655Y	N679K	P681H	N764K	D796Y	Q954H
		258	258	258	258	258	258	258	258	258	258	258
		N969K										
		258										
1	BA.5.1.1	T19I	ΔL24	ΔP25	ΔP26	A27S	ΔH69	ΔV70	G142D	V213G	G339D	S371F

		1	1	1	1	1	1	1	1	1	1	1
		S373P	S375F	T376A	D405N	R408S	K417N	N440K	L452R	S477N	T478K	E484A
		1	1	1	1	1	1	1	1	1	1	1
		F486V	Q498R	N501Y	Y505H	D614G	H655Y	N679K	P681H	N764K	D796Y	Q954H
		1	1	1	1	1	1	1	1	1	1	1
		N969K										
		1										
8	BA.5.1.2	T19I	ΔL24	ΔP25	ΔP26	A27S	ΔH69	ΔV70	G142D	V213G	G339D	S371F
		8	8	8	8	8	8	8	8	8	8	8
		S373P	S375F	T376A	D405N	R408S	K417N	N440K	L452R	S477N	T478K	E484A
		8	8	8	8	8	8	8	8	8	8	8
		F486V	Q498R	N501Y	Y505H	D614G	H655Y	N679K	P681H	N764K	D796Y	Q954H
		8	8	8	8	8	8	8	8	8	8	8
		N969K										
		8										
13	BA.5.1.3	T19I	ΔL24	ΔP25	ΔP26	A27S	ΔH69	ΔV70	G142D	V213G	V289I	G339D[d]
		13	13	13	13	13	13	13	13	13	13	9
		S371F[d]	S373P	S375F	T376A	D405N	R408S	K417N	N440K	L452R	S477N	T478K
		7	10	10	11	13	13	13	13	13	13	13
		E484A	F486V	Q498R	N501Y	Y505H	D614G	H655Y	N679K	P681H	N764K	D796Y
		13	13	13	13	13	13	13	13	13	13	13
		Q954H	N969K									
		13	13									
3	BA.5.1.5	T19I	ΔL24	ΔP25	ΔP26	A27S	ΔH69	ΔV70	G142D	V213G	G339D	S371F
		3	3	3	3	3	3	3	3	3	3	3
		S373P	S375F	T376A	D405N	R408S	K417N	N440K	L452R	S477N	T478K	E484A
		3	3	3	3	3	3	3	3	3	3	3
		F486V	Q498R	N501Y	Y505H	D614G	H655Y	N679K	P681H	N764K	D796Y	Q954H
		3	3	3	3	3	3	3	3	3	3	3
		N969K	P1263Q									
		3	3									

		T19I	ΔL24	ΔP25	ΔP26	A27S	ΔH69	ΔV70	G142D	V213G	G339D	S371F
		2	2	2	2	2	2	2	2	2	2	2
		S373P	S375F	T376A	D405N	R408S	K417N	N440K	L452R	S477N	T478K	E484A
2	BA.5.1.10	2	2	2	2	2	2	2	2	2	2	2
		F486V	Q498R	N501Y	Y505H	D614G	H655Y	N679K	P681H	N764K	D796Y	Q954H
		2	2	2	2	2	2	2	2	2	2	2
		N969K										
		2										
		T19I	ΔL24	ΔP25	ΔP26	A27S	ΔH69	ΔV70	G142D	V213G	G339D	S371F
		1	1	1	1	1	1	1	1	1	1	1
		S373P	S375F	T376A	D405N	R408S	K417N	N440K	L452R	S477N	T478K	E484A
1	BK.1	1	1	1	1	1	1	1	1	1	1	1
		F486V	Q498R	N501Y	Y505H	D614G	H655Y	N679K	P681H	N764K	D796Y	Q954H
		1	1	1	1	1	1	1	1	1	1	1
		N969K										
		1										
		T19I	ΔL24	ΔP25	ΔP26	A27S	ΔH69	ΔV70	G142D	V213G	G339D	S371F
		3	3	3	3	3	3	3	3	3	3	3
		S373P	S375F	T376A	D405N	R408S	K417N	N440K	V445A	L452R	S477N	T478K
3	BA.5.1.12	3	3	3	3	3	3	3	2	3	3	3
		E484A	F486V	Q498R	N501Y	Y505H	D614G	H655Y	N679K	P681H	N764K	D796Y
		3	3	3	3	3	3	3	3	3	3	3
		Q954H	N969K									
		3	3									
		T19I	ΔL24	ΔP25	ΔP26	A27S	ΔH69	ΔV70	G142D	V213G	G339D	S371F
		1	1	1	1	1	1	1	1	1	1	1
		S373P	S375F	T376A	D405N	R408S	K417N	N440K	L452R	S477N	T478K	E484A
1	BA.5.1.17	1	1	1	1	1	1	1	1	1	1	1
		F486V	Q498R	N501Y	Y505H	D614G	H655Y	N679K	P681H	N764K	D796Y	Q954H
		1	1	1	1	1	1	1	1	1	1	1
		N969K										

		1										
2	BA.5.1.22	T19I	ΔL24	ΔP25	ΔP26	A27S	ΔH69	ΔV70	G142D	V213G	G339D	S371F
		2	2	2	2	2	2	2	2	2	2	2
		S373P	S375F	T376A	D405N	R408S	K417N	N440K	L452R	S477N	T478K	E484A
		2	2	2	2	2	2	2	2	2	2	2
		F486V	Q498R	N501Y	Y505H	D614G	H655Y	N679K	P681H	N764K	D796Y	Q954H
		2	2	2	2	2	2	2	2	2	2	2
		N969K										
		2										
2	BA.5.1.23	T19I	ΔL24	ΔP25	ΔP26	A27S	ΔH69	ΔV70	G142D	V213G	G339D	S371F
		2	2	2	2	2	2	2	2	2	2	2
		S373P	S375F	T376A	D405N	R408S	K417N	N440K	L452R	S477N	T478K	E484A
		2	2	2	2	2	2	2	2	2	2	2
		F486V	Q498R	N501Y	Y505H	D614G	H655Y	N679K	P681H	N764K	D796Y	Q954H
		2	2	2	2	2	2	2	2	2	2	2
		N969K										
		2										
223	BA.5.2	T19I	ΔL24	ΔP25	ΔP26	A27S	ΔH69	ΔV70	G142D	V213G	G339D	S371F
		223	223	223	223	223	223	223	223	223	223	223
		S373P	S375F	T376A	D405N	R408S	K417N	N440K	L452R	S477N	T478K	E484A
		223	223	223	223	223	223	223	223	223	223	223
		F486V	Q498R	N501Y	Y505H	D614G	H655Y	N679K	P681H	N764K	D796Y	Q954H
		223	223	223	223	223	222	223	223	223	223	223
		N969K										
		223										
139	BA.5.2.1	T19I	ΔL24	ΔP25	ΔP26	A27S	ΔH69	ΔV70	G142D	V213G	G339D	S371F
		139	139	139	139	139	139	139	139	137	139	139
		S373P	S375F	T376A	D405N	R408S	K417N	N440K	L452R	S477N	T478K	E484A
		139	139	139	139	139	139	139	139	139	139	139
		F486V	Q498R	N501Y	Y505H	D614G	H655Y	N679K	P681H	N764K	D796Y	Q954H
		139	139	139	139	138	138	139	139	139	139	139

		N969K										
		139										
11	BF.1	T19I	ΔL24	ΔP25	ΔP26	A27S	ΔH69	ΔV70	G142D	V213G	G339D	S371F
		11	11	11	11	11	11	11	11	11	11	11
		S373P	S375F	T376A	D405N	R408S	K417N	N440K	L452R	S477N	T478K	E484A
		11	11	11	11	11	11	11	11	11	11	11
		F486V	Q498R	N501Y	Y505H	D614G	H655Y	N679K	P681H	N764K	D796Y	Q954H
		11	11	11	11	11	11	11	11	11	11	11
		N969K										
		11										
1	BF.2	T19I	ΔL24	ΔP25	ΔP26	A27S	ΔH69	ΔV70	G142D	V213G	G339D	S371F
		1	1	1	1	1	1	1	1	1	1	1
		S373P	S375F	T376A	D405N	R408S	K417N	N440K	L452R	S477N	T478K	E484A
		1	1	1	1	1	1	1	1	1	1	1
		F486V	Q498R	N501Y	Y505H	D614G	H655Y	N679K	P681H	N764K	D796Y	Q954H
		1	1	1	1	1	1	1	1	1	1	1
		N969K										
		1										
3	BF.4	T19I	ΔL24	ΔP25	ΔP26	A27S	ΔH69	ΔV70	G142D	V213G	T259A	G339D
		3	3	3	3	3	3	3	3	3	3	3
		S371F	S373P	S375F	T376A	D405N	R408S	K417N	N440K[d]	L452R[d]	S477N	T478K
		3	3	3	3	3	3	3	2	2	3	3
		E484A	F486V	Q498R	N501Y	Y505H	D614G	H655Y	N679K	P681H	N764K	D796Y
		3	3	3	3	3	3	3	3	3	3	3
		Q954H	N969K									
		3	3									
25	BF.5	T19I	ΔL24	ΔP25	ΔP26	A27S	ΔH69	ΔV70	G142D	V213G	G339D	S371F
		25	25	25	25	25	25	25	25	25	25	25
		S373P	S375F	T376A	D405N	R408S	K417N	N440K	L452R	S477N	T478K	E484A
		25	25	25	25	25	25	25	25	25	25	25
		F486V	Q498R	N501Y	Y505H	D614G	H655Y	N679K	P681H	N764K	D796Y	Q954H

		25	25	25	25	25	25	25	25	25	25	25
		N969K	A1020S									
		25	25									
15	BF.7	T19I	ΔL24	ΔP25	ΔP26	A27S	ΔH69	ΔV70	G142D	V213G	G339D	R346T
		15	15	15	15	15	15	15	15	15	15	15
		S371F	S373P	S375F	T376A	D405N	R408S	K417N	N440K	L452R	S477N	T478K
		15	15	15	15	15	15	15	15	15	15	15
		E484A	F486V	Q498R	N501Y	Y505H	D614G	H655Y	N679K	P681H	N764K	D796Y
		15	15	15	15	15	15	15	15	15	15	15
		Q954H	N969K									
		15	15									
2	BF.7.1	T19I	ΔL24	ΔP25	ΔP26	A27S	ΔH69	ΔV70	G142D	V213G	G261V	G339D
		2	2	2	2	2	2	2	2	2	2	2
		R346T	S371F	S373P	S375F	T376A	D405N	R408S	K417N	N440K	L452R	S477N
		2	2	2	2	2	2	2	2	2	2	2
		T478K	E484A	F486V	Q498R	N501Y	Y505H	D614G	H655Y	N679K	P681H	N764K
		2	2	2	2	2	2	2	2	2	2	2
		D796Y	Q954H	N969K								
		2	2	2								
1	BF.7.4.2	T19I	ΔP25	ΔP26	ΔH69	ΔV70	G142D	V213G	G339D	R346T	S371F	S373P
		1	1	1	1	1	1	1	1	1	1	1
		S375F	T376A	D405N	R408S	K417N	N440K	L452R	S477N	T478K	E484A	F486V
		1	1	1	1	1	1	1	1	1	1	1
		Q498R	N501Y	Y505H	D614G	H655Y	N679K	P681H	N764K	D796Y	Q954H	N969K
		1	1	1	1	1	1	1	1	1	1	1
1	BF.7.5	T19I	ΔL24	ΔP25	ΔP26	A27S	ΔH69	ΔV70	G142D	V213G	G339D	R346T
		1	1	1	1	1	1	1	1	1	1	1
		S371F	S373P	S375F	T376A	D405N	R408S	K417N	N440K	L452R	S477N	T478K
		1	1	1	1	1	1	1	1	1	1	1
		E484A	F486V	Q498R	N501Y	Y505H	D614G	H655Y	N679K	P681H	N764K	D796Y
		1	1	1	1	1	1	1	1	1	1	1

		Q954H	N969K									
		1	1									
1	BF.7.5.1	T19I	ΔL24	ΔP25	ΔP26	A27S	ΔH69	ΔV70	G142D	ΔY144	G181V	V213G
		1	1	1	1	1	1	1	1	1	1	1
		G339D	R346T	S371F	S373P	S375F	T376A	D405N	R408S	K417N	N440K	L452R
		1	1	1	1	1	1	1	1	1	1	1
		S477N	T478K	E484A	F486V	Q498R	N501Y	Y505H	D614G	H655Y	N679K	P681H
		1	1	1	1	1	1	1	1	1	1	1
		N764K	D796Y	Q954H	N969K							
		1	1	1	1							
1	BF.7.8	T19I	ΔL24	ΔP25	ΔP26	A27S	ΔH69	ΔV70	G142D	V213G	G339D	R346T
		1	1	1	1	1	1	1	1	1	1	1
		S371F	S373P	S375F	T376A	D405N	R408S	K417N	N440K	L452R	S477N	T478K
		1	1	1	1	1	1	1	1	1	1	1
		E484A	F486V	Q498R	N501Y	Y505H	D614G	H655Y	N679K	P681H	N764K	D796Y
		1	1	1	1	1	1	1	1	1	1	1
		T883I	Q954H	N969K								
		1	1	1								
5	BF.10	T19I	ΔL24	ΔP25	ΔP26	A27S	ΔH69	ΔV70	G142D	V213G	G339D	S371F
		5	5	5	5	5	5	5	5	5	5	5
		S373P	S375F	T376A	D405N	R408S	K417N	N440K	L452R	S477N	T478K	E484A
		5	5	5	5	5	5	5	5	5	5	5
		F486V	Q498R	N501Y	Y505H	D614G	H655Y	N679K	P681H	N764K	D796Y	Q954H
		5	5	5	5	5	5	5	5	5	5	5
		N969K										
		5										
11	BF.11	T19I	ΔL24	ΔP25	ΔP26	A27S	ΔH69	ΔV70	G142D	V213G	G339D	R346T
		11	11	11	11	11	11	11	11	11	11	11
		S371F	S373P	S375F	T376A	D405N	R408S	K417N	N440K	L452R	S477N	T478K
		11	11	11	11	11	11	11	11	11	11	11
		E484A	F486V	Q498R	N501Y	Y505H	D614G	H655Y	N679K	P681H	N764K	D796Y

		11	11	11	11	11	11	11	11	11	11	11
		Q954H	N969K									
		11	11									
1	BF.11.2	T19I	ΔL24	ΔP25	ΔP26	A27S	ΔH69	ΔV70	G142D	K147N	V213G	G339D
		1	1	1	1	1	1	1	1	1	1	1
		R346T	S371F	S373P	S375F	T376A	D405N	R408S	K417N	N440K	L452R	S477N
		1	1	1	1	1	1	1	1	1	1	1
		T478K	E484A	F486V	Q498R	N501Y	Y505H	D614G	H655Y	N679K	P681H	N764K
		1	1	1	1	1	1	1	1	1	1	1
		D796Y	Q954H	N969K								
		1	1	1								
1	BF.14	T19I	ΔL24	ΔP25	ΔP26	A27S	ΔH69	ΔV70	G142D	V213G	G339D	S371F
		1	1	1	1	1	1	1	1	1	1	1
		S373P	S375F	T376A	D405N	R408S	K417N	N440K	N450D	L452R	S477N	T478K
		1	1	1	1	1	1	1	1	1	1	1
		E484A	F486V	Q498R	N501Y	Y505H	D614G	H655Y	N679K	P681H	N764K	D796Y
		1	1	1	1	1	1	1	1	1	1	1
		Q954H	N969K									
		1	1									
1	BF.19	T19I	ΔL24	ΔP25	ΔP26	A27S	ΔH69	ΔV70	G142D	V213E	G339D	S371F
		1	1	1	1	1	1	1	1	1	1	1
		S373P	S375F	T376A	D405N	R408S	K417N	N440K	L452R	S477N	T478K	E484A
		1	1	1	1	1	1	1	1	1	1	1
		F486V	Q498R	N501Y	Y505H	D614G	H655Y	N679K	P681H	N764K	D796Y	Q954H
		1	1	1	1	1	1	1	1	1	1	1
		N969K										
		1										
1	BA.5.2.2	T19I	ΔL24	ΔP25	ΔP26	A27S	ΔH69	ΔV70	G142D	V213G	G339D	S371F
		1	1	1	1	1	1	1	1	1	1	1
		S373P	S375F	T376A	D405N	R408S	K417N	N440K	L452R	S477N	T478K	E484A
		1	1	1	1	1	1	1	1	1	1	1

		F486V	Q498R	N501Y	Y505H	D614G	H655Y	N679K	P681H	N764K	D796Y	Q954H
		1	1	1	1	1	1	1	1	1	1	1
		N969K										
		1										
5	BA.5.2.3	T19I	ΔL24	ΔP25	ΔP26	A27S	ΔH69	ΔV70	G142D	V213G	G339D	S371F
		5	5	5	5	5	5	5	5	5	5	5
		S373P	S375F	T376A	D405N	R408S	K417N	N440K	L452R	S477N	T478K	E484A
		5	5	5	5	5	5	5	5	5	5	5
		F486V	Q498R	N501Y	Y505H	D614G	H655Y	N679K	P681H	N764K	D796Y	Q954H
		5	5	5	5	5	5	5	5	5	5	5
		N969K										
		5										
1	BA.5.2.13	T19I	ΔL24	ΔP25	ΔP26	A27S	ΔH69	ΔV70	G142D	V213G	G339D	R346T
		1	1	1	1	1	1	1	1	1	1	1
		S371F	S373P	S375F	T376A	D405N	R408S	K417N	N440K	L452R	S477N	T478K
		1	1	1	1	1	1	1	1	1	1	1
		E484A	F486V	Q498R	N501Y	Y505H	D614G	H655Y	N679K	P681H	N764K	D796Y
		1	1	1	1	1	1	1	1	1	1	1
		Q954H	N969K									
		1	1									
1	BA.5.2.14	T19I	ΔL24	ΔP25	ΔP26	A27S	W64L	ΔH69	ΔV70	G142D	V213G	G339D
		1	1	1	1	1	1	1	1	1	1	1
		S371F	S373P	S375F	T376A	D405N	R408S	K417N	N440K	K444M	L452R	S477N
		1	1	1	1	1	1	1	1	1	1	1
		T478K	E484A	F486V	Q498R	N501Y	Y505H	D614G	H655Y	N679K	P681H	N764K
		1	1	1	1	1	1	1	1	1	1	1
		D796Y	Q954H	N969K								
		1	1	1								
1	BA.5.2.18	T19I	ΔL24	ΔP25	ΔP26	A27S	ΔH69	ΔV70	G142D	V213G	G339D	S371F
		1	1	1	1	1	1	1	1	1	1	1
		S373P	S375F	T376A	D405N	R408S	K417N	N440K	K444R	L452R	S477N	T478K

		1	1	1	1	1	1	1	1	1	1	1
		E484A	F486V	Q498R	N501Y	Y505H	D614G	H655Y	N679K	P681H	N764K	D796Y
		1	1	1	1	1	1	1	1	1	1	1
		Q954H	N969K									
		1	1									
12	BA.5.2.20	T19I	ΔL24	ΔP25	ΔP26	A27S	ΔH69	ΔV70	G142D	V213G	G339D	S371F
		12	12	12	12	12	12	12	12	12	12	12
		S373P	S375F	T376A	D405N	R408S	K417N	N440K	L452R	S477N	T478K	E484A
		12	12	12	12	12	12	12	12	12	12	12
		F486V	Q498R	N501Y	Y505H	D614G	H655Y	N679K	P681H	N764K	D796Y	Q954H
		12	12	12	12	12	12	12	12	12	12	12
		N969K										
		12										
1	BA.5.2.22	T19I	ΔL24	ΔP25	ΔP26	A27S	ΔH69	ΔV70	G142D	V213G	G339D	S371F
		1	1	1	1	1	1	1	1	1	1	1
		S373P	S375F	T376A	D405N	R408S	K417N	N440K	L452R	S477N	T478K	E484A
		1	1	1	1	1	1	1	1	1	1	1
		F486V	Q498R	N501Y	Y505H	D614G	H655Y	N679K	P681H	N764K	D796Y	Q954H
		1	1	1	1	1	1	1	1	1	1	1
		N969K										
		1										
1	CK.2	T19I	ΔL24	ΔP25	ΔP26	A27S	ΔH69	ΔV70	G142D	V213G	G339D	S371F
		1	1	1	1	1	1	1	1	1	1	1
		S373P	S375F	T376A	D405N	R408S	K417N	N440K	K444N	L452R	S477N	T478K
		1	1	1	1	1	1	1	1	1	1	1
		E484A	F486V	Q498R	N501Y	Y505H	D614G	H655Y	N679K	P681H	N764K	D796Y
		1	1	1	1	1	1	1	1	1	1	1
		Q954H	N969K									
		1	1									
1	BA.5.2.26	T19I	ΔL24	ΔP25	ΔP26	A27S	ΔH69	ΔV70	G142D	V213G	G339D	S371F
		1	1	1	1	1	1	1	1	1	1	1

		S373P	S375F	T376A	D405N	R408S	K417N	N440K	L452R	S477N	T478K	E484A
		1	1	1	1	1	1	1	1	1	1	1
		F486V	Q498R	N501Y	Y505H	D614G	H655Y	N679K	P681H	N764K	D796Y	Q954H
		1	1	1	1	1	1	1	1	1	1	1
		N969K										
		1										
2	BA.5.2.33	T19I	ΔL24	ΔP25	ΔP26	A27S	ΔH69	ΔV70	G142D	V213G	G339D	S371F
		2	2	2	2	2	2	2	2	2	2	2
		S373P	S375F	T376A	D405N	R408S	K417N	N440K	L452R	S477N	T478K	E484A
		2	2	2	2	2	2	2	2	2	2	2
		F486V	Q498R	N501Y	Y505H	D614G	H655Y	N679K	P681H	N764K	D796Y	Q954H
		2	2	2	2	2	2	2	2	2	2	2
		N969K										
		2										
1	BA.5.2.35	T19I	ΔL24	ΔP25	ΔP26	A27S	ΔH69	ΔV70	G142D	V213G	G339D	R346T
		1	1	1	1	1	1	1	1	1	1	1
		S371F	S373P	S375F	T376A	D405N	R408S	K417N	N440K	L452R	S477N	T478K
		1	1	1	1	1	1	1	1	1	1	1
		E484A	F486V	Q498R	N501Y	Y505H	D614G	H655Y	N679K	P681H	N764K	D796Y
		1	1	1	1	1	1	1	1	1	1	1
		Q954H	N969K									
		1	1									
1	BA.5.3	T19I	ΔL24	ΔP25	ΔP26	A27S	ΔH69	ΔV70	G142D	V213G	G339D	S371F
		1	1	1	1	1	1	1	1	1	1	1
		S373P	S375F	T376A	D405N	R408S	K417N	N440K	L452R	S477N	T478K	E484A
		1	1	1	1	1	1	1	1	1	1	1
		F486V	Q498R	N501Y	Y505H	D614G	H655Y	N679K	P681H	N764K	D796Y	Q954H
		1	1	1	1	1	1	1	1	1	1	1
		N969K										
		1										
1	BA.5.3.1	T19I	ΔL24	ΔP25	ΔP26	A27S	ΔH69	ΔV70	G142D	V213G	G339D	S371F

		1	1	1	1	1	1	1	1	1	1	1
		S373P	S375F	T376A	D405N	R408S	K417N	N440K	L452R	S477N	T478K	E484A
		1	1	1	1	1	1	1	1	1	1	1
		F486V	Q498R	N501Y	Y505H	D614G	H655Y	N679K	P681H	N764K	D796Y	Q954H
		1	1	1	1	1	1	1	1	1	1	1
		N969K										
		1										
30	BE.1	T19I	ΔL24	ΔP25	ΔP26	A27S	ΔH69	ΔV70	G142D	V213G	G339D	S371F
		30	30	30	30	30	30	30	30	30	29	29
		S373P	S375F	T376A	D405N	R408S	K417N	N440K	L452R	S477N	T478K	E484A
		30	30	30	30	30	30	30	30	30	30	30
		F486V	Q498R	N501Y	Y505H	D614G	H655Y	N679K	P681H	N764K	D796Y	Q954H
		30	30	30	30	30	30	30	30	30	30	30
		N969K										
		30										
28	BE.1.1	T19I	ΔL24	ΔP25	ΔP26	A27S	ΔH69	ΔV70	G142D	V213G	G339D	S371F
		28	28	28	28	28	28	28	28	28	28	28
		S373P	S375F	T376A	D405N	R408S	K417N	N440K	L452R	S477N	T478K	E484A
		28	28	28	28	28	28	28	28	28	28	27
		F486V	Q498R	N501Y	Y505H	D614G	H655Y	N679K	P681H	N764K	D796Y	Q954H
		28	28	28	28	28	28	28	28	28	28	28
		N969K										
		28										
4	BE.1.1.1	T19I	ΔL24	ΔP25	ΔP26	A27S	ΔH69	ΔV70	G142D	V213G	G339D	S371F
		4	4	4	4	4	4	4	4	4	4	4
		S373P	S375F	T376A	D405N	R408S	K417N	N440K	K444T	L452R	S477N	T478K
		4	4	4	4	4	4	4	4	4	4	4
		E484A	F486V	Q498R	N501Y	Y505H	D614G	H655Y	N679K	P681H	N764K	D796Y
		4	4	4	4	4	4	4	4	4	4	4
		Q954H	N969K									
		4	4									

2	BQ.1	T19I	ΔL24	ΔP25	ΔP26	A27S	ΔH69	ΔV70	G142D	V213G	G339D	S371F
		2	2	2	2	2	2	2	2	2	2	2
		S373P	S375F	T376A	D405N	R408S	K417N	N440K	K444T	L452R	N460K	S477N
		2	2	2	2	2	2	2	2	2	2	2
		T478K	E484A	F486V	Q498R	N501Y	Y505H	D614G	H655Y	N679K	P681H	N764K
		2	2	2	2	2	2	2	2	2	2	2
		D796Y	Q954H	N969K								
		2	2	2								
7	BQ.1.1	T19I	ΔL24	ΔP25	ΔP26	A27S	ΔH69	ΔV70	G142D	V213G	G339D	R346T
		7	7	7	7	7	7	7	7	7	7	7
		S371F	S373P	S375F	T376A	D405N	R408S	K417N	N440K	K444T	L452R	N460K
		7	7	7	7	7	7	7	7	7	7	7
		S477N	T478K	E484A	F486V	Q498R	N501Y	Y505H	D614G	H655Y	N679K	P681H
		7	7	7	7	7	7	7	7	7	7	7
		N764K	D796Y	Q954H	N969K							
		7	7	7	7							
1	BQ.1.1.1	T19I	ΔL24	ΔP25	ΔP26	A27S	ΔH69	ΔV70	G142D	V213G	G339D	R346T
		1	1	1	1	1	1	1	1	1	1	1
		S371F	S373P	S375F	T376A	D405N	R408S	K417N	N440K	K444T	L452R	N460K
		1	1	1	1	1	1	1	1	1	1	1
		S477N	T478K	E484A	F486V	Q498R	N501Y	Y505H	D614G	H655Y	N679K	P681H
		1	1	1	1	1	1	1	1	1	1	1
		N764K	D796Y	Q954H	N969K							
		1	1	1	1							
2	BQ.1.1.11	T19I	ΔL24	ΔP25	ΔP26	A27S	ΔH69	ΔV70	G142D	V213G	G339D	R346T
		2	2	2	2	2	2	2	2	2	2	2
		S371F	S373P	S375F	T376A	D405N	R408S	K417N	N440K	K444T	L452R	N460K
		2	2	2	2	2	2	2	2	2	2	2
		S477N	T478K	E484A	F486V	Q498R	N501Y	Y505H	D614G	H655Y	N679K	P681H
		2	2	2	2	2	2	2	2	2	2	2
		N764K	D796Y	Q954H	N969K							

		2	2	2	2							
1	BQ.1.3	T19I	ΔL24	ΔP25	ΔP26	A27S	ΔH69	ΔV70	G142D	V213G	G339D	S371F
		1	1	1	1	1	1	1	1	1	1	1
		S373P	S375F	T376A	D405N	R408S	K417N	N440K	K444T	L452R	N460K	S477N
		1	1	1	1	1	1	1	1	1	1	1
		T478K	E484A	F486V	Q498R	N501Y	Y505H	D614G	E619Q	H655Y	N679K	P681H
		1	1	1	1	1	1	1	1	1	1	1
		N764K	D796Y	Q954H	N969K							
		1	1	1	1							
2	BQ.1.5	T19I	ΔL24	ΔP25	ΔP26	A27S	ΔH69	ΔV70	G142D	V213G	G339D	S371F
		2	2	2	2	2	2	2	2	2	2	2
		S373P	S375F	T376A	D405N	R408S	K417N	N440K	K444T	L452R	N460K	S477N
		2	2	2	2	2	2	2	2	2	2	2
		T478K	E484A	F486V	Q498R	N501Y	Y505H	D614G	H655Y	N679K	P681H	N764K
		2	2	2	2	2	2	2	2	2	2	2
		D796Y	Q954H	N969K								
		2	2	2								
1	BQ.1.8	T19I	ΔL24	ΔP25	ΔP26	A27S	ΔH69	ΔV70	G142D	ΔY144	V213G	G339D
		1	1	1	1	1	1	1	1	1	1	1
		S371F	S373P	S375F	T376A	D405N	R408S	K417N	N440K	K444T	L452R	N460K
		1	1	1	1	1	1	1	1	1	1	1
		S477N	T478K	E484A	F486V	Q498R	N501Y	Y505H	D614G	H655Y	N679K	P681H
		1	1	1	1	1	1	1	1	1	1	1
		N764K	D796Y	Q954H	N969K							
		1	1	1	1							
1	BA.5.3.2	T19I	ΔL24	ΔP25	ΔP26	A27S	ΔH69	ΔV70	G142D	V213G	G339D	S371F
		1	1	1	1	1	1	1	1	1	1	1
		S373P	S375F	T376A	D405N	R408S	K417N	N440K	L452R	S477N	T478K	E484A
		1	1	1	1	1	1	1	1	1	1	1
		F486V	Q498R	N501Y	Y505H	D614G	H655Y	N679K	P681H	N764K	D796Y	Q954H
		1	1	1	1	1	1	1	1	1	1	1

		N969K										
		1										
7	BA.5.3.3	T19I	ΔL24	ΔP25	ΔP26	A27S	ΔH69	ΔV70	G142D	V213G	G339D	S371F
		7	7	7	7	7	7	7	7	7	7	7
		S373P	S375F	T376A	D405N	R408S	K417N	N440K	L452R	S477N	T478K	E484A
		7	7	7	7	7	7	7	7	7	7	7
		F486V	Q498R	N501Y	Y505H	D614G	H655Y	N679K	P681H	N764K	D796Y	Q954H
		7	7	7	7	7	7	7	7	7	7	7
		N969K										
		7										
1	BA.5.3.4	T19I	ΔL24	ΔP25	ΔP26	A27S	ΔH69	ΔV70	G142D	V213G	G339D	S371F
		1	1	1	1	1	1	1	1	1	1	1
		S373P	S375F	T376A	D405N	R408S	K417N	N440K	L452R	S477N	T478K	E484A
		1	1	1	1	1	1	1	1	1	1	1
		F486V	Q498R	N501Y	Y505H	D614G	H655Y	N679K	P681H	N764K	D796Y	Q954H
		1	1	1	1	1	1	1	1	1	1	1
		N969K										
		1										
6	BA.5.5	T19I	ΔL24	ΔP25	ΔP26	A27S	ΔH69	ΔV70	T76I	G142D	V213G	G339D
		6	6	6	6	6	6	6	6	6	6	6
		S371F	S373P	S375F	T376A	D405N	R408S	K417N	N440K	L452R	S477N	T478K
		6	6	6	6	6	6	6	6	6	6	6
		E484A	F486V	Q498R	N501Y	Y505H	D614G	H655Y	N679K	P681H	N764K	D796Y
		6	6	6	6	6	6	6	6	6	6	6
		Q954H	N969K									
		6	6									
5	BA.5.6	T19I	ΔL24	ΔP25	ΔP26	A27S	ΔH69	ΔV70	G142D	V213G	G339D	S371F
		5	5	5	5	5	5	5	5	5	5	5
		S373P	S375F	T376A	D405N	R408S	K417N	N440K	L452R	S477N	T478K	E484A
		5	5	5	5	5	5	5	5	5	5	5
		F486V	Q498R	N501Y	Y505H	D614G	H655Y	N679K	P681H	N764K	D796Y	Q954H

		5	5	5	5	5	5	5	5	5	5	5
		N969K										
		5										
2	BA.5.8	T19I	ΔL24	ΔP25	ΔP26	A27S	ΔH69	ΔV70	G142D	V213G	G339D	S371F
		2	2	2	2	2	2	2	2	2	2	2
		S373P	S375F	T376A	D405N	R408S	K417N	N440K	L452R	S477N	T478K	E484A
		2	2	2	2	2	2	2	2	2	2	2
		F486V	Q498R	N501Y	Y505H	D614G	H655Y	N679K	P681H	N764K	D796Y	Q954H
		2	2	2	2	2	2	2	2	2	2	2
		N969K	P1162L									
		2	2									
11	BA.5.9	T19I	ΔL24	ΔP25	ΔP26	A27S	ΔH69	ΔV70	G142D	V213G	G339D	R346I
		11	11	11	11	11	11	11	11	11	11	11
		S371F	S373P	S375F	T376A	D405N	R408S	K417N	N440K	L452R	S477N	T478K
		11	11	11	11	11	11	11	11	11	11	11
		E484A	F486V	Q498R	N501Y	Y505H	D614G	H655Y	N679K	P681H	N764K	D796Y
		11	11	11	11	11	11	11	11	11	11	11
		Q954H	N969K									
		11	11									

[a]The most prevalent mutations shown in Table S1 were determined in accordance to the most prevalent mutations of these lineages in this dataset, and as denoted by the CoV-Spectrum [1] (http//cov-spectrum.org/)(date last accessed 23 May 2023) and Outbreak.info [2] (http//outbreak.info/)(date last accessed 23 May 2023) websites.

[b]Total number of sequences that were identified for each lineage in this dataset.

[c]For lineages that were identified in lower frequency in this dataset, the CoV-Spectrum [1] (http//cov-spectrum.org/)(date last accessed 23 May 2023) and Outbreak.info [2] (http//outbreak.info/)(date last accessed 23 May 2023) websites were used to indicate the expected common mutations and deletions

[d]Mutations that are listed as common (above 75% prevalence) by CoV-Spectrum [1] (http//cov-spectrum.org/)(date last accessed 23 May 2023) and Outbreak.info [2] (http//outbreak.info/)(date last accessed 23 May 2023) websites, for a specific lineage but were found in lower frequencies (below 75%) in this dataset.

[e]Mutations that are listed as below 75% prevalence in CoV-Spectrum [1] (http//cov-spectrum.org/)(date last accessed 23 May 2023) and Outbreak.info [2] (http//outbreak.info/)(date last accessed 23 May 2023) websites, but were found in higher frequencies (above 75%) in this dataset for specific lineages.

Table S2. Uncommon S Protein Mutations of Delta and Omicron Lineages that were Identified in this Dataset[a].

Total Sequences[b]	Lineage[c]	Mutations and Number of Sequences Identified for Each Mutation										
47	B.1.617.2	S60F	A67V	ΔH69	H69Y	ΔV70	P85L	T95I	ΔG142	ΔV143	ΔY144	Y145D
		1	2	2	1	2	1	34	2	2	3	2
		R237K	R273T	A372V	Q474H	P521S	E554Q	T572I	Q677H	A688V	P812L	A1020V
		1	1	2	2	1	6	3	3	2	1	1
		P1162S	G1167V									
		2	1									
2	AY.3	Q613H	A1020S	V1176F								
		1	1	1								
106	AY.4	L5F	V6F	L8V	S13T	L18F	T33I	A67V	ΔH69	ΔV70	R102S	D138H
		3	1	1	4	3	3	4	4	4	1	3
		ΔG142	ΔV143	ΔY144	Y145D	E180V	G181V	L189F	P209S	A222S	A222V	D228G
		2	3	2	2	2	5	1	1	1	2	3
		F238L	W258L	G339V	N501S	E554G	E554R	T572I	P809S	T859I	S939F	L1063F
		1	1	1	1	1	1	6	1	2	2	1
		V1104L	G1131A	L1141F								
		1	1	1								
38	AY.4.2	V36F	A67V	ΔH69	ΔV70	T73I	ΔG142	ΔV143	ΔY144	Y145D	E154A	D228H
		25	3	3	3	1	2	2	2	2	5	1
		P809S	S1252F									
		1	1									
4	AY.4.5	L18F										
		1										
3	AY.5	L5F	V62L									
		1	1									
2	AY.6	T22I	A67V	T95I								
		2	2	1								
22	AY.7.2	G142D	A260V									
		5	1									
5	AY.9.2	P25S	P26S	F490S								

		1	1	1								
1	AY.23	S254F										
		1										
1	AY.25	S112L										
		1										
1	AY.34	A67V	ΔH69	ΔV70	K854E							
		1	1	1	1							
4	AY.36	L5F	G142D	A222V	E484Q	V1122L						
		1	1	1	1	1						
1	AY.42	G142D	D215Y									
		1	1									
197	AY.43	L5F	P26L	N30H	H49Y	S50A	T51S	A67V	ΔH69	H69Y	ΔV70	G75V
		4	1	1	1	1	1	5	6	1	6	3
		R78S	D80Y	T95I	S112L	ΔG142	G142D	ΔV143	ΔY144	Y145D	Q173H	G181V
		2	1	12	1	1	54	1	5	1	1	3
		V193L	D215H	P217S	H245Y	D253G	T323I	N354K	P463S	P621S	ΔN679	S680T
		2	1	1	1	46	1	1	1	41	1	1
		T739I	L752F	T859I	A890V	K921E	D950S	N955S	M1029V	A1078S	H1088Y	V1122L
		1	1	1	1	1	1	1	1	48	1	2
		G1124V	V1176F	I1227L	C1247F	S1252P	D1259Y	D1260Y				
		5	10	1	2	1	3	15				
1	AY.43.8	T323I										
		1										
4	AY.46.6	G142D										
		1										
2	AY.60	G142D										
		2										
32	AY.98	V70A	V90F	P174S	T376I	N440Y	T523I	T1009I	T1120I	V1176F		
		1	2	1	1	2	1	1	17	1		
11	AY.98.1	D80Y	S98F									
		1	3									

1	AY.107	L5F	D80Y	G142D								
		1	1	1								
1	AY.109	S151G	E484K									
		1	1									
2	AY.120	Q493L	A684V	E702K								
		1	1	1								
45	AY.121	A67V	ΔH69	ΔV70	E96A	S98P	D111N	G142D	S349A	L452W	P479L	N536Y
		2	2	2	1	6	1	17	1	1	1	1
		A684V	S708F	L1063F	D1118Y	V1264L						
		1	2	1	1	1						
115	AY.122	L8F	L18F	T22I	L24S	A27S	A67V	ΔH69	ΔV70	V83F	T95I	D111N
		1	1	2	1	1	1	2	2	1	4	1
		T124I	ΔG142	G142D	ΔV143	ΔY144	Y145D	E224Q	T240I	W258R	L276I	R346I
		1	1	42	1	1	1	1	2	1	1	1
		V433I	S494L	V503F	A522V	T573I	Q613H	A623V	Q677H	S680P	A688V	S704L
		1	1	1	1	3	3	1	1	8	8	3
		T723I	T747I	P812L	K814R	A846V	Q853R	T859I	S939F	A1020S	T1027I	A1078S
		1	2	1	1	1	1	2	1	2	1	2
		V1176F	G1219V	V1228L	M1229I	C1247F	V1264L					
		7	1	1	1	3	1					
1	AY.124.1	A222S										
		1										
3	AY.125	G142D										
		1										
18	AY.126	A67V	ΔH69	ΔV70	E132Q	G142D	N188S	V289L	N532S	D574Y	V622F	S689I
		1	1	1	1	4	1	1	1	1	8	1
		S689R	V1228L	C1243R	C1250F							
		1	8	1	1							
44	AY.127	V6F	L18F	A67V	ΔH69	ΔV70	ΔY144	R246T	E516Q	L821V	D1260Y	
		1	3	1	1	1	1	1	1	8	1	
1	AY.127.2	V860I										

		1										
2	AY.128	S221L	E1150D									
		2	2									
337	BA.1	L5F	H49Y	S71F	G72E	E96V	N196S	ins210IV	L212E	V213P	ins214ETE	R214E
		1	1	2	1	2	1	1	1	1	1	1
		A222V	R237K	F238L	ΔL241	ΔL242	ΔA243	W258C	T323I	S371F	F392L	D427Y
		1	3	1	1	1	1	1	1	1	1	1
		E484S	V511A	N540S	Q613E	Q628R	Q675L	A701V	A899S	Q1005H	P1162L	D1184Y
		1	1	1	1	1	1	1	2	3	1	1
		E1202Q										
		1										
529	BA.1.1	L5F	H49Y	G75V	ΔD138	D138G	ΔP139	ΔF140	ΔL141	Y144V	D215Y	S254F
		4	1	1	1	1	1	1	1	1	1	1
		P272L	T286I	V308L	T430I	G476S	L585F	A623V	H625R	A647V	R683W	T778I
		3	1	1	2	1	1	1	2	2	1	1
		A846G	R847T	L938F	A1015S	A1070S	F1095L	P1112L	R1185H	E1202G	K1205N	V1228L
		1	1	2	1	10	1	1	2	1	3	1
		C1253W										
		6										
71	BA.1.1.1	S46L	ΔE156	ΔF157	R158G	R190G	I931V	N960K				
		1	1	1	1	1	2	1				
157	BA.1.1.15	L5F	P9Q	H49Y	Q52K	S151I	F490V	D574H	A626T	S691P	E702Q	A846V
		1	1	14	1	1	2	1	1	1	3	1
		E1144D	P1162S	I1169V	V1177I	C1247F	D1259Y					
		1	1	1	2	2	1					
1	BA.1.10	S1030A										
		1										
25	BA.1.15	M153I	Q1005H									
		1	1									
14	BA.1.15.1	F238L	P1263L									
		1	2									

16	BA.1.16	V16I	A1020S	V1264M								
		1	1	7								
99	BA.1.17	L5F	T95V	Q218R	P251T	E309Q	G476S	A626S	S691P	A701V	K835I	A879S
		1	1	1	1	1	1	1	1	3	1	1
194	BA.1.17.2	L5F	V127F	ins214ELE	G219D	D253G	E309Q	N450K	V622A	F643L	S810T	Y873H
		3	2	1	2	2	2	1	1	4	1	1
		P1162S	V1228L									
		2	7									
1185	BA.2	L5F	T19L	T33K	I68T	ΔH69	ΔV70	V70G	S98F	N99D	ΔL141	ΔG142
		6	1	1	1	9	9	1	1	3	1	1
		ΔV143	ΔY144	H146Y	K147I	M153T	M153V	L176F	ΔN211	ins212SGR	L212I	ins214EPE
		1	1	1	1	1	1	4	1	1	1	1
		ΔL242	ΔA243	S254F	S255F	A260T	A263T	T323I	R346K	R346T	K356T	R357K
		1	1	2	1	1	1	1	3	1	1	2
		S371L	N556K	T573I	D627G	P631S	V635A	Q677H	N679R	A701V	S704L	T719I
		2	2	2	2	1	1	1	1	5	8	1
		Q755H	R765L	A831V	A846G	S884F	A892V	L938F	S939F	T1009I	A1070V	T1076I
		1	3	1	2	1	2	1	1	1	1	1
		D1084N	R1091L	Q1113R	I1114L	T1117I	G1124V	D1146Y	P1162R	P1162S	D1168G	R1185S
		2	1	1	1	2	1	1	1	4	1	1
		D1199G	D1199N	L1200F	Q1201K	E1202K	E1207D	G1219V	A1222S	C1236F	C1236Y	S1239I
		1	1	1	1	1	2	1	1	2	1	2
		K1266R										
		1										
9	BA.2.1	G219S	L368I	P1143S	N978S							
		1	1	1	1							
1	BA.2.2	I1221T										
		1										
49	BA.2.3	T676I	S691P	M1237T								
		1	1	1								
6	BA.2.3.20	K147N	S640P									

		1	1									
5	BA.2.5	K444R										
		4										
128	BA.2.9	L5F	W64R	ΔK182	ΔQ183	ΔG184	ΔN185	ΔF186	P209L	D215G	L249S	R346K
		1	1	1	1	1	1	1	1	1	1	1
		S371L	L452R	N641Y	L938F	Q1005H	D1163Y	V1264L	K1266E			
		1	1	1	1	1	1	2	1			
2	BA.2.9.3	A93S										
		1										
2	BA.2.9.5	A771S										
		1										
8	BA.2.10	W64R	A623S									
		3	1									
47	BA.2.12.1	H49Y	ΔY145	ΔH146	N164K	W258C	Q613H	S939F				
		1	1	1	1	1	5	1				
3	BA.2.18	G261V										
		1										
45	BA.2.37	A1016V	G1099D									
		1	1									
6	BA.2.65	Y248S										
		1										
1	BA.2.72	D215E										
		1										
1	BA.2.78	R346T	L452M									
		1	1									
48	BA.4	G181R	R346S	F543V	F565I	N658S	A845S	D936N	S939F			
		2	1	1	1	32	2	2	2			
22	BA.4.1	I210T	D215H	H245Y	V341L	R346T	N354D	D389N	K444R	Q675H	K854R	D936Y
		2	1	2	1	4	1	2	1	1	2	1
		V1122M	G1251R									
		1	3									

1	BA.4.1.1	I670V	P1143S									
		1	1									
3	BA.4.4	S704L										
		1										
2	BA.4.6	A845S										
		1										
1	BA.4.7	T1006I										
		1										
6	BA.5	S255F										
		1										
258	BA.5.1	L5F	P9L	V11A	T20I	A67S	D80Y	ΔY144	H146R	R190M	V193L	Y200C
		3	1	1	1	1	1	3	1	1	1	1
		R214L	A222V	T240I	L242H	T250I	P251T	A260T	T274I	E340K	R346S	K444N
		1	1	6	1	1	1	1	1	1	2	3
		V445A	G446V	N460I	E516Q	T572S	T604I	S689I	M740I	L822I	T883I	D936H
		1	1	5	1	1	3	1	1	1	1	1
		S939F	L1034F	V1104L	N1108T	G1124V	D1139H	P1143L	I1183V	G1219V		
		4	1	3	1	2	1	1	2	1		
2	BA.5.1.10	N450D										
		2										
3	BA.5.1.12	A1087S										
		2										
2	BA.5.1.23	ΔY144	V445A	Q1201K								
		2	2	2								
223	BA.5.2	L5F	ΔL10	ΔV11	ΔS12	ΔS13	ΔQ14	ΔC15	ΔV16	ΔN17	S71F	V83L
		1	1	1	1	1	1	1	1	1	1	1
		K97E	D138N	ΔY144	K150N	S151I	K182N	K182Q	G184V	P217S	Q239H	Y248H
		1	2	4	1	2	1	2	1	1	1	2
		S254F	A264D	T299I	R346S	K444M	N450D	G476S	P479H	T547K	S680F	A684V
		1	1	1	1	1	1	1	1	1	1	1
		A688V	S689I	R765L	S940F	Q1071L	Q1071R	H1083Y	C1235F			

		1	1	1	1	1	1	5	1			
139	BA.5.2.1	L5F	V6F	Q14H	N30S	H66R	P82H	K97R	ΔY144	M177I	G181E	G181V
		1	7	1	1	9	1	1	1	2	1	1
		Q183L	V213E	R214H	F220L	E224Q	V445A	Y508H	T572I	A623S	L822F	S939F
		1	2	2	1	1	2	1	1	1	1	4
		Q1071R	E1182Q	D1184G	E1195V							
		1	1	2	2							
11	BF.1	H146Q	V445F	N460I								
		1	1	1								
3	BF.4	ΔY144	A783S									
		1	2									
25	BF.5	P39S	Y248H	A653V	D1084E	D1260Y						
		1	1	1	1	3						
15	BF.7	P9S	V1264L									
		1	2									
1	BF.7.4.2	ΔA27										
		1										
5	BF.10	P272L										
		1										
11	BF.11	N1023Y										
		3										
1	BF.19	ΔY144										
		1										
12	BA.5.2.20	S98F	V642G									
		1	2									
1	BA.5.2.26	R346I										
		1										
30	BE.1	A67T	P793L	A958S	T1273I							
		2	1	2	1							
28	BE.1.1	I101T	R214H	T284I	S297A	E484V	I742V	T747I	S929I			
		1	1	1	1	1	1	2	1			

4	BE.1.1.1	T430I			
		1			
2	BQ.1	R346T	V1040F	M1050I	
		2	2	2	
7	BQ.1.1	P209S	G219C	S939F	M1050I
		1	1	1	1
2	BQ.1.1.11	S494P			
		2			
1	BQ.1.8	L5F			
		1			
7	BA.5.3.3	G181V			
		1			
5	BA.5.6	L5F			
		1			
11	BA.5.9	L249F			
		1			

[a]The most prevalent mutations shown in Table S2 were determined in accordance to the most prevalent mutations of these lineages in this dataset, and as denoted by the CoV-Spectrum [1] (http//cov-spectrum.org/)(date last accessed 23 May 2023) and Outbreak.info [2] (http//outbreak.info/)(date last accessed 23 May 2023) websites.

[b]Total number of sequences that were identified for each lineage in this dataset.

[c]For lineages that were identified in lower frequency in this dataset, the CoV-Spectrum [1] (http//cov-spectrum.org/)(date last accessed 23 May 2023) and Outbreak.info [2] (http//outbreak.info/)(date last accessed 23 May 2023) websites were used to indicate the expected common mutations and deletions.

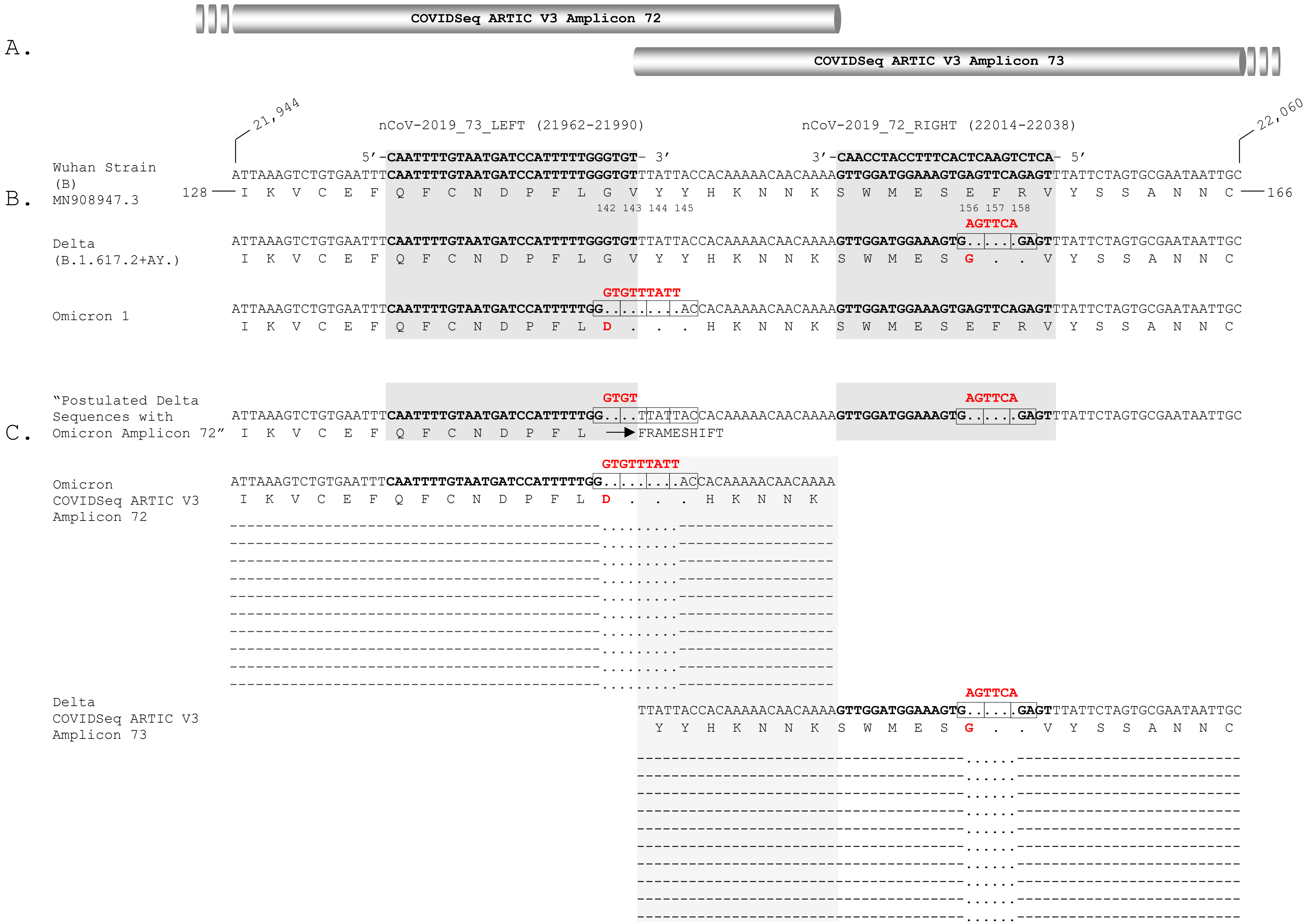

COVIDSeq ARTIC V3 Amplicon 72
A.
COVIDSeq ARTIC V3 Amplicon 73
21,944
nCoV-2019_73_LEFT (21962-21990)
nCoV-2019_72_RIGHT (22014-22038)
22,060
5′-CAATTTTGTAATGATCCATTTTTGGGTGT- 3′
3′-CAACCTACCTTTCACTCAAGTCTCA- 5′
Wuhan Strain (B) MN908947.3
B.
ATTAAAGTCTGTGAATTTCAATTTTGTAATGATCCATTTTTGGGTGTTTATTACCACAAAAACAACAAAAGTTGGATGGAAAGTGAGTTCAGAGTTTATTCTAGTGCGAATAATTGC
128 I K V C E F Q F C N D P F L G V Y Y H K N N K S W M E S E F R V Y S S A N N C 166
142 143 144 145
156 157 158
Delta (B.1.617.2+AY.)
AGTTCA
ATTAAAGTCTGTGAATTTCAATTTTGTAATGATCCATTTTTGGGTGTTTATTACCACAAAAACAACAAAAGTTGGATGGAAAGTG......GAGTTTATTCTAGTGCGAATAATTGC
I K V C E F Q F C N D P F L G V Y Y H K N N K S W M E S G . . V Y S S A N N C
Omicron 1
GTGTTTATT
ATTAAAGTCTGTGAATTTCAATTTTGTAATGATCCATTTTTGG.........ACCACAAAAACAACAAAAGTTGGATGGAAAGTGAGTTCAGAGTTTATTCTAGTGCGAATAATTGC
I K V C E F Q F C N D P F L D . . . H K N N K S W M E S E F R V Y S S A N N C
C.
"Postulated Delta Sequences with Omicron Amplicon 72"
GTGT
AGTTCA
ATTAAAGTCTGTGAATTTCAATTTTGTAATGATCCATTTTTGG....TTATTACCACAAAAACAACAAAAGTTGGATGGAAAGTG......GAGTTTATTCTAGTGCGAATAATTGC
I K V C E F Q F C N D P F L FRAMESHIFT
Omicron COVIDSeq ARTIC V3 Amplicon 72
GTGTTTATT
ATTAAAGTCTGTGAATTTCAATTTTGTAATGATCCATTTTTGG.........ACCACAAAAACAACAAAA
I K V C E F Q F C N D P F L D . . . H K N N K
Delta COVIDSeq ARTIC V3 Amplicon 73
AGTTCA
TTATTACCACAAAAACAACAAAAGTTGGATGGAAAGTG......GAGTTTATTCTAGTGCGAATAATTGC
Y Y H K N N K S W M E S G . . V Y S S A N N C

Figure S1. The postulated sequencing results from ARTIC V3 primer pairs 72 and 73. (A) Graphical representation of the amplicons of ARTIC V3 primer pairs 72 and 73. The gray cylinders indicate the region of amplification of each primer pair and the intermittent cylinders serve to show that the amplicon continues past the nucleotides indicated in (B). **(B)** The region of the SARS-CoV-2 genome where the left primer of primer pair 73 and the right primer of primer pair 72 bind. The region where each primer binds to is highlighted in gray. The numbers above the reference SARS-CoV-2 strain (GenBank: MN908947.3) correspond to the start and end of the genetic region that was isolated to indicate the primer binding sites (numbering based on the MN908947 genome). Directly above the nucleotide sequence of MN908947.3 are the left primer of ARTIC V3 primer pair 73 "nCoV-2019_73_LEFT" (in 5′ to 3′ orientation) and the right primer of ARTIC V3 primer pair 72 (in 3′ to 5′ orientation, complement). The nucleotide positions that they bind to are indicated in the parenthesis. Directly below the nucleotide sequence of MN908947.3 are the translated amino acids of the S-protein, and the numbers on the left and right of this amino acid sequence correspond to the start and end of this isolated region. The second and third genomes indicated in (B) represent sequences of the Delta variant B.1.617.2&AY. and Omicron 1 variant. The red letters indicate the characteristic nucleotide deletions of the Delta and Omicron variants. **(C)** The postulated consensus sequences of ARTIC V3 primer pair 72 in Omicron and ARTIC V3 primer pair 73 in Delta. Directly below the consensus sequences, are representative next generation sequencing fragments from the Omicron consensus sequence shown in (B) that cover the end of the right 72 primer, and representative next generation sequencing fragments from the Delta consensus sequence shown in (B), that cover the beginning of the left 73 primer.

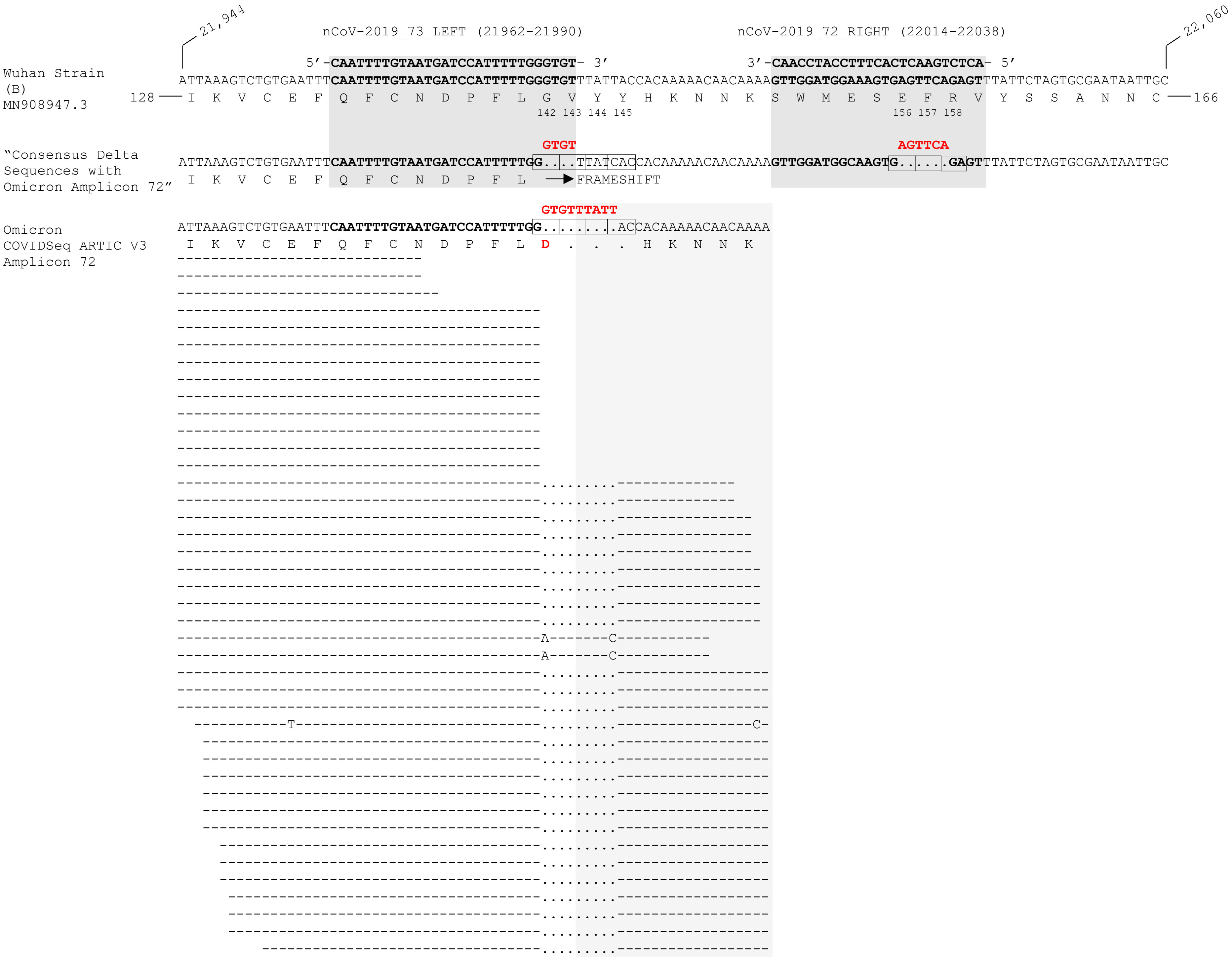
21,944
22,060
nCoV-2019_73_LEFT (21962-21990)
nCoV-2019_72_RIGHT (22014-22038)
5′-CAATTTTGTAATGATCCATTTTTGGGTGT- 3′
3′-CAACCTACCTTTCACTCAAGTCTCA- 5′
Wuhan Strain (B) MN908947.3
ATTAAAGTCTGTGAATTTCAATTTTGTAATGATCCATTTTTGGGTGTTTATTACCACAAAAACAACAAAAGTTGGATGGAAAGTGAGTTCAGAGTTTATTCTAGTGCGAATAATTGC
128 I K V C E F Q F C N D P F L G V Y Y H K N N K S W M E S E F R V Y S S A N N C 166
142 143 144 145
156 157 158
GTGT
AGTTCA
"Consensus Delta Sequences with Omicron Amplicon 72"
I K V C E F Q F C N D P F L
FRAMESHIFT
GTGTTTATT
Omicron COVIDSeq ARTIC V3 Amplicon 72
ATTAAAGTCTGTGAATTTCAATTTTGTAATGATCCATTTTTGG.........ACCACAAAAACAACAAAA
I K V C E F Q F C N D P F L D . . . H K N N K

```
---------------------------------.........------------------
---------------------------------.........------------------
---------------------------------.........------------------
---------------------------------.........------------------
-------------------------------T-.........-----------T------
---------------------------------.........------------------
---------------------------------.........------------------
---------------------------------.........------------------
---------------------------------.........------------------
---------------------------------.........------------------
---------------------------------.........------------------
---------------------------------.........------------------
---------------------------------.........------------------
---------------------------------.........------------------
  -------------------------------.........------------------
  -------------------------------.........------------------
  -------------------------------.........------------------
  -------------------------------.........------------------
  -------------------------------.........------------------
  -------------------------------.........------------------
  -------------------------------.........------------------
  -------------------------------.........------------------
  -------------------------------.........------------------
  -------------------------------.........------------------
  -------------------------------.........---G--------------
  --------------------C----------.........------------------
  -------------------------------.........------------------
  -------------------------------.........------------------
  -------------------------------.........------------------
  -------------------------------.........------------------
  -------------------------------.........------------------
  -------------------------------.........------------------
   ------------------------------.........------------------
   ------------------------------.........------------------
   ------------------------------.........------------------
      ---------------------------.........------------------
      ---------------------------.........------------------
      ---------------------------.........------------------
      ---------------------------.........------------------
      ---------------------------.........------------------
      ---------------------------.........------------------
      ---------------------------.........------------------
      ---------------------------.........------------------
      ---------------------------.........------------------
      ---------------------------.........------------------
      ---------------------------.........------------------
       --------------------------.........------------------
       --------------------------.........------------------
       --------------------------.........------------------
       --------------------------.........------------------
       --------------------------.........------------------
       --------------------------.........------------------
       --------------------------.........------------------
```

Delta
COVIDSeq ARTIC V3
Amplicon 73

```
                                      AGTTCA
TTATCACCACAAAAACAACAAAAGTTGGATGGCAAGTG......GAGTTTATTCTAGTGCGAATAATTGC
  Y  H  H  K  N  N  K  S  W  M  A  S  G  .  .  V  Y  S  S  A  N  N  C
----C--------------------------
----C--------------------------
----C---------------------------
----C---------------------T-----C-----
----C---------------------------C-----
----C---------------------------C-----......-
----C---------------------------C-----......-
----C---------------------------C-----......-
----C---------------------------C-----......----
----C---------------------------C-----......----------
----C---------------------------C-----......----------
----C---------------------------C-----......----------
----C---------------------------C-----......----------
----C---------------------------C-----......----------
----C---------------------------C-----......-----------
----C---------------------------C-----......-----------
----C---------------------------C-----......-----------
----C---------------------------C-----......-----------
----C---------------------------C-----......---------------
----C---------------------------C-----......---------------
----C---------------------------C-----......---------------
----C---------------------------C-----......-----------------
----C---------------------------C-----......------------------
----C---------------------------C-----......------------------
----C---------------------------C-----......------------------
----C---------------------------C-----......------------------
----C---------------------------C-----......------------------
----C---------------------------C-----......------------------
----C---------------------------C-----......------------------
----C---------------------------C-----......------------------
----C---------------------------C-----......------------------
----C---------------------------C-----......------------------
----C---------------------------C-----......------------------
----C---------------------------C-----......------------------
----C---------------------------C-----......------------------
----C---------------------------C-----......------------------
----C---------------------------C-----......--------------------
----C---------------------------C-----......--------------------
----C---------------------------C-----......--------------------
----C---------------------------C-----......---------------------
----C---------------------------C-----......---------------------
----C---------------------------C-----......---------------------
 ---C---------------------------C-----......-------------------------
 ---C---------------------------C-----......-------------------------
 ---C---------------------------C-----......-------------------------
  --C---------------------------C-----......--------------------------
   -C---------------------------C-----......--------------------------
    C---------------------------C-----......--------------------------
    C---------------------------C-----......--------------------------
    C---------------------------C-----......--------------------------
    C---------------------------C-----......--------------------------
    C---------------------------C-----......--------------------------
    C---------------------------C-----......--------------------------
    C--------------------------AC-----......--------------------------
    C---------------------------C-----......--------------------------
```

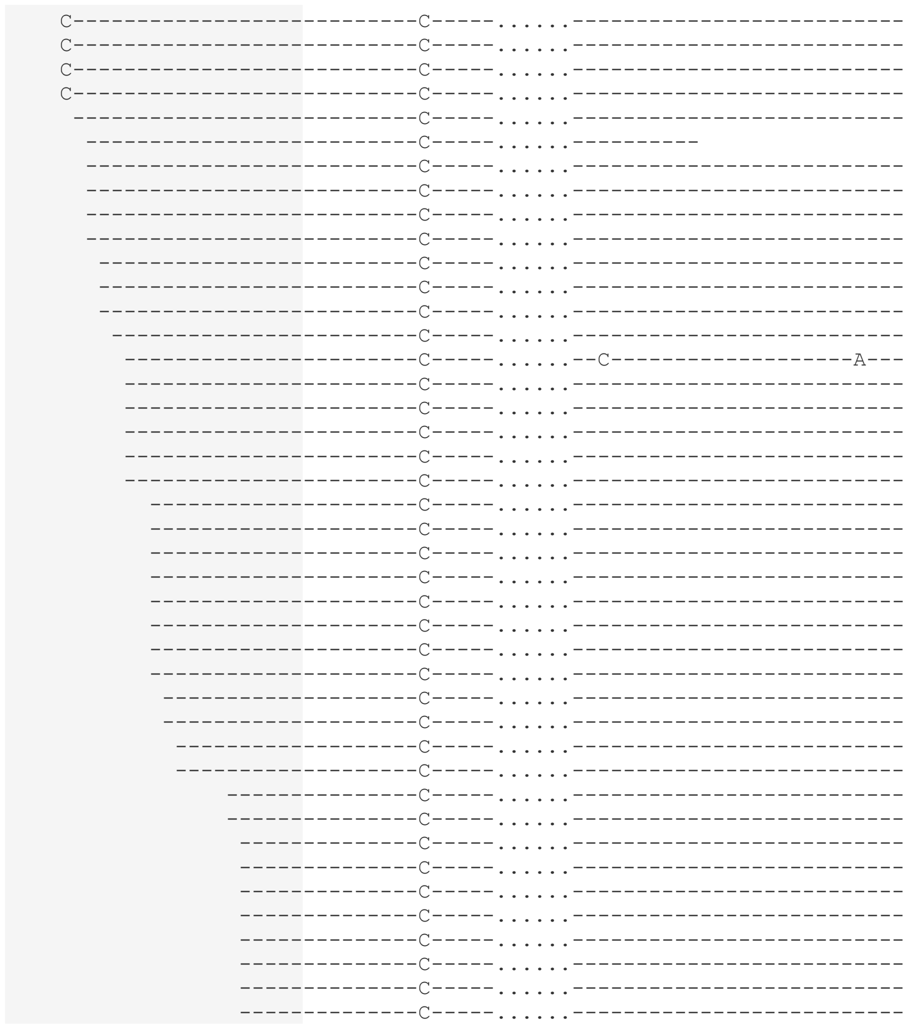

Figure S2. The sequencing results from ARTIC V3 primer pairs 72 and 73 of a Delta sample with a frameshift due to 4-nucleotide deletion at positions 21,987-21,990 . The top genome represents the reference SARS-CoV-2 Wuhan strain MN908947.3 (GenBank ID), showing the region that encompasses the left ARTIC V3 primer 73 and the right ARTIC V3 primer 72. The target recognition sequence of each primer is highlighted in gray. The numbers above the reference SARS-CoV-2 genome correspond to the start and end of the genetic region that was isolated to indicate the primer binding sites (numbering based on the MN908947.3 genome). Directly above the nucleotide sequence of MN908947.3 are the left primer of ARTIC V3 primer pair 73 "nCoV-2019_73_LEFT" (in 5′ to 3′ orientation) and the right primer of ARTIC V3 primer pair 72 (in 3′ to 5′ orientation, complement). The nucleotide positions that the ARTIC V3 primers 72 right and 73 left bind to are indicated in the parenthesis. Directly below the nucleotide sequence of MN908947.3 are the translated amino acids of the S-protein, and the numbers on the left and right of this amino acid sequence correspond to the start and end of this isolated region. The second genome indicated below MN908947.3 represents the consensus sequence of the AY.4.2 sample. The red characters indicate deleted nucleotides and corresponding amino acid changes of the consensus sequence of AY.4.2 relative to the MN908947.3 sequence. Directly below the AY.4.2 consensus sequence, are representative next generation sequencing fragments that cover the end of the right ARTIC V3 primer 72, and representative next generation sequencing fragments from that cover the beginning of the left ARTIC V3 primer 73.

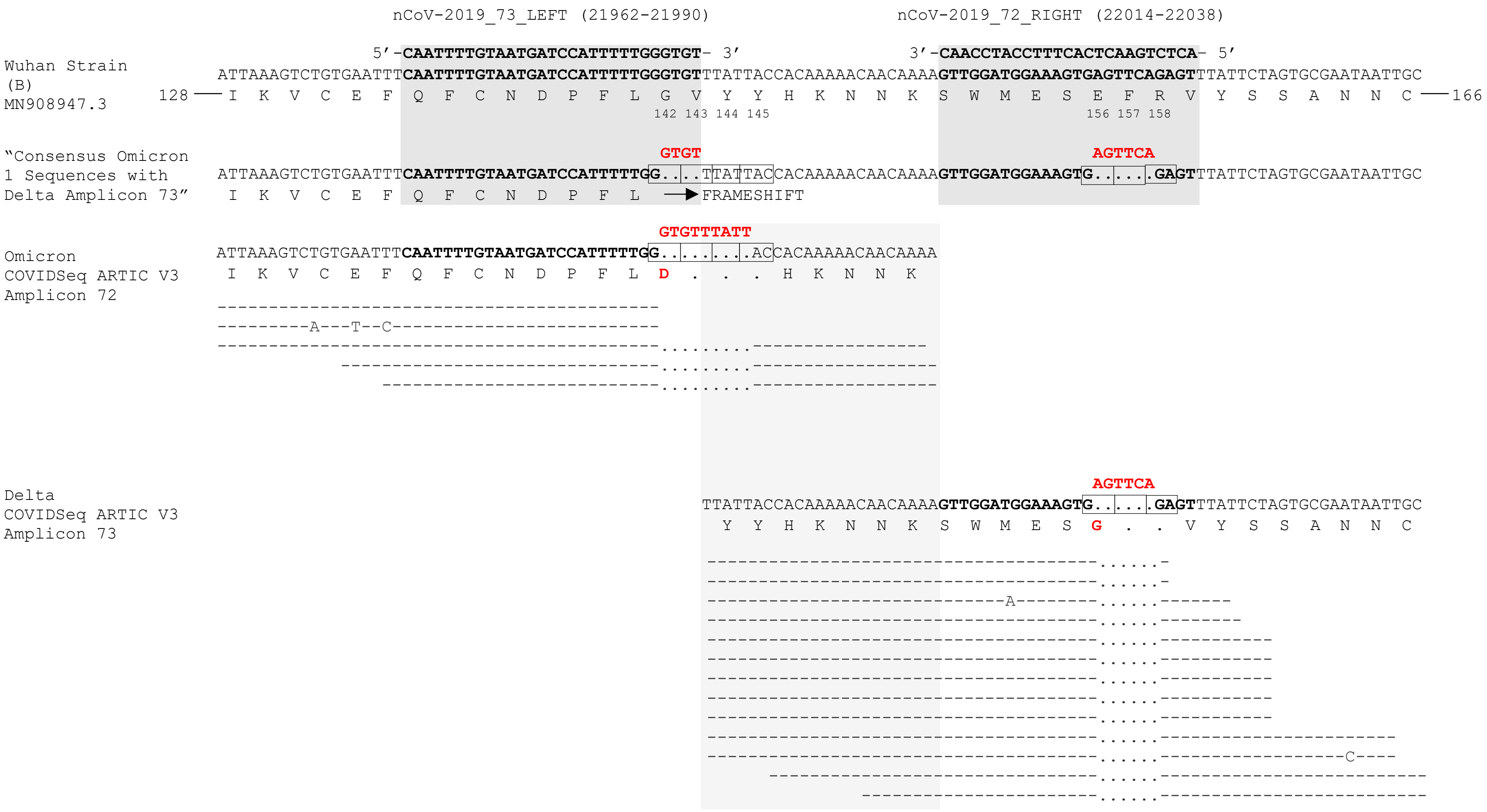

Figure S3. The sequencing results from ARTIC V3 primer pairs 72 and 73 of an Omicron 1 sample with a frameshift due to 4-nucleotide deletion at positions 21,987-21,990. The top genome represents the reference SARS-CoV-2 Wuhan strain MN908947.3 (GenBank ID), showing the region that encompasses the left ARTIC V3 primer 73 and the right ARTIC V3 primer 72. The target recognition sequence of each primer is highlighted in gray. The numbers above the reference SARS-CoV-2 genome correspond to the start and end of the genetic region that was isolated to indicate the primer binding sites (numbering based on the MN908947.3 genome). Directly above the nucleotide sequence of MN908947.3 are the left primer of ARTIC V3 primer pair 73 "nCoV-2019_73_LEFT" (in 5' to 3' orientation) and the right primer of ARTIC V3 primer pair 72 (in 3' to 5' orientation, complement). The nucleotide positions that the ARTIC V3 primers 72 right and 73 left bind to are indicated in the parenthesis. Directly below the nucleotide sequence of MN908947.3 are the translated amino acids of the S-protein, and the numbers on the left and right of this amino acid sequence correspond to the start and end of this isolated region. The second genome indicated below MN908947.3 represents the consensus sequence of a BA.1. The red characters indicate deleted nucleotides and corresponding amino acid changes of the consensus sequence of BA.1 relative to the MN908947.3 sequence. Directly below the BA.1 consensus sequence, are representative next generation sequencing fragments that cover the end of the right ARTIC V3 primer 72, and representative next generation sequencing fragments from that cover the beginning of the left ARTIC V3 primer 73.

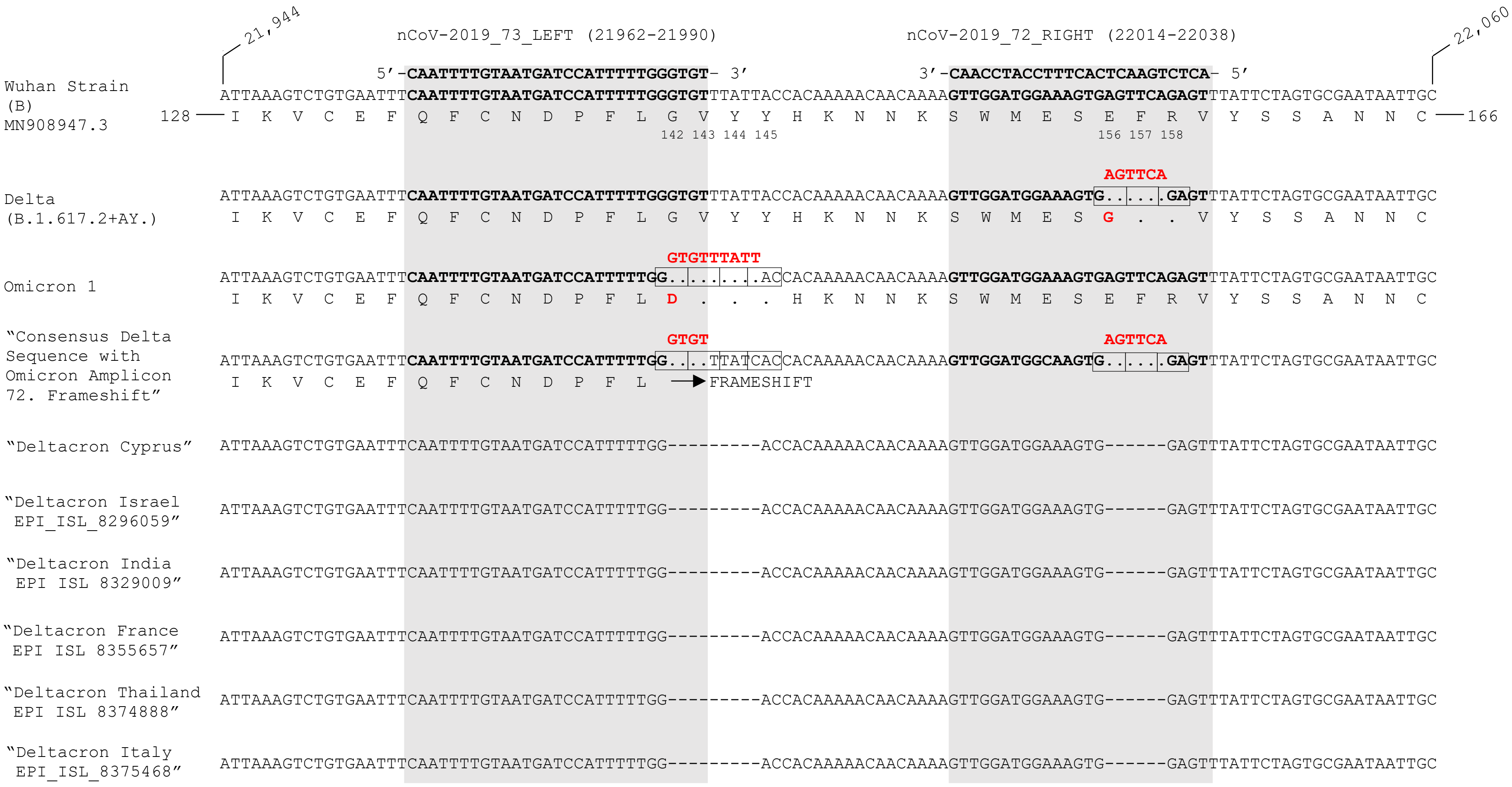

Figure S4. Alignment of representative Deltacron sequences from Cyprus and other countries. The top genome represents the reference SARS-CoV-2 Wuhan strain MN908947.3 (GenBank ID), showing the region that encompasses the left ARTIC V3 primer 73 and the right ARTIC V3 primer 72. The target recognition sequence of each primer is highlighted in gray. The numbers above the reference SARS-CoV-2 genome correspond to the start and end of the genetic region that was isolated to indicate the primer binding sites (numbering based on the MN908947.3 genome). Directly above the nucleotide sequence of MN908947.3 are the left primer of ARTIC V3 primer pair 73 "nCoV-2019_73_LEFT" (in 5′ to 3′ orientation) and the right primer of ARTIC V3 primer pair 72 (in 3′ to 5′ orientation, complement). The nucleotide positions that the ARTIC V3 primers 72 right and 73 left bind to are indicated in the parenthesis. Directly below the nucleotide sequence of MN908947.3 are the translated amino acids of the S-protein, and the numbers on the left and right of this amino acid sequence correspond to the start and end of this isolated region. The second and third genomes represent sequences of the Delta variant B.1.617.2&AY. and Omicron 1 variant. The red letters indicate the characteristic nucleotide deletions of the Delta and Omicron variants. The fourth genome indicated below MN908947.3 represents the consensus sequence of a Delta variant identified in our dataset with the frameshift resulting from a four-nucleotide deletion at positions 21,987-21,990 due to the ARTIC V3 right 72 primer amplifying Omicron 1, while ARTIC V3 left 73 primer amplifying Delta. The sequences below the fourth indicate Deltacron sequences from Cyprus and other countries, which do not contain the aforementioned four-nucleotide deletion, and as such do not have the frameshift.

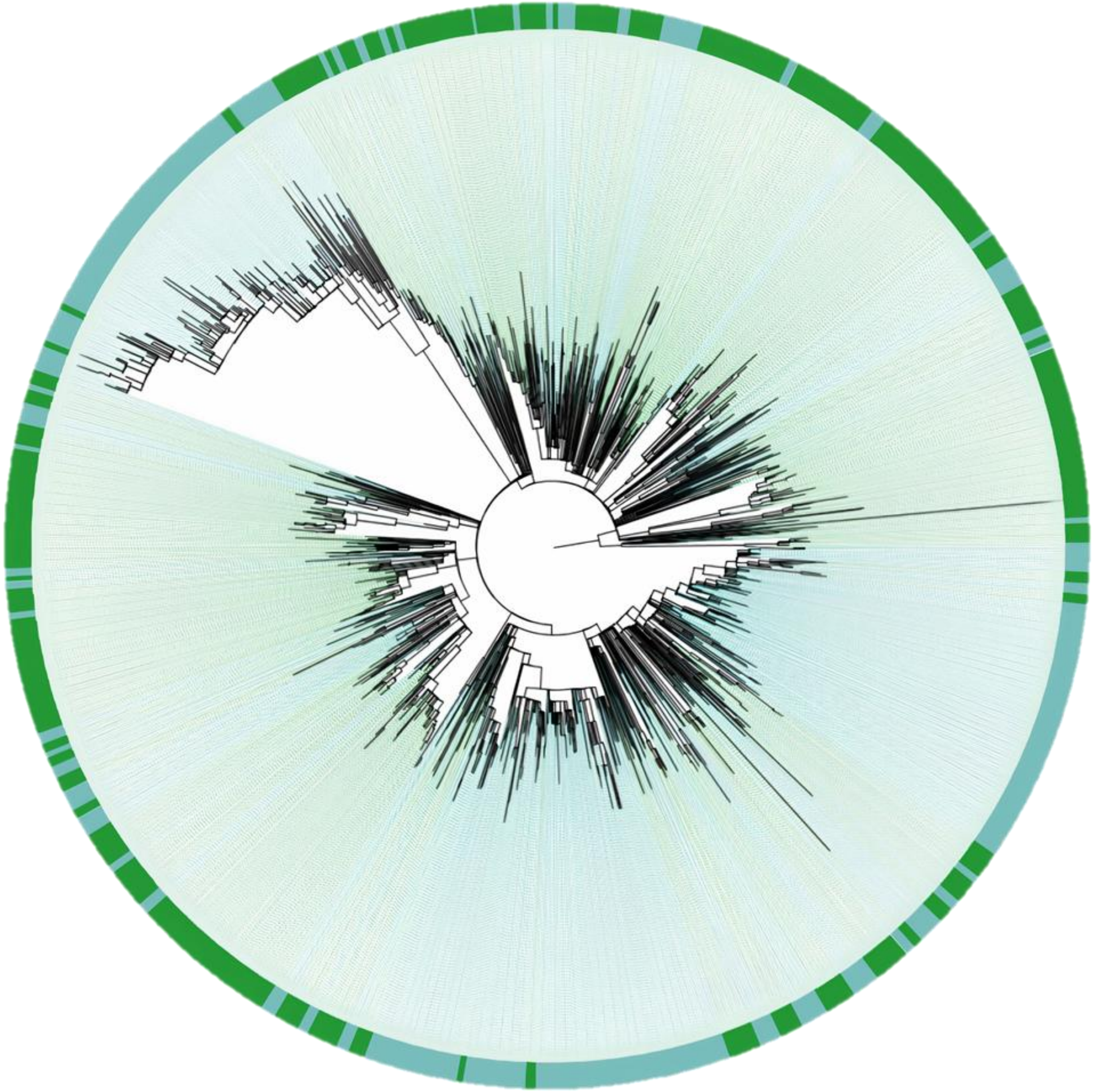

Figure S5. Maximum likelihood phylogenetic tree construction encompassing Delta variants from the fourth and fifth waves of SARS-CoV-2 infection in Cyprus. The Delta variant sequences highlighted in teal are predominantly from the fourth wave (previous study [3]), which present a more condensed clustering. The Delta variant sequences in green are primarily from the fifth wave of SARS-CoV-2 (current study), which present a more interspersed clustering.

References

1. Chen, C.; Nadeau, S.; Yared, M.; Voinov, P.; Xie, N.; Roemer, C.; Stadler, T. CoV-Spectrum: analysis of globally shared SARS-CoV-2 data to identify and characterize new variants. *Bioinformatics* **2022**, *38*, 1735–1737, doi:10.1093/bioinformatics/btab856.
2. Gangavarapu, K.; Latif, A.A.; Mullen, J.L.; Alkuzweny, M.; Hufbauer, E.; Tsueng, G.; Haag, E.; Zeller, M.; Aceves, C.M.; Zaiets, K.; et al. Outbreak.info genomic reports: scalable and dynamic surveillance of SARS-CoV-2 variants and mutations. *Nat. Methods* **2023**, *20*, 512–522, doi:10.1038/s41592-023-01769-3.
3. Chrysostomou, A.C.; Vrancken, B.; Haralambous, C.; Alexandrou, M.; Aristokleous, A.; Christodoulou, C.; Gregoriou, I.; Ioannides, M.; Kalakouta, O.; Karagiannis, C.; et al. Genomic Epidemiology of the SARS-CoV-2 Epidemic in Cyprus from November 2020 to October 2021: The Passage of Waves of Alpha and Delta Variants of Concern. *Viruses* **2022**, *15*, 108, doi:10.3390/v15010108.
